# A fluorophore's electron-deficiency does matter in designing high-performance near-infrared fluorescent probes†

**DOI:** 10.1039/d0sc04411c

**Published:** 2020-09-21

**Authors:** Xue-Xiang Zhang, Huan Qi, Ya-Lan Liu, Song-Qiu Yang, Peng Li, Yan Qiao, Pei-Yu Zhang, Shu-Hao Wen, Hai-long Piao, Ke-Li Han

**Affiliations:** State Key Laboratory of Molecular Reaction Dynamics, Dalian Institute of Chemical Physics, Chinese Academy of Sciences Dalian 116023 P. R. China klhan@dicp.ac.cn; CAS Key Laboratory of Separation Science for Analytical Chemistry, Dalian Institute of Chemical Physics, Chinese Academy of Sciences Dalian 116023 P. R. China hpiao@dicp.ac.cn; Institute of Molecular Sciences and Engineering, Shandong University Qingdao 266237 P. R. China; Department of Pathophysiology, Basic Medical College of Zhengzhou University Zhengzhou 450001 P. R. China; Shenzhen Jingtai Technology Co., Ltd Floor 4, No. 9, Hualian Industrial Zone, Dalang Street, Longhua District Shenzhen P. R. China peiyu.zhang2018@gmail.com

## Abstract

The applications of most fluorescent probes available for Glutathione *S*-Transferases (GSTs), including **NI3** which we developed recently based on 1,8-naphthalimide (**NI**), are limited by their short emission wavelengths due to insufficient penetration. To realize imaging at a deeper depth, near-infrared (NIR) fluorescent probes are required. Here we report for the first time the designing of NIR fluorescent probes for GSTs by employing the NIR fluorophore **HCy** which possesses a higher brightness, hydrophilicity and electron-deficiency relative to **NI**. Intriguingly, with the same receptor unit, the **HCy**-based probe is always more reactive towards glutathione than the **NI**-based one, regardless of the specific chemical structure of the receptor unit. This was proved to result from the higher electron-deficiency of **HCy** instead of its higher hydrophilicity based on a comprehensive analysis. Further, with caging of the autofluorescence being crucial and more difficult to achieve *via* photoinduced electron transfer (PET) for a NIR probe, the quenching mechanism of **HCy**-based probes was proved to be PET for the first time with femtosecond transient absorption and theoretical calculations. Thus, **HCy2** and **HCy9**, which employ receptor units less reactive than the one adopted in **NI3**, turned out to be the most appropriate NIR probes with high-sensitivity and little nonenzymatic background noise. They were then successfully applied to detecting GST in cells, tissues and tumor xenografts *in vivo*. Additionally, unlike **HCy2** with a broad isoenzyme selectivity, **HCy9** is specific for GSTA1-1, which is attributed to its lower reactivity and the higher effectiveness of GSTA1-1 in stabilizing the active intermediate *via* H-bonds based on docking simulations.

## Abstract 2

An abnormal and intriguing phenomenon that the fluorophore's electron-deficiency could affect a probe's performance is now revealed for the first time.

## Graphical abstract

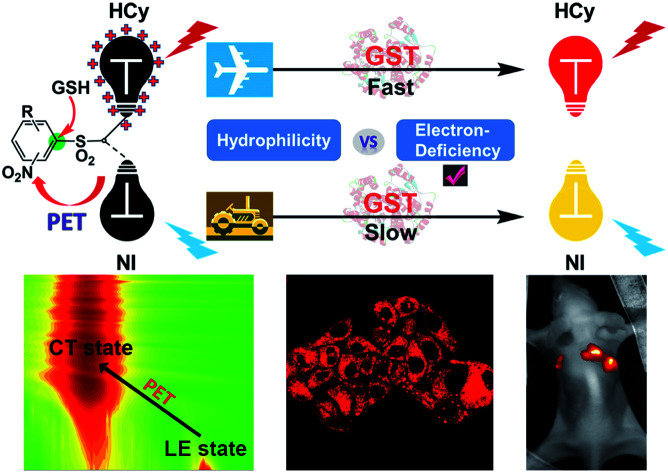

## Introduction

Three subclasses of cytosolic Glutathione *S*-Transferases (GSTs, EC 2.5.1.18), GSTA, GSTM and GSTP are regularly overexpressed in tumor cells, especially in those with drug-resistance.^1–5^ With little toxicity, high sensitivity, a fast and convenient detection process, and facile modification, more and more attention has been drawn to small molecule fluorescent probes.^6–12^ Recently, with the 1,8-naphthalimide (**NI**) scaffold, we have developed a two-photon fluorescent probe **NI3** for GSTs after an elaborate investigation into the structure–activity relationships between nonenzymatic or enzymatic reactivities and the effective electrophilicity of the receptor unit.^13^ Despite **NI**'s two-photon absorptivity, its short emission wavelength impeded more practical applications such as *in vivo* imaging. It would be desirable that both excitation and emission wavelengths are in the near-infrared (NIR) region (650–900 nm) due to deeper skin and tissue penetration, lower background autofluorescence interference, and less phototoxicity.^14–19^ In addition, NIR fluorophores usually possess a higher molar extinction coefficient *ε*, and as can be seen in eqn (1), this property is conducive to superior signal sensitivity (refer to deduction I in the ESI†).1
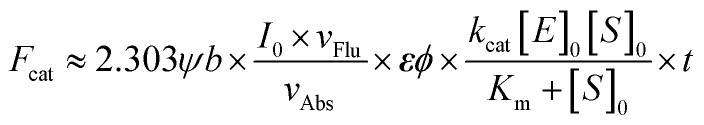


Hence, (*E*)-2-(2-(6-hydroxy-2,3-dihydro-1*H*-xanthen-4-yl)vinyl)-3,3-dimethyl-1-ethyl-3*H*-indol-1-ium iodide (**HCy**) was selected as the fluorophore in light of its NIR character, high brightness (*εφ*) and favorable photostability.^20,21^ What's more, a systematic study on whether and how replacement of the fluorophore could affect a probe's recognition behavior is lacking. Herein, to address this very issue and to develop highly sensitive NIR fluorescent probes for GSTs with negligible background noise for the first time, receptor units adopted in **NI**-based probes were linked with **HCy** to produce the NIR probes, which unexpectedly appeared to be more reactive than **NI**-based ones. Subsequent studies on the hydrophilicity and the electron-deficiency of the fluorophore demonstrated that the latter is the origin of the higher reactivity. Meanwhile, the fluorescence caging mechanism of **HCy**-based probes was inspected with femtosecond transient absorption spectra and theoretical calculations. Thus, instead of 4-cyano-2-nitro-benzenesulfonyl adopted in **NI3**, **HCy2** and **HCy9** with more inert receptor units turned out to be the most appropriate NIR probes for practical applications, including GST imaging in cells, tissues and even tumor xenografts in nude mice. In addition, the different isoenzyme selectivities of these two probes and the specificity of **HCy9** for GSTA1-1 were discussed according to the docking simulations.

## Results and discussion

### Design, synthesis and evaluation of **HCy**-based NIR probes *in vitro*

**Fig. 1:**
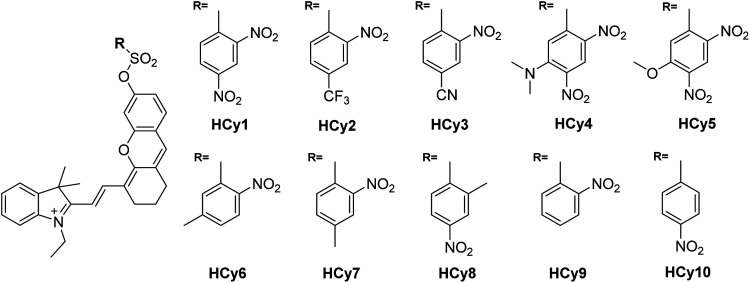
Chemical structures of probes **HCy1–10**.

**Fig. 2:**
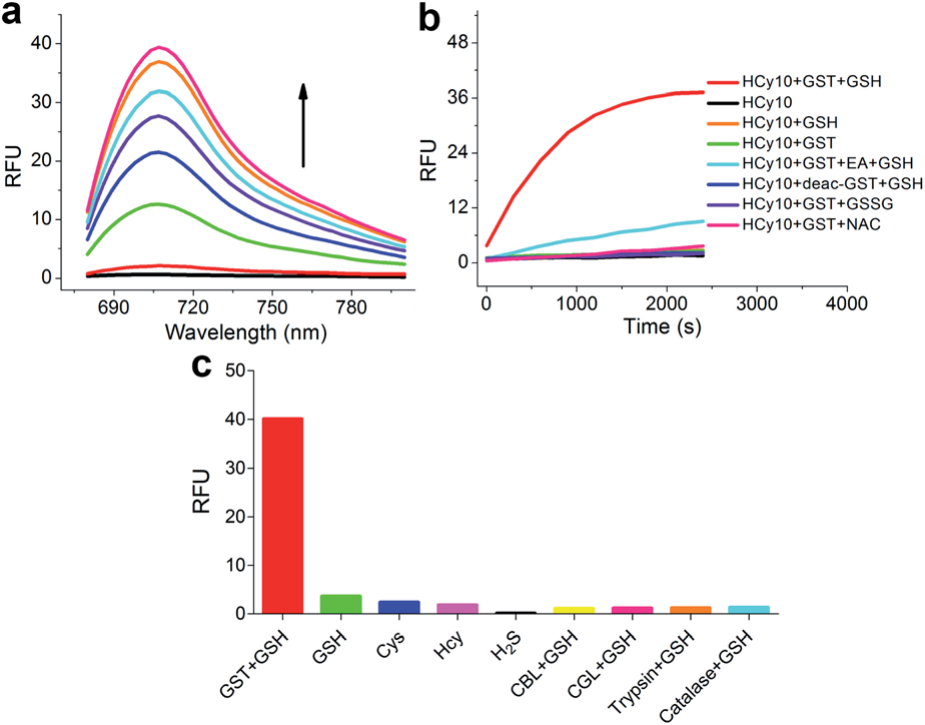
(a) Fluorescence spectral changes of **HCy10** (20 μM) in HEPES buffer (20 mM, 0.5% DMSO, pH 7.4) upon addition of GSTs (12.5 μg mL^−1^) over the course of *ca.* 30 min at 37 °C in the presence of GSH (1 mM). *λ*_ex_ = 650 nm. (b) Inspection of the origin of the fluorescence increase in (a). EA = ethacrynic acid (before addition of GSH and **HCy10** sequentially, GSTs were preincubated with 200 μM EA for 30 min); deac-GST = deactivated GSTs (12.5 μg mL^−1^) by preprocessing at 100 °C for 10 min; GSSG = oxidized glutathione (1 mM); NAC = *N*-acetylcysteine (1 mM). *λ*_ex/em_ = 650/700 nm. (c) Selectivity test of **HCy10** (20 μM) towards GST activity over reactive sulfur species and other related biological enzymes. Cys = l-cysteine (1 mM); Hcy = l-homocysteine (1 mM); H_2_S was produced by Na_2_S (1 mM) solution; CBL = cystathionine β-lyase (12.5 μg mL^−1^); CGL = cystathionine γ-lyase (12.5 μg mL^−1^). Data were obtained after incubation at 37 °C in HEPES buffer (20 mM, 0.5% DMSO, pH 7.4) for 1 h.

As in the design of **NI**-based probes, all corresponding receptor units were adopted and attached to **HCy** to give NIR probes **HCy1–10** (Fig. 1 and S1†). The synthesis procedures for compounds **HCy1–10** and corresponding characterization using NMR spectroscopy and mass spectrometry are provided in the ESI.† First, we examined if the sequence of the local electrophilicity *ω*_*k*_^13,22,23^ was influenced by the replacement of the fluorophore from **NI** to **HCy**, and the results showed that substantial agreement was reached between these two series (Table S1†), demonstrating that the receptor unit of **HCy**-based probes still dominates its reactivity (see also Fig. S2†). Then, these probes were subjected to GST detection *in vitro*, and as shown in Fig. S3,†**HCy1** and **HCy3–5** manifested bright fluorescence in the presence of GSH with no need for GSTs while **HCy2** and **HCy6–10** exhibited responses to GSTs with little nonenzymatic background noise. For the latter ones, as a representative, the fluorescence intensity of **HCy10** showed a 20-fold increase at 710 nm in *ca.* 30 min upon encountering GSTs (Fig. 2a). Further, a series of contrast experiments proved that this enhancement arising from GSH addition catalyzed by GST activity (Fig. 2b), and specifically ethacrynic acid (EA, a common inhibitor for various GSTs^24^) could significantly suppresse the fluorescence increase by inhibiting these activities. Subsequent selectivity experiments illustrated the probe's specificity for GST activity (Fig. 2c). To confirm that **HCy**-based probes share the same detection mechanism with **NI**-based ones (Scheme S1†), UPLC-MS and spectra comparative analyses were implemented, and the results have revealed this conclusion (Fig. S4 and S5†).

### Exploring the cause of the different behaviors (both nonenzymatic and enzymatic) between **HCy**-based and **NI**-based probes

Interestingly, although the detection mechanism doesn't change with the replacement of the fluorophore from **NI** to **HCy**, some differences do appear. In the **NI**-series, only **NI1** and **NI5** with the most and the second most electrophilic groups, respectively, as the receptor unit showed unacceptable nonenzymatic background noise,^13^ while in the **HCy**-series, not only **HCy1** and **HCy5** but also **HCy3** and **HCy4** did (Fig. S3†). Notably, even **HCy10**, the receptor unit of which is a relatively inert one, displayed some perceivable nonenzymatic reactivity (Fig. S3j†), which didn't happen in the case of **NI10**.^13^ Then, how come? Inspired by eqn (1), we can obtain eqn (2) describing the nonenzymatic reaction signal. The above results are equivalent to the fact that the initial slope of an *F*–*t* curve for a **HCy**-based probe seems to be larger than the one for a **NI**-based probe bearing the same receptor unit. Thus, one may suspect two causes. One is the higher brightness (*εφ*) of **HCy** (28 440 M^−1^ cm^−1^)^20^ relative to **NI**'s (660 M^−1^ cm^−1^),^25^ which equivalently means amplifying the signal of a certain reaction behavior. The other one is the higher reactivity (*k*_nonc_) of **HCy**-based probes. To identify if the second one hits the bull's eye, kinetic study on nonenzymatic reactions was conducted. As shown in Table 1, **HCy**-based probes are indeed more prone to reacting with GSH than **NI**-based ones, regardless of the specific receptor unit. These results demonstrated that a probe's reactivity is not only related to the receptor unit but also to the fluorophore. So, when regarding the reactivity, what causes the difference between **HCy** and **NI**?2
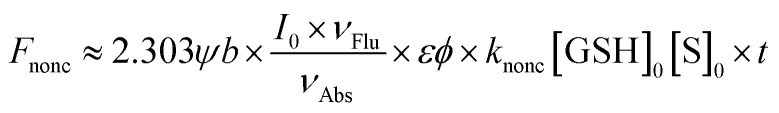


**Table 1:** Apparent second-order rate constants *k*_nonc_ (unit: s^−1^ M^−1^) for nonenzymatic reactions of probes **HCy2–4** and **NI2–4** with GSH^a^

	*k*_nonc_ (**HCy**-)	*k*_nonc_ (**NI**-)^b^	*R*_1_ c
2	1.189 ± 0.116	0.025 ± 0.002	48.2 ± 8.5
4	3.446 ± 0.153	0.256 ± 0.042	13.9 ± 2.8
3	5.737 ± 0.690	0.960 ± 0.160	6.3 ± 1.8

a The *k*_nonc_ data for **HCy10** were 0.002 ± 0.000 s^−1^ M^−1^ while for **NI10** they were undetectable. The data for **HCy1** and **HCy5** were not determined because of their excessively fast reaction rates with GSH. The data for other **HCy**-based probes were undetectable.

b The data were drawn from ref. 13.

c *R*_1_ = *k*_nonc_ (**HCy**-)/*k*_nonc_ (**NI**-), characterizing the enlargement factor of the reaction rate when replacing the fluorophore **NI** with a more hydrophilic and electrophilic one, **HCy**. The list was sorted based on the mean values of *R*_1_ from the highest to the lowest.

After careful consideration, one can realize two possible rational reasons. The first one is the higher hydrophilicity of **HCy** due to its net positive charge, which is conducive to encountering similarly hydrophilic GSH molecules. The second one is the higher electron-deficiency of **HCy** due to the same positive charge, which favors the nucleophilic attack of GSH on the electrophilic center *via* the electronic effect and maybe electrostatic attraction (GSH bearing negative charges) as well. To discern which one accounts for this phenomenon, several parameters based on rate constants *k*_nonc_, octanol–water partition coefficients log *P* and the local electrophilicity *ω*_*k*_, respectively, were defined and inspected. Therein, the log *P* values of **HCy**-based probes calculated with the well-known ALOGPS 2.1 program^26–28^ are truly smaller irrespective of the specific receptor unit, indicating their higher hydrophilicity (Table 2). However, if the hydrophilicity were responsible, when altering the receptor unit, the parameter *R*_2_, or rather, *R*_3_ (refer to deduction II in the ESI†), which characterize the enlargement factor of hydrophilicity when changing the fluorophore from **NI** to **HCy**, should keep pace with *R*_1_ which characterizes the enlargement factor of the reaction rate when changing the fluorophore from **NI** to **HCy**. And as shown in Tables 1 and 2, the sequence **2** > **4** > **3** in terms of *R*_1_ is completely opposite to the **3** > **4** > **2** in terms of *R*_2_ or *R*_3_, thus eliminating the possibility of hydrophilicity being the reason. The log *P* values calculated with another widely-accepted program, XLOGP3,^29^ gave the same conclusion (Table S2†). What about the second reason, namely the electron-deficiency of the fluorophore? By a rough approximation and evaluation, the changes of *ω*_*k*_ were examined. As indicated in Table 3, **HCy**-based probes showed larger local electrophilicity than **NI**-based ones despite the specific receptor unit, demonstrating their higher tendency to be attacked by the nucleophile GSH and thus higher reactivity, and their higher electron affinity consolidates this conclusion (Table S1†). Furthermore, the changes of the parameter *R*_4_, which characterizes the enlargement factor of *ω*_*k*_ when changing the fluorophore from **NI** to **HCy**, are along the ascending order of *ω*_*k*_ and in line with that of *R*_1_ (Tables 1 and 3), again manifesting that **HCy**'s higher electron-deficiency brings about the probes' higher reactivity. And the fact that the more electrophilic (*i.e. ω*_*k*_) the receptor unit is, the less the enlargement factor of *ω*_*k*_ (*i.e. R*_4_) or *k*_nonc_ (*i.e. R*_1_) is, could be interpreted and comprehended readily as follows. In a probe molecule, just as demonstrated above, either a more electrophilic receptor unit or a more electron-deficient fluorophore will induce its higher reactivity with GSH. Hence, when a probe is endowed with a more electrophilic receptor unit, the additional elecrtrophilic effect brought about by the introduction of a more electron-deficient fluorophore seems not that notable, sort of like the law of diminishing marginal utility in economics (refer to deduction III in the ESI† for a little more strict proof). Additionally, previous literature^7^ reported three probes for GSTs, DNs-AcRh, DNs-Coum and DNs-CV, adopting the same DN group as the receptor unit with different fluorophores (Fig. S7†). In that delicate work the authors proved the sequence of DNs-AcRh < DNs-CV < DNs-Coum in terms of hydrophilicity with a tactfully designed experiment calling for the surfactant Triton X-100. However, they didn't notice the sequence of DNs-AcRh > DNs-Coum > DNs-CV in terms of both *k*_nonc_ and *k*_cat_ (Tables S3 and S4†), and neither did they address the reason therein. Actually, the information just mentioned could afford the rejection of hydrophilicity as the reason. Notably, as shown in Tables S3,† the calculated log *P* values for these three probes with both ALOGPS 2.1 and XLOGP3 programs agree quite well with the reported experimental results, indicating the reliability of this calculation method. In addition, the sequence of DNs-AcRh > DNs-Coum > DNs-CV in terms of *ω*_*k*_ is consistent with the one related to *k*_nonc_ or *k*_cat_ (Tables S3, S4 and Fig. S8†). Taken together, all these results corroborate that it is not the higher hydrophilicity but the higher electron-deficiency of the fluorophore that makes the probe more reactive.

**Table 2:** Octanol–water partition coefficients (log *P*) for probes **HCy2–4** and **NI2–4** calculated with the ALOGPS 2.1 program^26–28^ and comparisons of hydrophilicity

	log *P* (**NI**-)	log *P* (**HCy**-)	*R*_2_ a	*R*_3_ b
3	2.82	2.00	1.41	2.25
4	3.22	2.46	1.31	2.13
2	3.61	3.01	1.20	1.82

a *R*_2_ = log *P* (**NI**-)/log *P* (**HCy**-).

b *R*_3_ = *x*_w_ (**HCy**-)/*x*_w_ (**NI**-) = (1 + *P* (**NI**-))/(1 + *P* (**HCy**-)), refer to deduction II in the ESI for more about the parameter *x*_w_. The list was sorted based on the values of *R*_2_ or *R*_3_ from the highest to the lowest.

**Table 3:** Comparison between **HCy**-based and **NI**-based probes in terms of the local electrophilicity *ω*_*k*_ (unit: eV)

	*ω*_*k*_ (**HCy**-)	*ω*_*k*_ (**NI**-)^a^	*R*_4_ b
10	1.106	0.414	2.673
2	1.349	0.517	2.611
4	1.776	0.680	2.610
3	1.947	0.785	2.481
1	2.092	0.910	2.301

a The data were drawn from ref. 13.

b *R*_4_ = *ω*_*k*_ (**HCy**-)/*ω*_k_ (**NI**-). The list was sorted based on the values of *R*_4_ from the highest to the lowest.

Will *k*_cat_ undergo a corresponding rise when replacing the fluorophore **NI** with **HCy**, just as in the case of *k*_nonc_? To check this, a kinetic study on enzymatic reactions was executed (Tables S5–S7†), and the answer was found to be yes. For instance, regarding GSTA1-1, the *k*_cat_ for **NI2** and **HCy2** are, respectively, 0.158 ± 0.009 and 0.408 ± 0.023 s^−1^, and for **NI9** and **HCy9** they are, respectively, 0.010 ± 0.001 and 0.099 ± 0.006 s^−1^.^13^ The larger enhancement factor of *k*_nonc_ than of *k*_cat_ (*e.g.* for **NI2** → **HCy2**, the corresponding numbers are 48.2 and 2.6, respectively) implies that the stronger enzymatic reactivity of **HCy**-based probes stems from their stronger nonenzymatic reactivity. Besides, the *K*_m_ values of **HCy**-based probes are universally smaller than those of **NI**-based ones,^13^ indicating their stronger binding ability with GSTs. Consequently, the larger *k*_cat_ and smaller *K*_m_ make **HCy**-based probes more prone to recognizing and detecting GSTs to the extent that the catalytic efficiency *k*_cat_/*K*_m_ of **HCy9** for GSTA1-1 is 0.124 ± 0.021 s^−1^ μM^−1^, a value comparable to that of **NI3** (0.103 ± 0.011 s^−1^ μM^−1^),^13^ the receptor unit of which is a more electrophilic one, implying **HCy9**'s practical detection ability for GSTA in cells (*vide infra*). Another noteworthy result is that the susceptibility of *k*_nonc_ (6.44) towards *ω*_*k*_ is still larger than that of *k*_cat_ (0.72) (Fig. S9†), analogous to the case in **NI**-based probes,^13^ thus affording the space for selecting practical probes with both high sensitivity and low nonenzymatic background noise. Noticeably, **HCy10** and **HCy8** exhibited inappropriately low enzymatic reactivity (Fig. S9 and Table S5†), indicating the essential role the *o*-NO_2_ group plays in GST catalysis, in agreement with previous literature.^7,13,30^ An overall consideration of the sensitivity and background noise (Fig. S6†) showed **HCy2** and **HCy9** as the outstanding probes for potential practical applications in the following sections. This result overturns 4-cyano-2-nitro-benzenesulfonyl as the master key for all practical GST probes, although it was proved to be the most appropriate receptor unit for **AcRh**-based^8^ and **NI**-based^13^ probes.

### Insight into the caging of autofluorescence of intact NIR probes

**Fig. 3:**
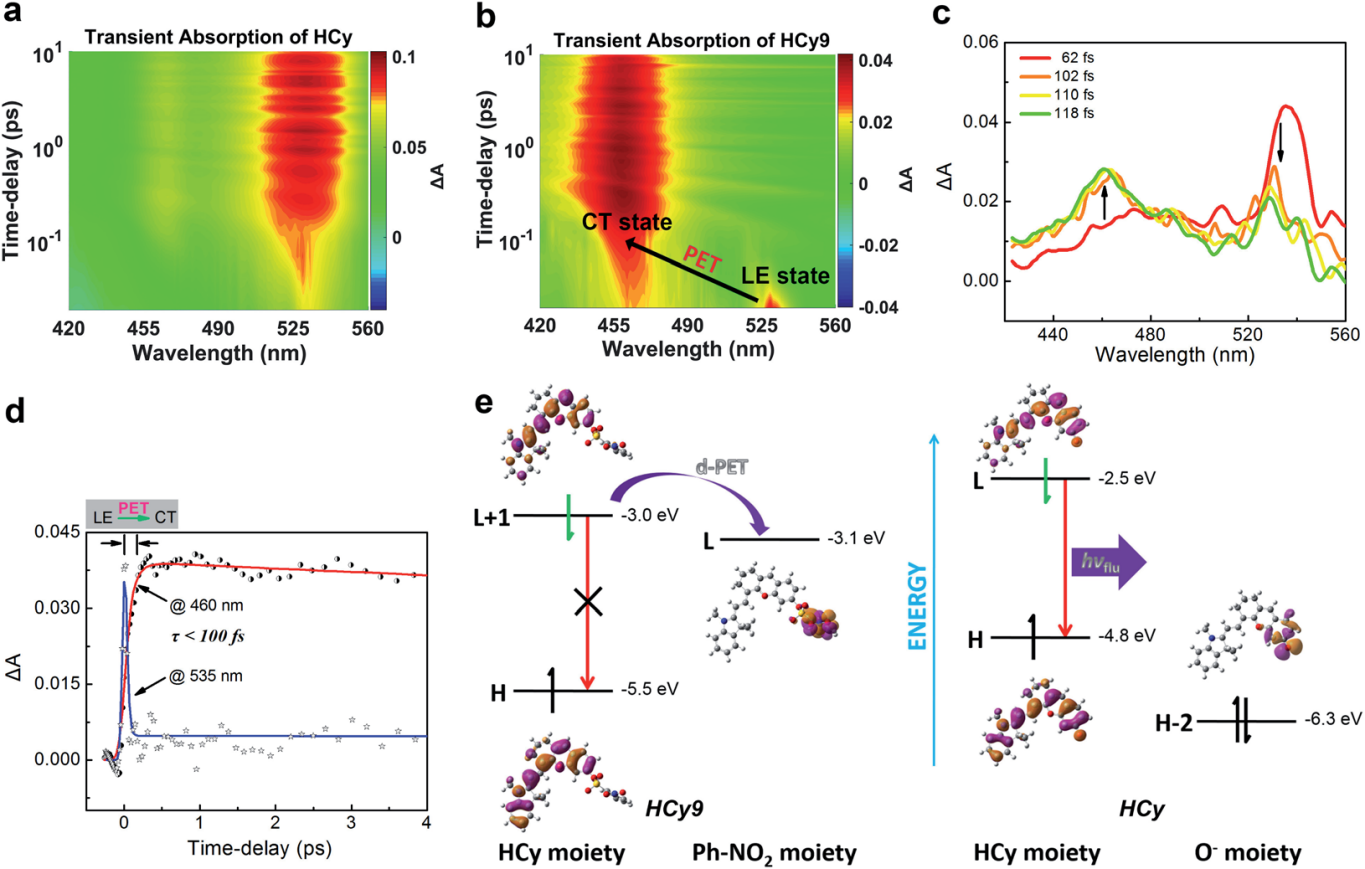
(a and b) Pseudocolor femtosecond transient absorption (TA) spectra of (a) **HCy** and (b) **HCy9** in DMSO. (c) Femtosecond TA spectra recorded at different time-delays (62–118 fs) after femtosecond laser excitation (630 nm). (d) Kinetic traces at different wavelengths following 630 nm laser pulse excitation and the respective fit with two (@535 nm) or three (@460 nm) exponential functions. (e) TD-DFT calculations on the electronic transitions of **HCy9** and **HCy** in DMSO at the B3LYP/aug-cc-pVDZ level.

It should be noted that relative to non-NIR probes, NIR ones are generally more difficult to regulate *via* photoinduced electron transfer (PET) due to the relatively smaller excitation energy, which results in the failure to produce charge separation.^31–36^ In other words, NIR fluorophores usually have a lower LUMO and/or higher HOMO, and this will reduce the driving force (Δ*G*_PET_) of the PET process regardless of the donor-type (d-PET) or acceptor-type (a-PET) (Scheme S2†). This can be reflected by the stronger autofluorescence of **HCy9** (refer to the black line in Fig. S3i† when *t* = 0) relative to **NI9**.^13^ Additionally, as an isomer of **HCy10**, **HCy9** was found to exhibit obviously stronger autofluorescence than the former (*cf.* the black lines in Fig. S3i and j† when *t* = 0). Consequently, we examined if the quenching mechanism of **HCy9** was still PET and explored the specific inherent reason for the above phenomena. For this purpose, with **HCy** as the control molecule, measurements of the femtosecond transient absorption (TA) spectra and calculations of electronic transitions based on time-dependent density functional theory (TD-DFT) were performed. Upon excitation using a laser at 630 nm, a distinct transient absorption band of **HCy** centered at *ca.* 535 nm appeared instantly (<100 fs; Fig. 3a), which should be attributed to the locally excited (LE) state. This state could last for more than 10 ps (Fig. 3a). As for **HCy9**, the same excited-state absorption (ESA) signal centered at *ca.* 535 nm was observed in the first 60 fs upon excitation at 630 nm (Fig. 3b, d and S10†). However, in the next 60 fs, the band at 535 nm disappeared gradually, accompanied by the formation of a brand-new one centered at *ca.* 460 nm (Fig. 3b–d), indicating the generation of a new state. The time scales obtained by fitting the data at 535 and 460 nm, respectively, are comparable (<100 fs), suggesting that the new state is derived from the LE state. In TD-DFT calculations, regarding **HCy9**, the oscillator strength *f* of the transition S_0_ → S_1_ is quite small (0.007) (Table S8†), indicating that the S_1_ state can be hardly formed directly from the ground state S_0_ upon excitation. Instead, the S_1_ state may be derived from a higher state S_2_ (Kasha's rule), which can be populated directly from the S_0_ state upon excitation since the *f* of the corresponding transition is considerable (1.274). Noticeably, the S_0_ → S_2_ transition corresponds to HOMO → LUMO+1 (Table S8†), and both molecular orbitals locate on the **HCy** moiety (Fig. 3e, left), demonstrating that the S_2_ state is the LE state. Meanwhile, the S_0_ → S_1_ transition corresponds to HOMO → LUMO (Table S8†), and the LUMO locates on the receptor unit moiety, demonstrating that the S_1_ state is the charge-transfer (CT) state. Hence, the transformation from S_2_ to S_1_ is essentially the PET process, and the new state generated in the second 60 fs corresponds to the CT state (Fig. 3b–d). As a comparison, the lowest excited state S_1_ of **HCy** can be populated directly from the S_0_ state (*f* = 1.124) (Table S8†), and the corresponding HOMO and LUMO both locate in the same region (Fig. 3e, right), indicating that the S_1_ state is the LE state, from which the fluorescence can be emitted unimpededly. Incidentally, to our knowledge, this is the first time that the real PET process of a NIR fluorescent probe has been captured experimentally. In brief, the quenching mechanism of **HCy9** is still PET, and the lower quenching efficiency relative to that of **NI9** is presumably due to the reduced driving force (0.1 eV in Fig. 3e*vs.* 0.4 eV for **NI9**)^13^ arising from the lower LUMO of the NIR fluorophore (−3.0 eV *vs.* −2.7 eV).^13^ And the higher quenching efficiency of **HCy10** relative to **HCy9** is probably related to the lower LUMO of the quencher moiety (*i.e.* the receptor unit) (−3.1 eV in Fig. 3e*vs.* −3.3 eV for **HCy10** in Fig. S11 and Table S8†), recovering the driving force from 0.1 eV in **HCy9** to 0.3 eV in **HCy10**, which is close to the 0.4 eV in **NI9**. This is in line with **HCy10**'s higher *ω*_*k*_ (Table S1†), which means that the properties (*e.g.* reactivity) of the receptor unit can also affect the probe's fluorescence behavior.

### Assessment of applications of **HCy**-based probes in cells, tissues and tumors *in vivo*

**Fig. 4:**
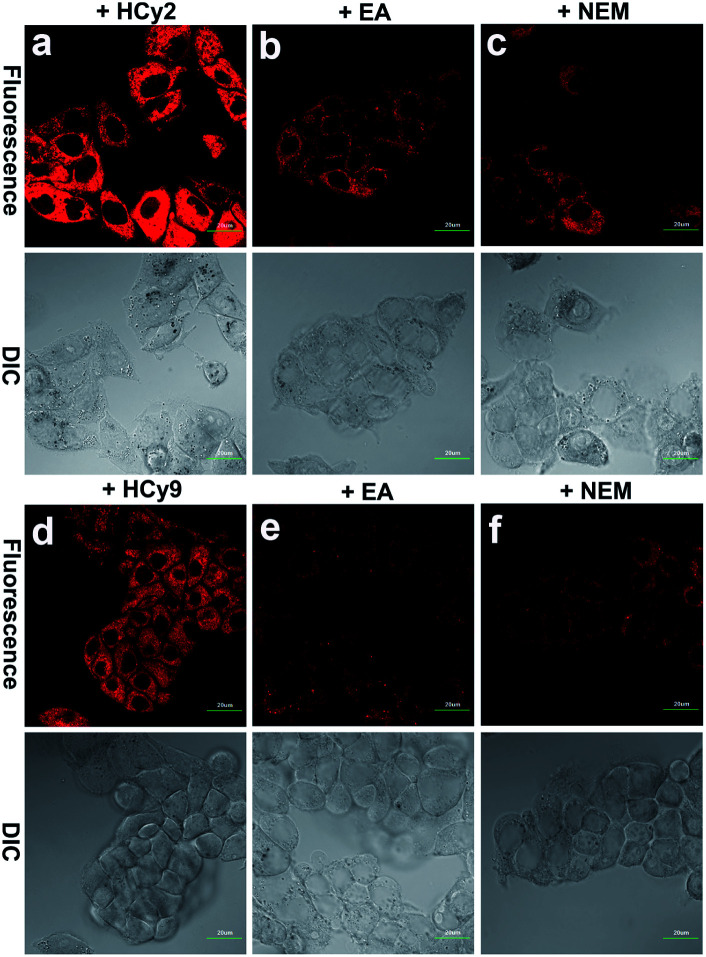
Fluorescence images of HepG2 cells incubated with 10 μM (a) **HCy2** or (d) **HCy9**, pretreated with 100 μM EA and then incubated with 10 μM (b) **HCy2** or (e) **HCy9** and pretreated with 50 μM NEM and then incubated with 10 μM (c) **HCy2** or (f) **HCy9** obtained with a 100× objective. *λ*_ex_ = 633 nm. *λ*_em_ = 680–780 nm. Scale bar = 20 μm. Representative images from repeated experiments are shown.

Since HepG2 cells are rich in GSTA1-1, as demonstrated by western blotting analysis in the literature,^13^ 10 μM **HCy2** or **HCy9** in HEPES buffer (20 mM, 0.25% DMSO, 5% glucose, pH 7.4) was used to incubate HepG2 cells, after which fluorescence images were captured. As shown in Fig. S12, S13 and Video in the ESI,† in both cases, the cells could be lit up gradually in 30 min. To examine if the fluorescence resulted from GST activity, HepG2 cells were pretreated with the GST inhibitor EA or the GSH-depleting agent *N*-ethylmaleimide (NEM), respectively. For either **HCy2** or **HCy9**, both EA and NEM could induce an obvious fluorescence loss (Fig. 4), verifying their capability for detecting GST activity in living cells. By contrast, for **HCy1** and **HCy3–5**, hardly any fluorescence decrease was observed when HepG2 cells were preincubated with EA (Fig. S14†), ruling out their qualification as probes for GST activity, which is in line with *in vitro* results (Fig. S6b†). Subsequently, a colocalization analysis demonstrated that the produced fluorescent dye **HCy** tended to accumulate in the lysosomes probably due to its charge characteristics (Fig. S15–18†),^37^ in agreement with the results in the literature.^37–39^

Although **HCy2** and **HCy9** were both amenable to cellular imaging for GST activity, they were found to differ obviously in sensitivity during the imaging process. As investigated by both fluorescence imaging and flow cytometry, **HCy2** was more sensitive to GST than **HCy9** under the same conditions (Fig. S19a, b and d†), in agreement with their *k*_cat_ values (Table S5†). This can be unquestionably attributed to their difference in *ω*_*k*_ (Table S1†). Nonetheless, as discussed previously, in addition to *ω*_*k*_, the *o*-NO_2_ group is essential for GST catalysis as well, which was also confirmed by comparing the performances of **HCy9** and **HCy10** in cell samples (Fig. S19b–d†). It is worthwhile to mention that the *ω*_*k*_ of **HCy10** is larger than that of **HCy9** (Table S1;† recall that the *k*_nonc_ of **HCy10** can be determined while the data for **HCy9** were undetectable; see also Fig. S3i and j†). And this result demonstrates indirectly that the fluorescence in **HCy9**-incubated HepG2 cells was not induced by GSH alone but by GST activity.

**Fig. 5:**
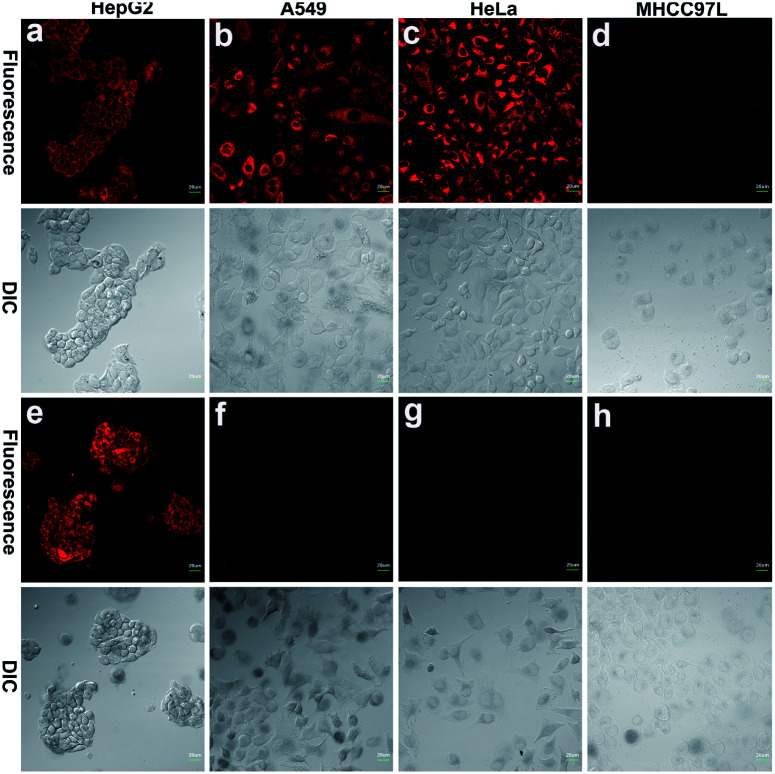
Fluorescence images of various cell lines incubated with 10 μM (a–d) **HCy2** or (e–h) **HCy9** with a 40× objective. *λ*_ex_ = 633 nm. *λ*_em_ = 680–780 nm. Scale bar = 20 μm. Representative images from repeated experiments are shown.

**Fig. 6:**
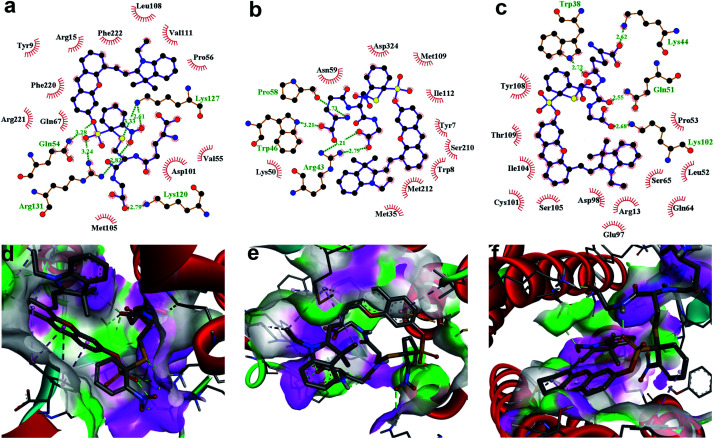
Docking simulations of the **GS-HCy9** σ complex in (a and d) GSTA1-1, (b and e) GSTM1-1 and (c and f) GSTP1-1, respectively. (a–c) 2D Ligplot+ analysis of active-site interactions. C, N, O and S atoms are shown in black, blue, red and yellow, respectively. H-Bonds and the hydrophobic interactions between the **GS-HCy9** σ complex and amino acid residues of GST are indicated with green dotted lines and red curves, respectively. (d–f) 3D visualization of active-site interactions by DS Visualizer analysis. H-Bond surfaces of GST relative to the ligand are displayed with the donor and acceptor colored in pink and green, respectively. H-Bonds are marked with green dotted lines.

To further demonstrate that both **HCy9** and **HCy2** are GST-activity-specific, another three cell lines besides HepG2 were employed, namely A549, HeLa and MHCC97L, according to the western blotting results in the literature.^13^ Therein, A549 cells are rich in GSTP1-1 with a tiny amount of GSTA1-1 contained, and HeLa cells are rich in GSTM1-1 and GSTP1-1. By contrast, MHCC97L cells contain no GSTA1-1, GSTM1-1 or GSTP1-1. As shown in Fig. 5d and h, MHCC97L cells incubated with **HCy2** or **HCy9** exhibited little fluorescence, indicating the probes' specificity for GSTs. Interestingly, both A549 and HeLa cells incubated with **HCy2** displayed bright fluorescence while rather dim fluorescence was observed when **HCy9** was used for incubation (Fig. 5b, c, f and g). Taking the specific GST isoenzymes contained in HepG2, A549 or HeLa into account, these results are consistent with the *k*_cat_ values of **HCy2** and **HCy9** towards different GST isoenzymes (Tables S5–S7†). In other words, **HCy2** has broad isoenzyme selectivity whereas **HCy9** shows specificity for GSTA1-1. To address this issue, docking simulations of the σ complex^30,40–42^ (*i.e.* Meisenheimer complex) for **HCy9** or **HCy2** into these three GST isoenzymes were implemented. For the **GS-HCy9** σ complex, in regard to GSTA1-1, abundant H-bonds are formed between residues in the active-site and the receptor unit moiety of **HCy9** (Fig. 6a). And in particular, two H-bonds are centered on the *o*-NO_2_ group, which is highly favorable for lowering the activation energy barrier by stabilizing the σ complex and thus facilitates GST catalysis. In contrast, as regards GSTM1-1 and GSTP1-1, all the H-bonds are centered on the GSH moiety (Fig. 6b and c). These results were based on well-known Ligplot+ analysis.^43^ And another notable piece of analysis software, DS Visualizer, gave a similar result (Fig. S20†). In addition, as can also be reflected in 3D visualization, the colored section of H-bond surfaces is focused on the receptor unit moiety for GSTA1-1 whereas for GSTM1-1 and GSTP1-1, they are scarcely located on this moiety (Fig. 6d–f). As for the **GS-HCy2** σ complex, there exist H-bonds between the receptor unit moiety and residues in the active-sites of all three isoenzymes (Fig. S21†). Although relative to the number of “effective” H-bonds for the **GS-HCy9** σ complex in GSTA1-1, the ones for the **GS-HCy2** σ complex in all three isoenzymes appear fewer; this makes sense since the activation energy barrier for **HCy2** is lower, and a smaller number of “effective” H-bonds could suffice for the catalysis process. The lower activation energy barrier arises from the higher *ω*_*k*_. Therefore, to some extent we can say that the difference in reactivities of **HCy2** and **HCy9** leads to their different behaviors in isoenzyme selectivity, in agreement with the conclusion in previous literature.^7^

**Fig. 7:**
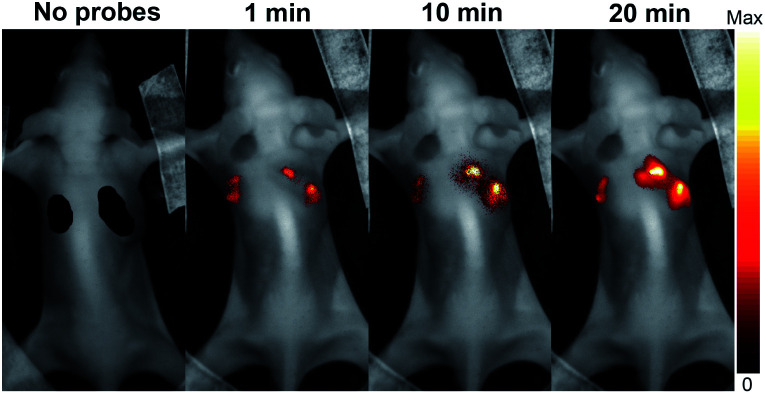
*In vivo* serial whole-body imaging of GST expression in a nude mouse bearing HepG2 tumors. 100 μL of 20 μM **HCy9** or **HCy2** in HEPES buffer (20 mM, 0.5% DMSO, pH 7.4) was injected into the left or the right tumor, respectively. *λ*_ex_ = 661 nm. *λ*_em_ = 700–800 nm.

It is often desirable that probes could afford deeper imaging in practical applications. Herein, as a NIR fluorescent probe, **HCy2** proved to be competent to detect GSTs at a depth of 50 μm in liver and lung tissues (Fig. S22†), which, respectively, contain abundant GSTA or GSTP isoenzymes.^44,45^ Actually, the NIR characteristics make **HCy2** behave well even at a depth deeper than 50 μm so that fluorescence images at various depths could afford a 3D reconstruction of the tissue sections (Fig. S23 and S24†). The outstanding performances of **HCy2** inspired us to inspect if these NIR probes could be applied for *in vivo* fluorescence imaging. Hence, **HCy2** and **HCy9** were then subjected to monitoring of GST activity in HepG2 tumor xenografts embedded in nude mice. As shown in Fig. S25,† while the left control tumor displayed faint fluorescence, the right tumor turned brighter gradually in 20 min after injection with **HCy9**, demonstrating that even this less sensitive probe relative to **HCy2** was able to realize GST detection *in vivo* in real time. And undoubtedly, with respect to **HCy9**, herein **HCy2** is also more sensitive to GST (Fig. 7), similar to the results observed in cells (Fig. S19†). To sum up, the NIR properties endow the probes screened in this work with competence for deep imaging.

## Conclusions

In summary, high-performance NIR fluorescent probes for GSTs have been developed in this work, and we have found that the reactivity (both nonenzymatic and enzymatic) of a probe is related to both the receptor unit and the fluorophore. Specifically, the higher the electron-deficiency of the fluorophore is, the more prone to reacting with GSH the probe is, which makes **HCy**-based probes universally more sensitive to GSH and GST activity than **NI**-based ones. On the other hand, the difference in the receptor unit between **HCy2** and **HCy9** results in their different reactivities and thus distinct isoenzyme selectivities. By virtue of the high brightness and NIR character of **HCy**, **HCy2** and **HCy9** have been proven to be competent to detect GST in a variety of biological samples including cells, tissues and tumors *in vivo*. Additionally, although NIR fluorophores are generally difficult to quench *via* PET, the quenching mechanism of **HCy**-based probes herein has been demonstrated to be PET experimentally and theoretically. Besides, the fluorescence behavior of a probe has also been found to be connected with both the fluorophore and the receptor unit. That is to say, although the properties of a probe can be artificially divided into two parts according to eqn (1), namely the photoluminescence mechanism and the recognition or reaction mechanism, the probe should be treated as an entirety. These results will provide a distinct framework for designing more practical NIR fluorescent probes for GSTs in the future.

## Conflicts of interest

There are no conflicts to declare.

## Supplementary Material

SC-011-D0SC04411C-s001

SC-011-D0SC04411C-s002
