# Supplementary material for: A fluorophore's electron-deficiency does matter in designing high-performance near-infrared fluorescent probes
Source: Chem Sci. 2020 Sep 21;11(41):11205–13. doi: 10.1039/d0sc04411c (PMC8162715; doi:10.1039/d0sc04411c)
Supplement: SC-011-D0SC04411C-s001 [file SC-011-D0SC04411C-s001.pdf]

Electronic Supplementary Material (ESI) for Chemical Science.
This journal is © The Royal Society of Chemistry 2020

Supporting Information for

Fluorophore's Electron-Deficiency Does Matter in Designing High-Performance Near-Infrared Fluorescent Probes

Xue-Xiang Zhang[†,§], Huan Qi[‡,§], Ya-Lan Liu[†], Song-Qiu Yang[†], Peng Li[//], Yan Qiao[⊥], Pei-Yu Zhang[¶], Shu-Hao Wen[¶], Hai-long Piao[*,‡] and Ke-Li Han[*,†,//]

[†]State Key Laboratory of Molecular Reaction Dynamics, Dalian Institute of Chemical Physics, Chinese Academy of Sciences, Dalian 116023, P. R. China

[‡]CAS Key Laboratory of Separation Science for Analytical Chemistry, Dalian Institute of Chemical Physics, Chinese Academy of Sciences, Dalian 116023, P. R. China

[//]Institute of Molecular Sciences and Engineering, Shandong University, Qingdao 266237, P. R. China

[⊥]Department of Pathophysiology, Basic Medical College of Zhengzhou University, Zhengzhou 450001, P. R. China

[¶]Shenzhen Jingtai Technology Co., Ltd., Floor 4, No. 9, Hualian Industrial Zone, Dalang Street, Longhua District, Shenzhen, P. R. China

[§]These authors contributed equally.

Table of Contents

Materials and Instruments 3
Experimental Procedures and Calculation Methods 3
Synthesis and Characterizations 5
Deduction I 8
Deduction II 9
Deduction III 9
Supplementary Figures, Schemes and Tables 10
NMR Spectra Data 25
Cartesian coordinates (angstrom) for the optimized structures of probe models used for ω calculations 37
Cartesian coordinates (angstrom) for the optimized structures of probe models used for P_k^+ calculations 46
Cartesian coordinates (angstrom) for the optimized structures of DNs-series probes used for P_k^+ and ω calculations 54
Cartesian coordinates (angstrom) for the optimized structures of HCy9, HCy and HCy10 used for TD-DFT calculations 56
References 58

Materials and Instruments

Unless otherwise specified, all reagents of analytical reagent grade were acquired from commercial source and were used without further purification. *In vitro* tests, kinetic measurements and assay in cell lysate samples employed 96-well black flat bottom polystyrene NBS™ microplate (Corning® Product #3650, USA) with a microplate reader Varioskan Flash (Thermo Fisher Scientific, USA). GSTs from equine liver (Sigma-Aldrich #G6511, USA) were used for *in vitro* tests. GSTA1-1, GSTM1-1 and GSTP1-1 (Abcam #ab167981, #ab168035 and #ab167990, respectively, UK) were used for enzymatic kinetic study. A 4 mM stock solution (dissolved in DMSO) were freshly prepared for all **HCy**-series probes, followed by dilution with HEPES buffer before use. ^{1}H, ^{13}C and ^{19}F NMR spectra were taken on a nuclear magnetic resonance spectrometer (Bruker, Germany) at room temperature. High resolution mass spectra (HDMS) were recorded on 6540 UHD Accurate-Mass Q-TOF LC/MS instrument (Agilent, USA). The *in vivo* fluorescence images of mice were obtained with the Maestro EX in vivo Imaging System (CRi, USA).

Experimental Procedures and Calculation Methods

Kinetic measurements.

Nonenzymatic and enzymatic kinetic study were performed by monitoring changes in fluorescence intensity at 700 nm upon excitation at 650 nm with a 96-well black flat bottom microplate. All measurements were accomplished in HEPES buffer (20 mM, < 1.25% DMSO, pH 7.4) at 37 °C in duplicate. The final volume of the mixed solution was 200 μL. To determine the concentration of newly-produced **HCy**, the fluorescence emission at 700 nm was measured upon excitation at 650 nm.

For nonenzymatic kinetic study, the concentration of probes was ranging from 0 to 6.25 μM (for **HCy10**, the concentration was from 0 to 25 μM), and the second-order rate constant k_{nonc} was determined by direct fits of the data to the equation (1) using a linear regression[1], in which the subscript '0' represents the initial state of the reaction.

$$v_{0,nonc} = k_{nonc}[\mathrm{GSH}]_0[\mathrm{Probe}]_0 \quad (1)$$

For enzymatic kinetic study, the final enzyme concentration was 1.984, 0.992 and 2.500 μg/mL for GSTA1-1, GSTM1-1 and GSTP1-1, respectively. Briefly, a certain probe of a series of concentrations (from 0.39 to 50 μM) was added to the enzyme solution premixed (ten minutes earlier) with GSH (2 mM, final concentration 1 mM) to initiate the reaction. With the obvious nonenzymatic reaction noise being subtracted from the enzymatic reaction signal, the initial reaction rates were determined by the data within a linear response range. Kinetic parameters (K_m, k_{cat} and k_{cat}/K_m) were calculated by direct fits of the data to the Michaelis-Menten Equation using a nonlinear regression.

UPLC-MS analysis.

UPLC-MS analysis was performed on a ZORBAX Eclipse Plus C18 column (2.1 × 50 mm, 1.8-Micron, Agilent, USA) using 6540 UHD Accurate-Mass Q-TOF LC/MS instrument (Agilent, USA). LC signal was detected based on absorbance at 600 or 400 nm with the conditions: flow rate being 200 μL/min; sample amount being 0.5 μL; eluent ingredient being 5% A to 95% A over the initial 1 min and then maintaining the eluent ingredient for 15 min (solvent A and B are water with 0.1% formic acid and acetonitrile, respectively). The final concentrations of **HCy2**, **HCy** and GSH were 20 μM, 20 μM and 1 mM, respectively.

Cell culture

Human hepatocellular carcinoma cells HepG2 and MHCC97L were obtained from the American Type Culture Collection (ATCC, Manassas, VA). Human breast cancer cells A549 and human cervical cancer cells Hela were purchased from Cell Bank of Shanghai Institute of Cell Biology, Chinese Academy of Sciences. HepG2 cells were cultured in Dulbecco's Modified Eagle's Medium (DMEM, Thermo Fisher Scientific) supplemented with 10% fetal bovine serum (FBS, Thermo Fisher Scientific) and 1% penicillin-streptomycin (P/S, Thermo Fisher Scientific), and others were cultured in RPMI 1640 medium (Thermo Fisher Scientific) with 10% FBS and 1% P/S. All cells were maintained in a humidified atmosphere containing 5% CO_2 at 37℃ and were seeded in glass bottom dish (φ20 mm, NEST) with a density of 1×10^4 cells per dish for imaging.

Fluorescence imaging in Cells and Tissues

Fluorescence microscopic images in cells and tissue sections were captured by a FV1000 confocal laser scanning microscope (Olympus, Japan). Direct imaging was implemented without washing.

For imaging related to EA and NEM, operation conditions were as follows: (1) To eliminate any interferences induced by long-time incubation in HEPES buffer, HepG2 cells were pretreated with HEPES buffer (20 mM, 5% glucose, pH 7.4) for 40 min and then incubated with 10 μM **HCy**-based probes in HEPES buffer for 30 min before subjected to imaging; (2) HepG2 cells were pretreated with 100 μM EA in HEPES buffer for 30 min and then incubated with 10 μM **HCy**-based probes in HEPES buffer in the presence of EA for another 30 min before subjected to imaging; (3) HepG2 cells were pretreated with 50 μM NEM in HEPES buffer for 40 min and then incubated with 10 μM **HCy**-based probes in HEPES buffer in the absence of NEM for another 30 min before subjected to imaging.

For comparison of fluorescence intensities of various cell lines incubated with **HCy2** or **HCy9**, the incubation time was 30 min or 1.5 h for **HCy2** or **HCy9**, respectively.

For fluorescence imaging in tissues, mice liver or lung tissue sections were incubated with 20 μM **HCy2** in HEPES buffer (20 mM, 0.5% DMSO, 5% glucose, pH 7.4) for 1 h before imaging.

Flow cytometry.

FACSCanto™ II (BD, USA) was used for the flow cytometric analysis upon excitation at 633 nm, with fluorescence signal collected at 680−780 nm (using APC-Cy7 optical filter). HepG2 cells were incubated with 10 μM **HCy2**, **HCy9** or **HCy10** in HEPES buffer (20 mM, 0.25% DMSO, 5% glucose, pH 7.4) for 30 min prior to FACS.

Establishment of tumor model in mice

All animal experiment procedures were performed according to the protocols approved by the Animal Ethics and Use Committee of Dalian Medical University. Female BALB/c-nu mice with 5 weeks were obtained from the animal center of Dalian Medical University and were housed under standard conditions with 12 h light/dark cycle. The tumors were grafted by subcutaneous injection of 5×10^6 HepG2 cells in 200 μL of PBS-matrigel (1:1) mixture into the front flanks of each mouse. Subsequently, when tumor size reached about 300 mm^3, *in vivo* fluorescence imaging was carried out.

In vivo whole-body imaging

The mice were anesthetized by 4% chloral hydrate and intratumorally injected with 100 μL 20 μM **HCy9** ot **HCy2** in HEPES buffer (20 mM, 0.5% DMSO, pH 7.4). The control tumor was intratumorally injected with HEPES buffer (20 mM, 0.5% DMSO, pH 7.4) alone. Serial fluorescence images were captured after injection. Fluorescence images were acquired using the following parameters: Excitation Wavelength: 661 nm; Emission Filter: 700 nm long-pass filter; Acquisition Settings: 700–800 nm in 10 nm steps; Exposure times were 400 ms.

Femetosecond TA spectroscopy

TA experiment was conducted with a home-made femtosecond pump-probe setup.[2] Briefly, laser pulses (800 nm, 50 fs pulse length, 1 kHz repetition rate) were generated by a Ti: sapphire femtosecond laser source (Spitfire, Spectra-Physics). An optical parametric amplifier was used to change the laser wavelength. The white light-continuum generation from a thin CaF_2 plate was used for the probe. By placing a Berek compensator in the pump beam, the mutual polarization between pump and probe beams was set to the magic angle (54.7°). The wavelength and the power of the pump beam were 630 nm and 100 μW, respectively, and the spot size was 0.3 mm in diameter. A solution of *ca.* 1 mM **HCy9** in DMSO (Spectrum Pure, >99.9% GC) was subjected to the measurement. The kinetic of the repeated scans remained the same, indicating no sign of degradation.

Calculations of ω, spin density distribution, P_k^+ and ω_k

The global elctrophilicity ω values were obtained based on the calculations of vertical ionization potential (I) and vertical electron affinity (A) with ORCA 4.0.1 program at the PWPB95/def2-QZVPP level[3] according to the formulas for the valance state parabola model in the literature[4], utilizing RIJCOSX acceleration with auxiliary basis sets def2/J[5] and def2-QZVPP/C[6] and involving the atom-pairwise dispersion correction with the Becke-Johnson damping scheme (D3BJ)[7]. The integral grid was set to 4, and the convergence limit was set as ‘tightSCF’. In light of the analysis in the literature[4] and the probes’ applications in biological systems, all these calculations were implemented in gas phase. The global elctrophilicity ω in the valence state parabola model was calculated according to the Equations (2) – (4)[4], in which μ means the electronic chemical potential and η represents the chemical hardness:

$$\mu = -(I + A)/2 \tag{2}$$

$$\eta = (I - A)/2 \tag{3}$$

$$\omega = \mu^2/2\eta = (I + A)^2/4(I - A) \tag{4}$$

The calculation method for P_k^+. Specifically, geometry optimization and vibrational frequency analysis were carried out at the B3LYP/6-31G(d) level[8] of DFT theory. The P_k^+ values were obtained based on natural population analysis (NPA) charge by single-point energy calculations over the optimized neutral geometries (since the positive charge in π-conjugated organic dye like cyanine or **HCy** always brings about problems in accuracy,[9] and considering the indolium moiety bearing the positive charge not being a reactive site, a simplified model for **HCy**-based probes with the deletion of methyl on the nitrogen of indolium ring was used) using the unrestricted UB3LYP formalism for the radical anion of compounds at the same level. And the results of these calculations were analysed by Multiwfn software[10] to give spin density distributions. Above calculations were conducted with Gaussian 09 suite of programs[11].

The ω_k values were calculated according to the Equations (5).

$$\omega_k = \omega \cdot P_k^+ \tag{5}$$

Docking Simulations

Homology modeling of GSTM1-1 protein structure. The starting structure was homologously modeled on the basis of single template using Modeller 9.20 software. The template (PDB code: 6GSV) was obtained from the RCSB PDB. The alignment result was then used as the input for Modeller, and a total of ten models were generated and evaluated by the default DOPE potential in Modeller. The best GSTM1-1 model was chosen to be applied for docking simulations.

The crystallographic structures of GSTA1-1 (PDB code: 1K3L) and GSTP1-1 (PDB code: 18GS) were retrieved from the RCSB PDB. Afer adding the explicit hydrogen atoms, deleting all water molecules and removing the ligands, we processed the protein structure using AutoDock Tools with Grid box size of 40/54/45 around center 80.5/26/18.5, 45/42/45 around center 20.5/17.5/31 and 54/45/50 around center 8/8/26 for GSTA1-1, GSTM1-1 and GSTP1-1, respectively. Geometrically optimized **HCy9-GS** and **HCy2-GS** σ complexes were prepared for docking by using AutoDock Tools to assign AD4 atom types, calculate Gasteiger charges and set all rotatable bonds as active torsions except the one in rigid fluorophore moiety. The complexes were docked into the protein using AutoDock 4. The exhaustiveness parameter was set as default. The maximum number of evals was set as long (25,000,000).

TD-DFT calculations
The calculation method was same with the one in the case of **NI**-based probes.[2] Briefly, in order to mimic the real process of photoexcitation in femetosecond TA spectroscopy, the solvent effect in DMSO solution was employed in the SCRF calculations by using the SMD solvation model[12]. Geometrical optimizations of **HCy9**, **HCy** and **HCy10** in the ground state and vibrational frequency analysis were carried out at the B3LYP/6-31G(d) level[8] of DFT method. The electron transitions of **HCy9**, **HCy** and **HCy10** were studied at the B3LYP/aug-cc-pVDZ level[13-15] based on TD-DFT method conducted with Gaussian 16 suite of programs[16].

Synthesis and Characterizations

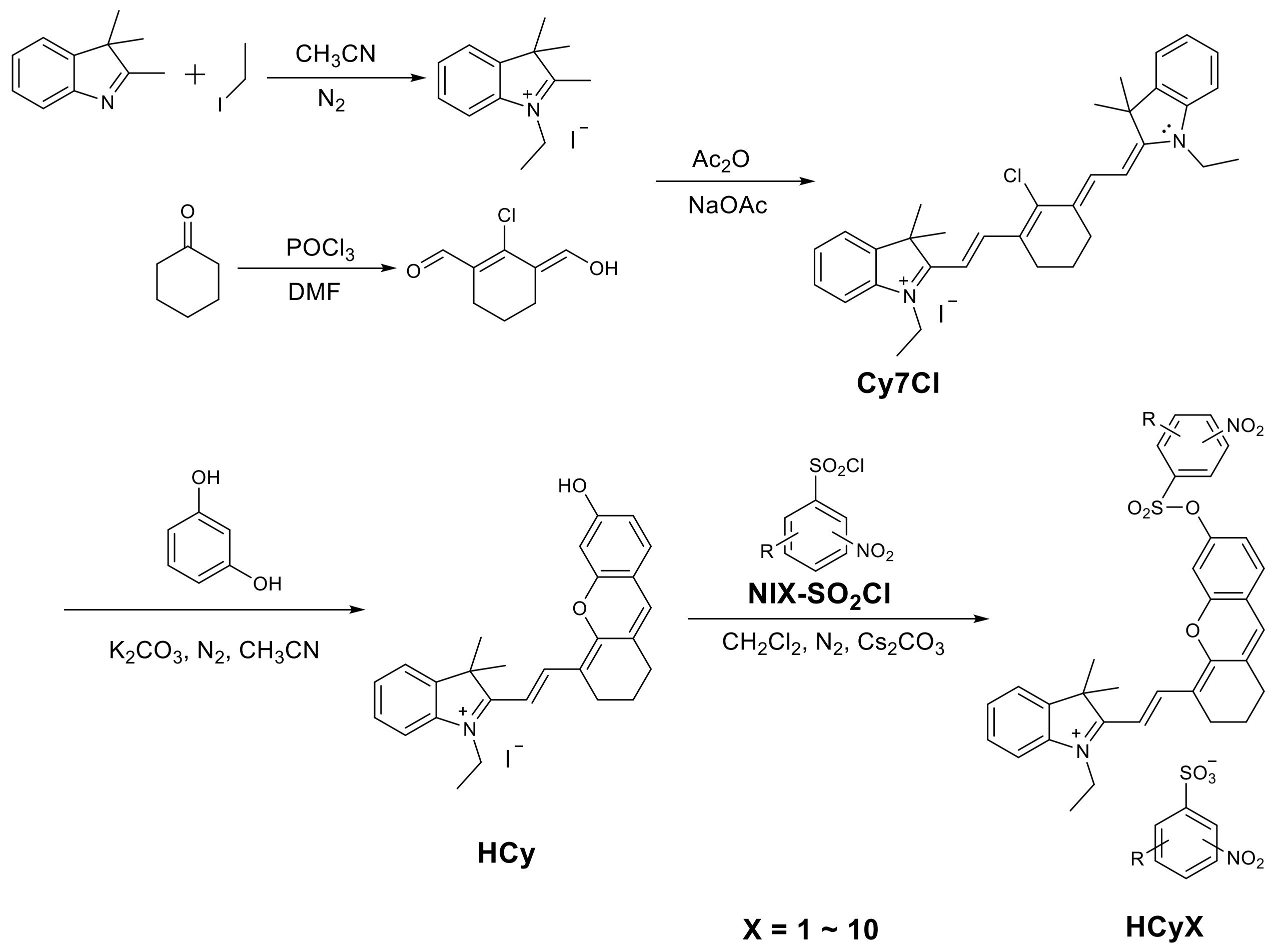

Cy7Cl
To a stirred solution of 2,3,3-trimethyl-3*H*-indole (32 g, 200 mmol) and acetonitrile (500 mL) was added iodoethane (80 g, 500 mmol) under N_2 atmosphere. Then the mixture was heated at 80 °C for 48 h, after which the solvent was removed *in vacuo*. The residue was washed with diethyl ether and dried to give 1-ethyl-2,3,3-trimethyl-3*H*-indolium iodide as a purple solid (62 g, yield 98%). HRMS (ESI$^+$): [M]$^+$, calcd. for $C_{13}H_{18}N^+$, 188.1434; found 188.1438.
To a stirred solution of DMF (40mL, 540 mmol) and dichloromethane (40 mL) was added phosphoryl chloride (37 mL, 230 mmol) dropwise at 0 °C. And 30 min later, cyclohexanone (10 g, 100 mmol) was added dropwise at 0 °C. The mixture was refluxed at 60 °C for 6 h, and then was cooled with ice bath, after which the solution was poured onto the crushed ice for overnight. Afterwards, the filter residue was washed with cool water after filtration, and then was dried to give 2-chloro-3-(hydroxymethylene)-1-cyclohexene-1-carboxaldehyde as a yellow solid (15.95 g, yield 92%). HRMS (ESI$^-$): [M-H]$^-$, calcd. for $C_8H_8ClO_2^-$, 171.0218; found 171.0215.
In a three-necked, round-bottomed flask provided with a magnetic stirrer was placed 1-ethyl-2,3,3-trimethyl-3*H*-indolium iodide (12.6 g, 40 mmol), 2-chloro-3-(hydroxymethylene)-1-cyclohexene-1-carboxaldehyde (3.44 g, 20 mmol), acetic anhydride (70 mL) and sodium acetate (3.3 g, 40 mmol). The solution was refluxed foe 3 h at 70 °C, after which the mixture was poured into a concentrated solution of potassium iodide. The solution was stirred at 70 °C for 1 h, and then the filter residue was washed with ethyl ether after filtration and dried to give **Cy7Cl** as a yellow green solid (12.06 g, yield 94%). ^{1}H NMR (400 MHz, DMSO-d_6) δ 8.27 (d, J = 14.1 Hz, 2H), 7.67 – 7.63 (m, 2H), 7.49 – 7.42 (m, 4H), 7.33 – 7.27 (m, 2H), 6.34 (d, J = 14.2 Hz, 2H), 4.27 (q, J = 7.1 Hz, 4H), 2.73 (t, J = 6.1 Hz, 4H), 1.90 – 1.82 (m, 2H), 1.68 (s, 12H), 1.32 (t, J = 7.1 Hz, 6H). ^{13}C NMR (101 MHz, DMSO) δ 172.25, 148.47, 143.56, 142.11, 141.66, 129.14, 126.57, 125.65, 123.05, 111.79, 101.77, 49.48, 39.50, 27.87, 26.37, 20.86, 12.72. HRMS (ESI$^+$): [M]$^+$, calcd. for $C_{34}H_{40}ClN_2^+$, 511.2875; found 511.2884.

HCy

This synthesis procedure was according to the literature.[17] To a stirred solution of resorcinol (4.4 g, 40 mmol) in acetonitrile (100 mL) was added K_2CO_3 (5.53 g, 40 mmol), and then keep the reaction on at RT for *ca.* 1 h until the color of the solution turned yellow. Afterwards, the solution of **Cy7Cl** (12.8 g, 20 mmol) in acetonitrile (215 mL) was added to the abovementioned solution and the stirred mixture was heated at 50 °C for 10 h, after which the solvent was removed *in vacuo*. The crude product was chromatographed on silica gel (MeOH/CH_2Cl_2 = 5:500→40:500) to give **HCy** as a dark blue solid (3.42 g, yield 33%). ^{1}H NMR (400 MHz, Chloroform-*d*) δ 8.14 (d, *J* = 13.5 Hz, 1H), 7.36 – 7.28 (m, 3H), 7.23 (d, *J* = 9.0 Hz, 1H), 7.08 (t, *J* = 7.4 Hz, 1H), 6.88 (d, *J* = 8.0 Hz, 1H), 6.81 (dd, *J* = 9.0, 2.0 Hz, 1H), 6.63 (t, *J* = 1.3 Hz, 1H), 5.69 (d, *J* = 13.5 Hz, 1H), 3.91 (q, *J* = 7.2 Hz, 2H), 2.69 (t, *J* = 6.0 Hz, 2H), 2.63 (t, *J* = 6.1 Hz, 2H), 1.90 (p, *J* = 6.2 Hz, 2H), 1.67 (s, 6H), 1.37 (t, *J* = 7.2 Hz, 3H). ^{13}C NMR (101 MHz, $CDCl_3$) δ 165.92, 160.47, 158.72, 142.79, 140.01, 139.89, 133.86, 129.72, 128.25, 126.35, 122.67, 122.15, 117.27, 116.21, 115.83, 108.16, 103.50, 94.57, 77.37, 77.05, 76.73, 47.77, 38.25, 28.57, 28.06, 24.52, 21.26, 11.66. HRMS (ESI$^+$): [M]$^+$, calcd. for $C_{27}H_{28}NO_2^+$, 398.2115; found 398.2126.

HCyX

To synthesize **HCyX**, **NIX-SO$_2$Cl** should be prepared according to the reference[2] in advance.

On the whole, in a three-necked, round-bottomed flask provided with a magnetic stirrer was placed **HCy** (0.2 g, 0.38 mmol) and **NIX-SO$_2$Cl** (⩾ 1.52 mmol) and anhydrous dichloromethane. Then Cs_2CO_3 (⩾ 0.06 g) was added at 0 °C under N_2 atmosphere, after which the ice bath was removed to allow the reaction keep on at RT until the completion of the reaction. Then the solvent was removed *in vacuo* and the crude product was chromatographed on silica gel (MeOH/CH_2Cl_2) to give **HCyX** as a dark blue solid.

HCy1

Yield 42%. ^{1}H NMR (400 MHz, Chloroform-*d*) δ 8.68 (d, *J* = 1.8 Hz, 1H), 8.65 – 8.58 (m, 2H), 8.39 (d, *J* = 8.6 Hz, 1H), 8.36 (d, *J* = 8.6 Hz, 1H), 8.28 (dd, *J* = 8.5, 2.2 Hz, 1H), 8.25 (d, *J* = 2.2 Hz, 1H), 7.54 (t, *J* = 7.6 Hz, 2H), 7.48 (dd, *J* = 7.4, 5.6 Hz, 2H), 7.34 (d, *J* = 8.4 Hz, 1H), 7.21 (d, *J* = 2.2 Hz, 1H), 7.07 – 6.96 (m, 2H), 6.73 (d, *J* = 15.1 Hz, 1H), 4.59 (d, *J* = 7.5 Hz, 2H), 2.78 (d, *J* = 6.2 Hz, 2H), 2.71 (t, *J* = 6.0 Hz, 2H), 1.92 (s, 2H), 1.81 (s, 6H), 1.56 (t, *J* = 6.8 Hz, 3H). ^{13}C NMR (101 MHz, $CDCl_3$) δ 178.69, 173.14, 158.51, 152.98, 151.14, 149.39, 148.78, 148.24, 147.46, 146.57, 145.37, 142.50, 140.84, 134.28, 133.16, 131.89, 131.78, 130.89, 129.45, 129.15, 128.67, 128.37, 127.16, 125.29, 122.65, 121.71, 120.48, 118.52, 118.24, 115.97, 113.37, 110.12, 107.17, 51.34, 41.81, 29.58, 27.80, 24.16, 20.08, 13.21. HRMS (ESI$^+$): [M]$^+$, calcd. for $C_{33}H_{30}N_3O_8S^+$, 628.1748; found 628.1727. HRMS (ESI$^-$): [M]$^-$, calcd. for $C_6H_3N_2O_7S^-$, 246.9666; found 246.9776.

HCy2

Yield 47%. ^{1}H NMR (400 MHz, Chloroform-*d*) δ 8.61 (d, *J* = 15.0 Hz, 1H), 8.32 (dd, *J* = 8.3, 4.2 Hz, 2H), 8.13 (s, 1H), 8.08 (d, *J* = 8.2 Hz, 1H), 7.71 (d, *J* = 8.2 Hz, 1H), 7.66 (s, 1H), 7.50 (dt, *J* = 17.6, 7.0 Hz, 4H), 7.34 (d, *J* = 8.4 Hz, 1H), 7.20 (d, *J* = 2.2 Hz, 1H), 7.05 – 6.97 (m, 2H), 6.80 (d, *J* = 15.1 Hz, 1H), 4.63 (q, *J* = 7.2 Hz, 2H), 2.79 (t, *J* = 5.9 Hz, 2H), 2.69 (t, *J* = 6.0 Hz, 2H), 1.95 – 1.85 (m, 2H), 1.81 (s, 6H), 1.58 – 1.52 (m, 3H). ^{13}C NMR (101 MHz, $CDCl_3$) δ 178.62, 158.48, 152.95, 149.46, 148.72, 148.27, 146.47, 143.07, 142.50, 140.86, 137.58 (q, J = 34.7 Hz), 133.43, 131.71, 131.69 (q, J = 34.6 Hz), 131.26, 129.65 (q, J = 3.8 Hz), 129.44, 129.19, 128.65, 128.30, 127.46 (q, J = 3.6 Hz), 122.78 (q, J = 272.8 Hz), 122.56, 122.38 (q, J = 3.5 Hz), 121.70 (q, J = 273.9 Hz), 121.63, 119.91 (q, J = 3.9 Hz), 118.53, 115.99, 113.47, 110.07, 107.27, 51.27, 41.85, 29.55, 27.80, 24.14, 20.07, 13.24. ^{19}F NMR (376 MHz, Chloroform-*d*) δ -62.82, -63.35. HRMS (ESI$^+$): [M]$^+$, calcd. for $C_{34}H_{30}F_3N_2O_6S^+$, 651.1771; found 651.1770. HRMS (ESI$^-$): [M]$^-$, calcd. for $C_7H_3F_3NO_5S^-$, 269.9690; found 269.9694.

HCy3

Yield 59%. ^{1}H NMR (400 MHz, Chloroform-*d*) δ 8.61 (d, *J* = 15.2 Hz, 1H), 8.30 (t, *J* = 7.2 Hz, 2H), 8.19 – 8.13 (m, 2H), 7.73 (d, *J* = 8.2 Hz, 1H), 7.67 (s, 1H), 7.60 – 7.44 (m, 4H), 7.37 (d, *J* = 8.4 Hz, 1H), 7.22 – 7.16 (m, 1H), 7.06 – 7.00 (m, 2H), 6.70 (d, *J* = 15.2 Hz, 1H), 4.57 (q, *J* = 7.3 Hz, 2H), 2.75 (t, *J* = 6.0 Hz, 2H), 2.70 (t, *J* = 6.1 Hz, 2H), 1.90 (p, *J* = 6.1 Hz, 2H), 1.81 (s, 6H), 1.55 (t, *J* = 7.2 Hz, 3H). ^{13}C NMR (101 MHz, $CDCl_3$) δ 178.64, 158.71, 152.94, 149.49, 148.55, 148.38, 146.60, 143.99, 142.48, 140.80, 136.40, 134.15, 133.46, 131.76, 131.62, 131.52, 129.50, 129.45, 128.80, 128.37, 128.24, 126.17, 122.72, 121.66, 119.69, 118.66, 116.64, 115.83, 114.95, 113.33, 110.09, 106.88, 51.35, 41.77, 29.52, 27.81, 24.17, 20.08, 13.19. HRMS (ESI$^+$): [M]$^+$, calcd. for $C_{34}H_{30}N_3O_6S^+$, 608.1850; found 608.1854. HRMS (ESI$^-$): [M]$^-$, calcd. for $C_7H_3N_2O_5S^-$, 226.9768; found 226.9767.

HCy4

Yield 68%. ^{1}H NMR (400 MHz, Chloroform-*d*) δ 8.61 (d, *J* = 13.2 Hz, 2H), 8.17 (s, 1H), 7.90 (s, 1H), 7.73 (s, 1H), 7.58 – 7.43 (m, 4H), 7.41 (d, *J* = 8.4 Hz, 1H), 7.20 (d, *J* = 2.2 Hz, 1H), 7.11 (dd, *J* = 8.4, 2.2 Hz, 1H), 7.05 (s, 1H), 6.74 (d, *J* = 15.2 Hz, 1H), 4.61 (q, *J* = 7.2 Hz, 2H), 3.11 (s, 6H), 2.98 (s, 6H), 2.78 – 2.68 (m, 4H), 1.90 (p, *J* = 6.0 Hz, 2H), 1.81 (s, 6H), 1.55 (t, *J* = 7.1 Hz, 3H). ^{13}C NMR (101 MHz, $CDCl_3$) δ 178.52, 158.72, 152.95, 149.74, 147.35, 146.74, 146.45, 142.47, 140.87, 136.12, 134.46, 134.12, 133.33, 131.51, 129.60, 129.42, 128.71, 128.25, 127.10, 124.32, 122.59, 121.87, 121.46, 119.52, 118.77, 115.89, 113.38, 110.10, 107.00, 51.25, 42.84, 42.48, 41.77, 29.53, 27.83, 24.12, 20.10, 13.22. HRMS (ESI$^+$): [M]$^+$, calcd. for $C_{35}H_{35}N_4O_8S^+$, 671.2170; found 671.2195. HRMS (ESI$^-$): [M]$^-$, calcd. for $C_8H_8N_3O_7S^-$, 290.0088; found 290.0100.

HCy5

Yield 62%. ^{1}H NMR (400 MHz, Chloroform-*d*) δ 8.62 (d, *J* = 15.3 Hz, 1H), 8.51 (s, 1H), 8.08 (s, 1H), 7.95 (s, 1H), 7.84 (s, 1H), 7.58 – 7.45 (m, 4H), 7.40 (d, *J* = 8.5 Hz, 1H), 7.21 (d, *J* = 2.3 Hz, 1H), 7.09 (dd, *J* = 8.5, 2.3 Hz, 1H), 7.04 (s, 1H), 6.70 (d, *J* = 15.3 Hz, 1H), 4.57 (q, *J* = 7.2 Hz, 2H), 4.13 (s, 3H), 4.03 (s, 3H), 2.80 – 2.67 (m, 4H), 1.91 (p, *J* = 6.3 Hz, 2H), 1.82 (s, 6H), 1.56 (t, *J* = 7.2 Hz, 3H). ^{13}C NMR (101 MHz, $CDCl_3$) δ 178.64, 158.65, 155.01, 154.43, 152.95, 149.45, 146.53, 146.29, 142.48, 141.03, 140.81, 140.28, 140.10, 137.44, 133.87, 131.64, 129.43, 128.75, 128.36, 123.81, 122.66, 121.70, 121.64, 118.65, 117.79, 115.84, 115.65, 113.34, 110.14, 106.92, 58.52, 57.47, 51.34, 41.73, 29.53, 27.78, 24.11, 20.08, 13.18. HRMS (ESI$^+$): [M]$^+$, calcd. for $C_{34}H_{32}N_3O_9S^+$, 658.1854; found 658.1867. HRMS (ESI$^-$): [M]$^-$, calcd. for $C_7H_5N_2O_8S^-$, 276.9772; found 276.9768.

HCy6

Yield 74%. ^{1}H NMR (400 MHz, Chloroform-*d*) δ 8.58 (d, *J* = 15.0 Hz, 1H), 8.02 (d, *J* = 1.9 Hz, 1H), 7.84 (d, *J* = 8.8 Hz, 2H), 7.69 (d, *J* = 8.2 Hz, 1H), 7.56 – 7.41 (m, 4H), 7.33 (d, *J* = 8.1 Hz, 2H), 7.18 – 7.12 (m, 2H), 7.03 – 6.98 (m, 2H), 6.84 (d, *J* = 15.2 Hz, 1H), 4.66

(q, J = 7.1 Hz, 2H), 2.78 (t, J = 5.7 Hz, 2H), 2.67 (t, J = 5.8 Hz, 2H), 2.50 (s, 3H), 2.36 (s, 3H), 1.91 – 1.84 (m, 2H), 1.79 (s, 6H), 1.53 (t, J = 6.9 Hz, 3H). ^{13}C NMR (101 MHz, $CDCl_3$) δ 178.52, 158.40, 152.90, 149.78, 146.35, 144.27, 142.51, 141.59, 140.96, 139.40, 136.35, 132.41, 131.60, 130.56, 129.90, 129.40, 129.17, 128.45, 128.14, 127.89, 125.41, 122.74, 122.47, 121.40, 118.67, 116.14, 113.57, 110.10, 107.56, 51.17, 41.92, 29.57, 27.84, 24.14, 21.48, 21.24, 20.10, 13.30. HRMS (ESI$^+$): $[M]^+$, calcd. for $C_{34}H_{33}N_2O_6S^+$, 597.2054; found 597.2106. HRMS (ESI$^-$): $[M]^-$, calcd. for $C_7H_6NO_5S^-$, 215.9972; found 215.9984.

HCy7

Yield 78%. ^{1}H NMR (400 MHz, Chloroform-*d*) δ 8.58 (d, J = 14.7 Hz, 1H), 8.05 (d, J = 7.9 Hz, 1H), 7.90 (d, J = 7.6 Hz, 1H), 7.66 (s, 1H), 7.50 (tt, J = 19.2, 7.4 Hz, 5H), 7.32 (d, J = 8.3 Hz, 1H), 7.27 – 7.14 (m, 3H), 7.04 – 6.94 (m, 2H), 6.84 (d, J = 14.9 Hz, 1H), 4.72 – 4.62 (m, 2H), 2.79 (t, J = 5.8 Hz, 2H), 2.67 (t, J = 5.6 Hz, 2H), 2.55 (s, 3H), 2.34 (s, 3H), 1.91 – 1.84 (m, 2H), 1.80 (s, 6H), 1.53 (t, J = 6.4 Hz, 3H). ^{13}C NMR (101 MHz, $CDCl_3$) δ 178.51, 158.45, 152.89, 149.89, 148.54, 148.32, 146.38, 142.52, 140.96, 140.10, 136.94, 132.95, 132.16, 131.57, 131.29, 130.08, 129.40, 129.26, 128.47, 128.12, 125.50, 124.86, 122.82, 122.47, 121.36, 118.64, 116.15, 113.56, 110.12, 107.57, 51.16, 41.97, 29.59, 27.87, 24.20, 21.69, 20.88, 20.12, 13.32. HRMS (ESI$^+$): $[M]^+$, calcd. for $C_{34}H_{33}N_2O_6S^+$, 597.2054; found 597.2048. HRMS (ESI$^-$): $[M]^-$, calcd. for $C_7H_6NO_5S^-$, 215.9972; found 215.9990.

HCy8

Yield 78%. ^{1}H NMR (400 MHz, Chloroform-*d*) δ 8.56 (d, J = 15.2 Hz, 1H), 8.32 (d, J = 2.2 Hz, 1H), 8.20 – 8.13 (m, 2H), 8.10 (d, J = 8.6 Hz, 1H), 7.96 (d, J = 2.3 Hz, 1H), 7.89 (dd, J = 8.5, 2.4 Hz, 1H), 7.58 – 7.46 (m, 4H), 7.37 – 7.28 (m, 1H), 7.04 – 6.98 (m, 2H), 6.80 (d, J = 15.3 Hz, 1H), 6.71 (dd, J = 8.4, 2.3 Hz, 1H), 4.66 (q, J = 7.3 Hz, 2H), 2.94 (s, 3H), 2.81 (s, 3H), 2.76 (t, J = 6.0 Hz, 2H), 2.67 (t, J = 6.2 Hz, 2H), 1.87 – 1.83 (m, 2H), 1.78 (s, 6H), 1.56 (t, J = 7.2 Hz, 3H). ^{13}C NMR (101 MHz, $CDCl_3$) δ 178.51, 158.57, 152.88, 151.29, 150.89, 149.95, 147.67, 146.37, 142.36, 141.41, 140.79, 139.50, 138.84, 131.90, 131.44, 129.54, 129.39, 128.63, 128.53, 128.43, 127.44, 125.65, 122.58, 121.33, 121.31, 120.00, 118.32, 115.86, 113.44, 110.05, 107.17, 51.19, 41.85, 29.51, 27.80, 24.15, 20.94, 20.85, 20.03, 13.30. HRMS (ESI$^+$): $[M]^+$, calcd. for $C_{34}H_{33}N_2O_6S^+$, 597.2054; found 597.2067. HRMS (ESI$^-$): $[M]^-$, calcd. for $C_7H_6NO_5S^-$, 215.9972; found 215.9988.

HCy9

Yield 93%. ^{1}H NMR (400 MHz, Chloroform-*d*) δ 8.57 (d, J = 15.2 Hz, 1H), 8.19 (dd, J = 7.8, 1.3 Hz, 1H), 8.03 (dd, J = 7.9, 1.3 Hz, 1H), 7.95 (td, J = 7.7, 1.3 Hz, 1H), 7.89 (dd, J = 8.1, 1.3 Hz, 1H), 7.81 (td, J = 7.7, 1.4 Hz, 1H), 7.54 (d, J = 7.1 Hz, 1H), 7.52 – 7.35 (m, 6H), 7.32 (d, J = 8.5 Hz, 1H), 7.13 (d, J = 2.2 Hz, 1H), 7.02 – 6.97 (m, 2H), 6.79 (d, J = 15.3 Hz, 1H), 4.63 (q, J = 7.2 Hz, 2H), 2.76 (t, J = 6.0 Hz, 2H), 2.69 – 2.65 (m, 2H), 1.87 (p, J = 5.9 Hz, 2H), 1.79 (s, 6H), 1.53 (t, J = 7.1 Hz, 3H). ^{13}C NMR (101 MHz, $CDCl_3$) δ 178.54, 158.49, 152.86, 149.82, 148.55, 148.42, 146.40, 142.47, 140.90, 139.53, 136.42, 132.71, 132.13, 131.53, 130.79, 130.15, 129.63, 129.41, 129.30, 128.51, 128.20, 127.68, 125.10, 122.55, 121.40, 118.72, 115.99, 113.49, 110.03, 107.28, 51.22, 41.83, 29.53, 27.82, 24.09, 20.08, 13.25. HRMS (ESI$^+$): $[M]^+$, calcd. for $C_{33}H_{31}N_2O_6S^+$, 583.1897; found 583.1890. HRMS (ESI$^-$): $[M]^-$, calcd. for $C_6H_4NO_5S^-$, 201.9816; found 201.9826.

HCy10

Yield 82%. ^{1}H NMR (400 MHz, Chloroform-*d*) δ 8.58 (d, J = 15.2 Hz, 1H), 8.47 – 8.43 (m, 2H), 8.19 – 8.03 (m, 6H), 7.58 – 7.46 (m, 4H), 7.30 (d, J = 8.5 Hz, 1H), 7.07 (d, J = 2.2 Hz, 1H), 7.02 (s, 1H), 6.77 (d, J = 2.4 Hz, 1H), 6.76 – 6.73 (m, 1H), 4.63 (q, J = 7.3 Hz, 2H), 2.75 (t, J = 5.9 Hz, 2H), 2.69 (t, J = 6.1 Hz, 2H), 1.88 (p, J = 6.2 Hz, 2H), 1.79 (s, 6H), 1.57 (t, J = 7.1 Hz, 3H). ^{13}C NMR (101 MHz, $CDCl_3$) δ 178.55, 158.66, 153.28, 152.89, 151.24, 150.00, 147.83, 146.44, 142.39, 140.77, 140.57, 131.45, 130.06, 129.53, 129.49, 128.52, 128.45, 127.45, 124.66, 123.17, 122.65, 121.34, 118.68, 115.77, 113.38, 110.23, 106.98, 51.25, 41.80, 29.51, 27.81, 24.14, 20.06, 13.27. HRMS (ESI$^+$): $[M]^+$, calcd. for $C_{33}H_{31}N_2O_6S^+$, 583.1897; found 583.1919. HRMS (ESI$^-$): $[M]^-$, calcd. for $C_6H_4NO_5S^-$, 201.9816; found 201.9824.

Deduction I

Since light intensity is essentially a kind of energy flux density (as an approximation, herein emission peak broadening is disregarded),

$$F_{cat} = \Psi \cdot h\nu_{Flu} \cdot N_{Flu} = \Psi h\nu_{Flu} \cdot \phi \cdot N_{Abs} = \Psi h\nu_{Flu}\phi \cdot \frac{I_0 - I}{h\nu_{Abs}} = \Psi\phi \cdot \frac{I_0 - I}{I_0} \cdot \frac{I_0 \cdot \nu_{Flu}}{\nu_{Abs}} = \Psi\phi \cdot (1 - 10^{-A}) \cdot \frac{I_0 \cdot \nu_{Flu}}{\nu_{Abs}}$$

$$\approx 2.303\Psi\phi A \cdot \frac{I_0 \cdot \nu_{Flu}}{\nu_{Abs}} = 2.303\Psi\phi \cdot \frac{I_0 \cdot \nu_{Flu}}{\nu_{Abs}} \cdot \varepsilon bc = 2.303\Psi b \cdot \frac{I_0 \cdot \nu_{Flu}}{\nu_{Abs}} \cdot \varepsilon\phi \cdot c$$

$$\because v_0 = \frac{dc}{dt}, \therefore\ c = \int_0^t v_0 dt = v_0 t = \frac{k_{cat}[E]_0[S]_0}{K_m + [S]_0} \cdot t$$

$$\therefore F_{cat} \approx 2.303\Psi b \cdot \frac{I_0 \cdot \nu_{Flu}}{\nu_{Abs}} \cdot \varepsilon\phi \cdot \frac{k_{cat}[E]_0[S]_0}{K_m + [S]_0} \cdot t$$

$$if\ [S]_0 \ll K_m, then\ F_{cat} \approx 2.303\Psi b \cdot \frac{I_0 \cdot \nu_{Flu}}{\nu_{Abs}} \cdot \varepsilon\phi \cdot \frac{k_{cat}}{K_m}[E]_0[S]_0 \cdot t$$

$$if\ [S]_0 \gg K_m, then\ F_{cat} \approx 2.303\Psi b \cdot \frac{I_0 \cdot \nu_{Flu}}{\nu_{Abs}} \cdot \varepsilon\phi \cdot k_{cat}[E]_0 \cdot t$$

Any way, $F_{cat} \propto S_{Photoluminescence\ mechanism} \cdot S_{recognition\ or\ reaction\ mechanism}$

Similarly, for a nonenzymatic reaction between GSH and a substrate (*i.e.* probe): $F_{nonc} \approx 2.303\Psi b \cdot \frac{I_0 \cdot \nu_{Flu}}{\nu_{Abs}} \cdot \varepsilon\phi \cdot k_{nonc}[GSH]_0[S]_0 \cdot t$

F_{cat}: fluorescence intensity of enzymatic reaction mixture
Ψ: the overall fluorescence collection efficiency of the apparatus
h: Planck constant
ν_{Flu}: frequency of the emitted light
ν_{Abs}: frequency of the excitation light
$h\nu$: energy of per photon
N_{Flu}: the number of fluorescent photons through a unit area emitted by the dye per unit time
ϕ: fluorescence quantum yield
N_{Abs}: the number of photons absorbed by the dye per unit time and per unit area
I_0: the initial intensity of the incident light, namely the initial intensity of the excitation light
I: the remaining intensity of the light which has underwent absorption by the dye molecules
A: absorbance
ε: molar extinction coefficient
b: optical path length
c: the molar concentration of the luminescent substance (the dye molecules)
$\varepsilon\phi$: the brightness of the dye
v_0: the initial reaction rate
t: reaction time
k_{cat}: catalytic constant
K_m: Michaelis constant
$[E]_0$: the initial molar concentration of the enzyme
$[S]_0$: the initial molar concentration of the substrate (herein the probe)
k_{nonc}: apparent second-order reaction rate constant
$[GSH]_0$: the initial molar concentration of GSH
F_{nonc}: fluorescence intensity of nonenzymatic reaction mixture

Notes:

[a] In the derivation of "$\Psi\phi \cdot \frac{I_0 - I}{I_0} \cdot \frac{I_0 \cdot \nu_{Flu}}{\nu_{Abs}} = \Psi\phi \cdot (1 - 10^{-A}) \cdot \frac{I_0 \cdot \nu_{Flu}}{\nu_{Abs}}$", the definition of absorbance "$A = -lg\frac{I}{I_0}$" is used.

[b] $1 - 10^{-A}$ and $(\ln 10)A$ are the equivalent infinitesimal when $A \to 0$, so $1 - 10^{-A} \approx 2.303A$. And the working concentration of the probe is generally several to tens of μM, which makes A rather small (close to zero), and hence meet the requirement for aforementioned approximation.

[c] In the derivation of "$2.303\Psi\phi A \cdot \frac{I_0 \cdot \nu_{Flu}}{\nu_{Abs}} = 2.303\Psi\phi \cdot \frac{I_0 \cdot \nu_{Flu}}{\nu_{Abs}} \cdot \varepsilon bc$", the Lambert–Beer law "$A = \varepsilon bc$" is used.

[d] In the derivation of "$\int_0^t v_0 dt = v_0 t = \frac{k_{cat}[E]_0[S]_0}{K_m + [S]_0} \cdot t$", the hypothesis that the initial rate remains unchanged in a short period since the initiation of reactions and Michaelis-Menten equation are applied. For the former, no matter in the study of kinetic measurements or in the incubation of cells with probes, administration in a relatively short time assures this deduction reasonable.

[e] The fluorescence intensity is proportional to signals arising from both photoluminescence mechanism (PM) and recognition or reaction mechanism (RM). For the former mechanism, a specific dye with high molar extinction coefficient and/or high fluorescence quantum yield, namely high brightness, is favorable to probe design as the fluorophore. For the latter mechanism, a probe with large catalytic constant k_{cat} and small Michaelis constant K_m is beneficial. In practice, k_{cat} is more important than K_m. And certainly, increasing the intensity of excitation light I_0, improving the overall fluorescence collection efficiency of the apparatus Ψ or prolonging the reaction or incubation time t can also afford a brighter fluorescence signal, and this is the very reason why one must keep these parameters consistent strictly in a set of experiments addressing the comparison of the fluorescence intensities from different samples.

[f] One of the main conclusions in this work is that although herein the property of a probe can be artificially divided into two parts, namely the PM and RM, the probe should be treated as an entirety. In other words, the RM is related not only to the receptor unit but also to the fluorophore, and so is the PM.

Deduction II

If x_w stands for the portion of a certain probe dissolved in the water, and x_o stands for the portion dissolved in the octanol, then $x_w + x_o = 1$ and $\log P = \log\frac{x_o}{x_w} = \log\frac{1-x_w}{x_w}$. Thus, we get $P = \frac{1-x_w}{x_w}$, namely

$$x_w \overset{\text{def}}{=} \frac{1}{1+P}$$

Then, what if the fluorophore's hydrophilicity affects remarkably the encounter between probe and GSH molecules and thus the apparent nonenzymatic reaction rate constant k_{nonc}? Since GSH is a hydrophilic and polar substance, the effective initial concentration of the probe that can be "felt" readily by GSH molecules ($[S]_{0,eff}$) should multiply the apparent one ($[S]_0$) by a factor that is proportional to x_w, and herein for convenience we can just directly take $[S]_{0,eff} = x_w \cdot [S]_0$. Hence, the nonenzymatic reaction rate $v_{0,nonc} = k_{nonc,true} \cdot [S]_{0,eff}[GSH]_0 = k_{nonc,true} \cdot x_w \cdot [S]_0[GSH]_0$ and $v_{0,nonc} = k_{nonc} \cdot [S]_0[GSH]_0$, in which $k_{nonc,true}$ is the true nonenzymatic reaction rate constant that is determined only by the nature, namely the chemical structure, of the receptor unit of one certain probe and k_{nonc} is the apparent one that is measured by the kinetic study, and $[GSH]_0$ is the initial concentration of glutathione. So, $k_{nonc} = k_{nonc,real} \cdot x_w$. It should be noted that, this deduction is based on the hypothesis that it is the hydrophilicity instead of the electron-deficiency of the fluorophore that bring about the differences in reactivity between corresponding **HCy-**based and **NI-**based probes. Therefore, if this hypothesis were true, when regarding different receptor units, the amplifications of k_{nonc} induced by the replacement of **NI** with the more hydrophilic fluorophore **HCy** should be positively correlated with the amplifications of x_w. In other words, $R_1 = k_{nonc}$ (**HCy**-)/k_{nonc} (**NI**-) should be positively correlated with $R_3 = x_w$ (**HCy-**)/x_w (**NI-**) = (1 + P (**NI-**))/(1 + P (**HCy-**)), which is not the actual situation as shown in relevant tables. Hence, it is not the fluorophore's hydrophilicity that causes the increased reactivities of **HCy-**based probes.

Deduction III

As an approximation, suppose regardless of the specific structure of the receptor unit, the increment of the local electrophilicity ω_k (*i.e.* $\Delta\omega_k$,) induced by the replacement of **NI** with the more electron-deficient fluorophore **HCy** is a constant, and this assumption is demonstrated to be reasonable as follows:

$$R_4 = \frac{\omega_k\ (\mathbf{HCy}-)}{\omega_k\ (\mathbf{NI}-)} = \frac{\omega_k + \Delta\omega_k}{\omega_k} = 1 + \frac{\Delta\omega_k}{\omega_k}$$

If $\Delta\omega_k$ is a constant, when increasing the electriphilicity of the receptor unit, namely enlarging ω_k, R_4 should decrese monotonically, which is none other than the actual case reflected by the trend in Table 3 in the main text.

Then, without loss of generality, we assume the variation of k_{nonc} induced by the replacement of either a fluorophore or a receptor unit can be described by formulas of the same pattern. For example, one may choose the formula pattern describing **HCy-**based probes (refer to Figure S9),

$$k_{nonc} = a\omega_k + b$$

where a and b are constants, and a > 0.

$$R_1 = \frac{k_{nonc}\ (\mathbf{HCy}-)}{k_{nonc}\ (\mathbf{NI}-)} = \frac{a(\omega_k + \Delta\omega_k) + b}{a\omega_k + b} = \frac{a\omega_k + b + a\Delta\omega_k}{a\omega_k + b} = 1 + \frac{a\Delta\omega_k}{a\omega_k + b}$$

Hence, when increasing the electriphilicity of the receptor unit, namely enlarging ω_k, R_1 should decrese monotonically, which is exactly consistent with the actual case reflected by the trend in Table 1 in the main text.

Alternatively, one can also choose the formula pattern describing **NI-**based probes,[2]

$$k_{nonc} = e^{\alpha}{\omega_k}^{\beta}$$

where α and β are positive constants.

$$R_1 = \frac{k_{nonc}\ (\mathbf{HCy}-)}{k_{nonc}\ (\mathbf{NI}-)} = \frac{e^{\alpha}(\omega_k + \Delta\omega_k)^{\beta}}{e^{\alpha}{\omega_k}^{\beta}} = \frac{(\omega_k + \Delta\omega_k)^{\beta}}{{\omega_k}^{\beta}} = (1 + \frac{\Delta\omega_k}{\omega_k})^{\beta}$$

And the same conclusion can be drawn.

Taken together, it's rational to infer that the larger reactivities of **HCy-**based probes relative to **NI-**based ones are probably caused by the larger electron-deficiency of **HCy**.

Supplementary Figures, Schemes and Tables

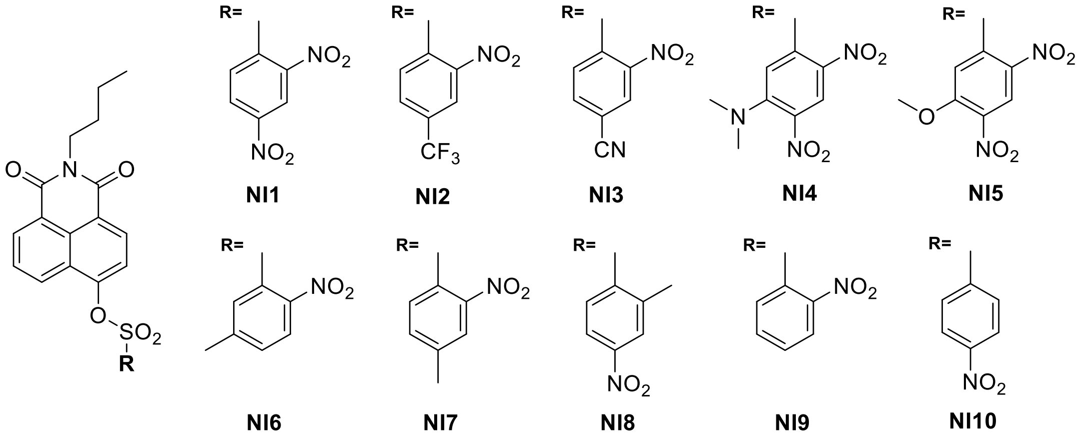

Figure S1. Chemical structures of naphthalimide (**NI**)-based probes **NI1-10**.

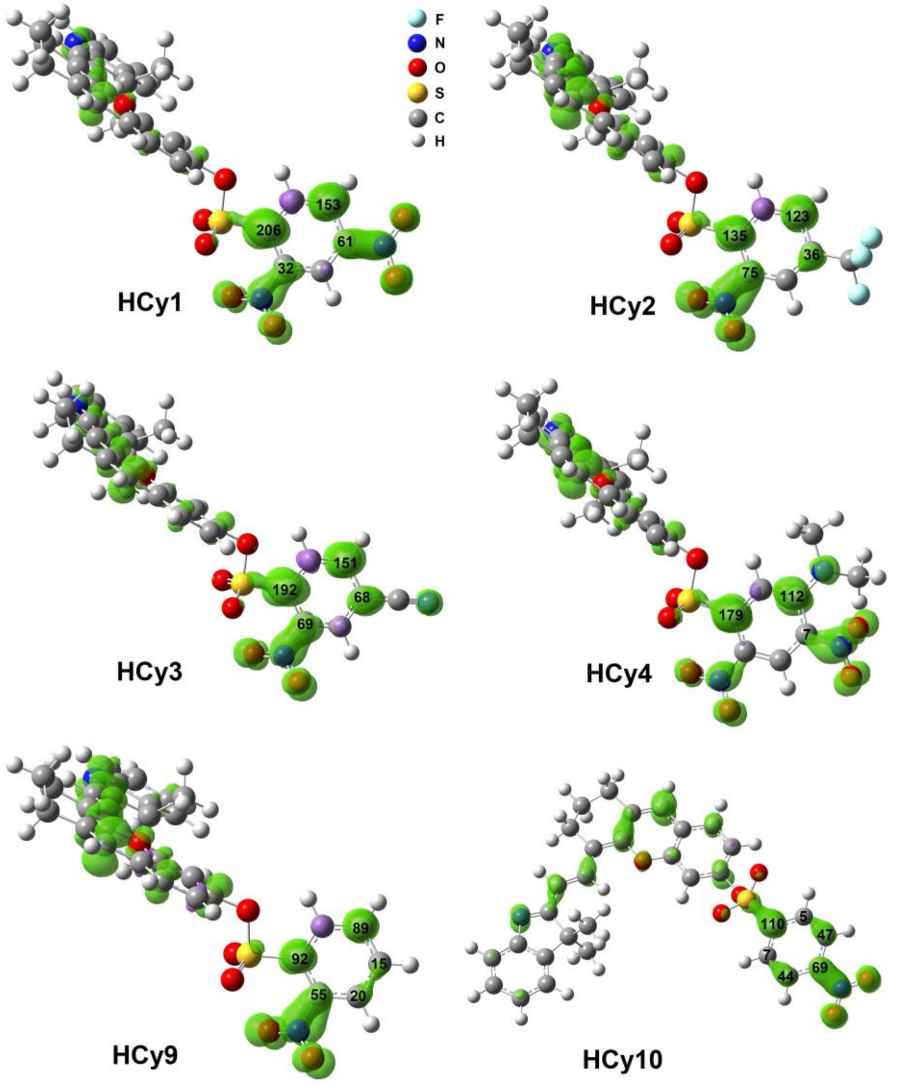

Figure S2. Spin density distributions of the anion radicals of probe models intimately related to this work. Respective positive P_k^{+} value (amplified by a factor of 1,000) of the carbon atoms in the nitrobenzene ring is marked, with positive and negative spin density colored by green and purple, respectively. Negative spin density or P_k^{+} values are herein regarded as meaningless.[18,19] Isodensity value = 0.002.

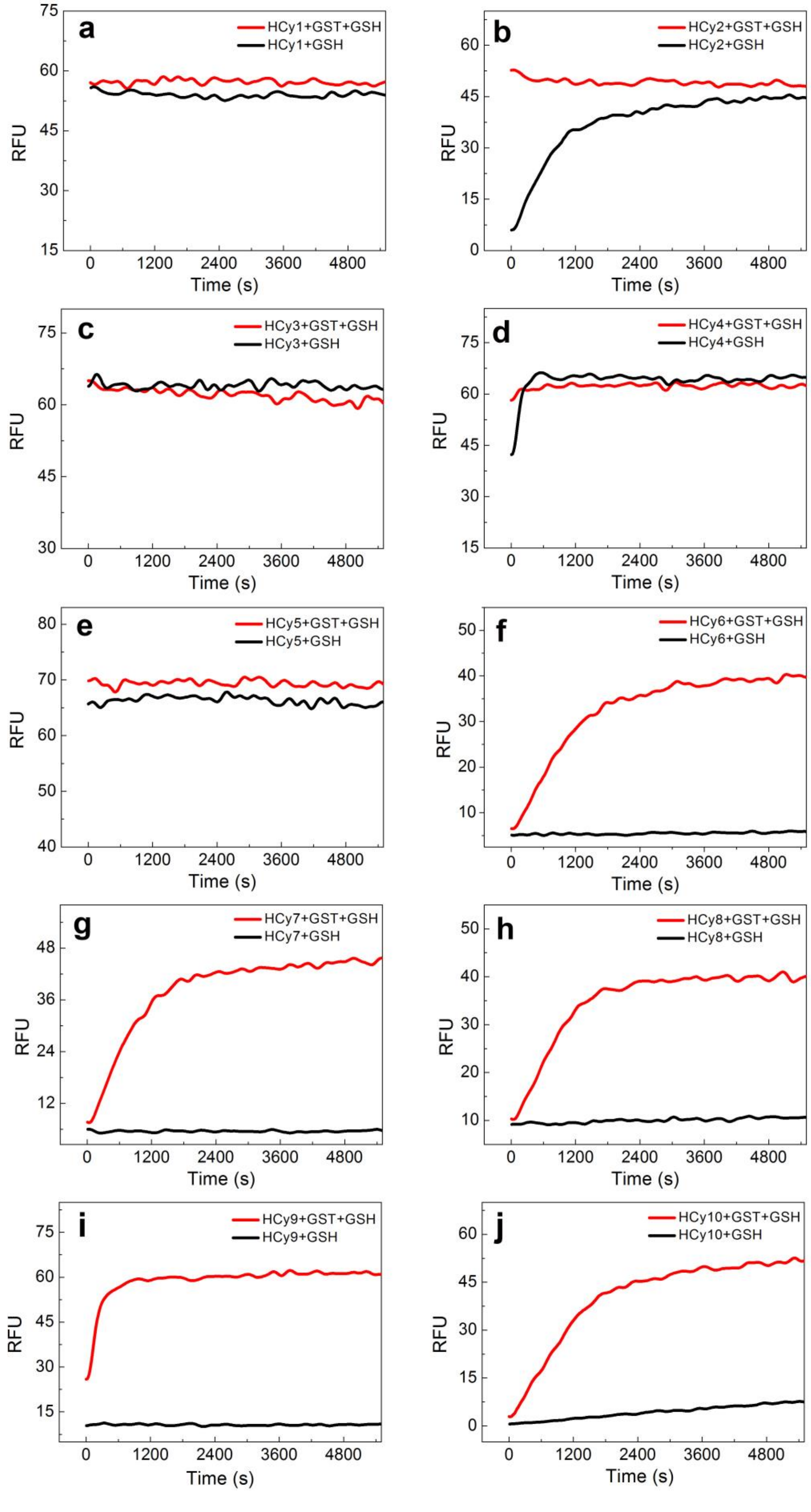

Figure S3. Respective time course of fluorescence intensity of probes **HCy1−10** (20 μM) in HEPES buffer (50 mM, 0.5% DMSO, pH 7.4) with or without GSTs (12.5 μg/mL) from equine liver in the presence of GSH (10 mM). $\lambda_{ex/em}$ = 650/700 nm. GSTs were premixed with GSH for 10 min prior to the addition of probes.

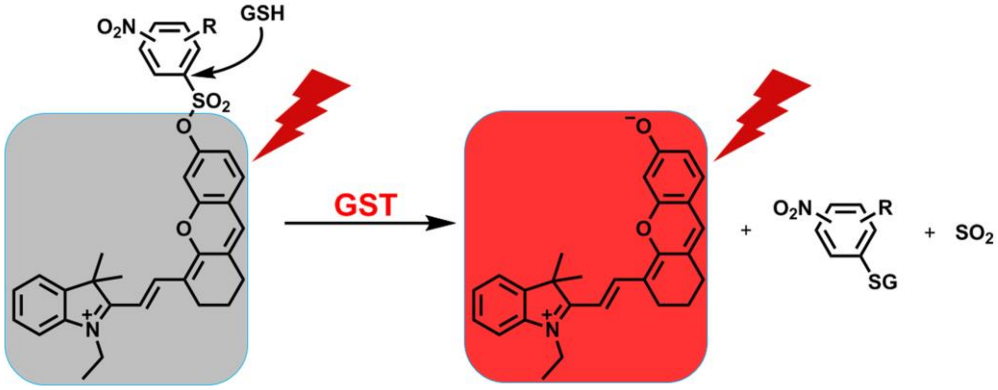

Scheme S1. GST detection mechanism of the **HCy**-based probes.

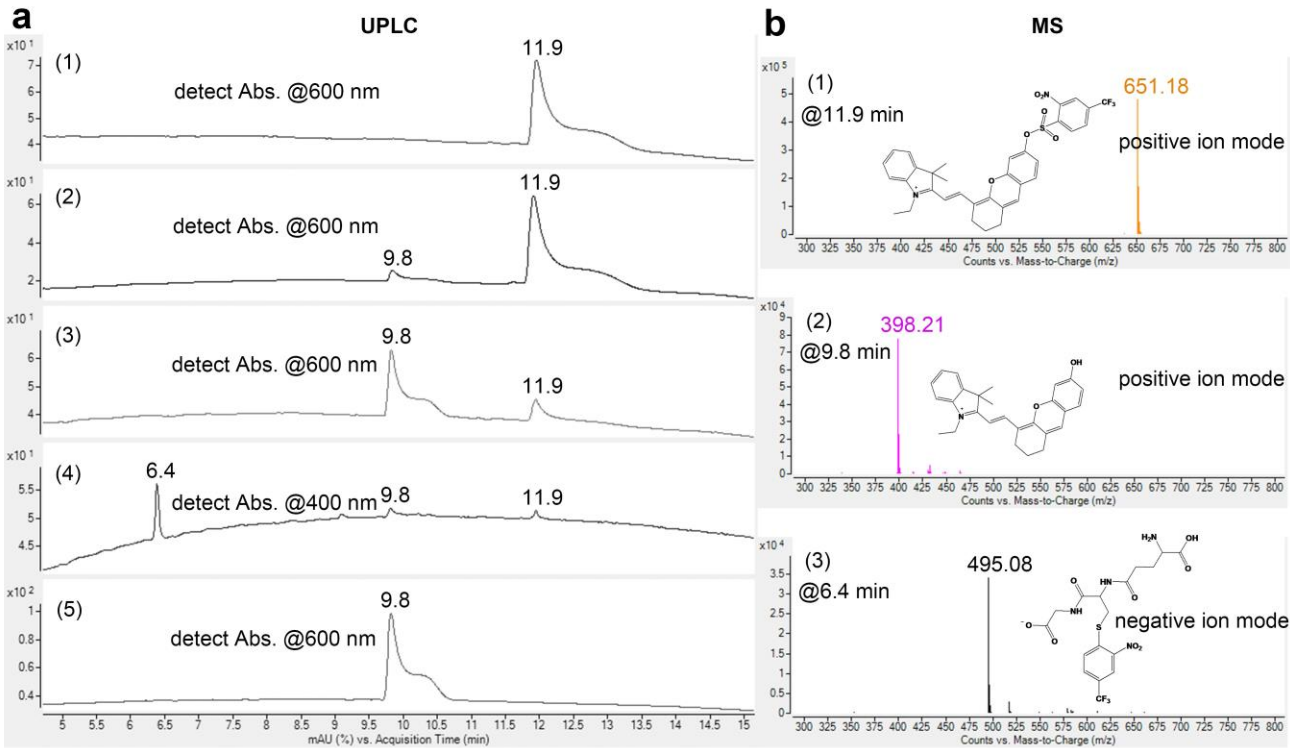

Figure S4. UPLC-MS analysis of the GST-catalyzed reaction between **HCy2** and GSH. (a) UPLC traces of (1) **HCy2**, (2) reaction mixture at 2 min after addition of GSTs (6.875 μg/mL) from equine liver, (3,4) reaction mixture at 10 h after addition of GSTs (12.5 μg/mL) from equine liver and (5) **HCy**. (b) Mass spectra (MS) analysis of the peaks in UPLC. (1) The peak at 11.9 min corresponds to **HCy2** ($[M]^+$, calcd. for $C_{34}H_{30}F_3N_2O_6S^+$, 651.1771; found 651.18). (2) The peak at 9.8 min corresponds to **HCy** ($[M]^+$, calcd. for $C_{27}H_{28}NO_2^+$, 398.2115; found 398.21). (3) The peak at 6.4 min corresponds to the glutathione-conjugate **GS-2** ($[M-H]^-$, calcd. for $C_{17}H_{18}F_3N_4O_8S^-$, 495.0803; found 495.08).

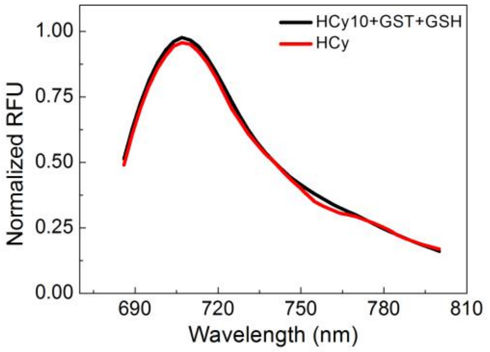

Figure S5. Fluorescence spectra of **HCy** and the GST catalyzed reaction product with **HCy10** and GSH being the reactants (prolong the time until the reaction nearly fully completed). Fluorescence intensity is normalized. λ_{ex} = 650 nm.

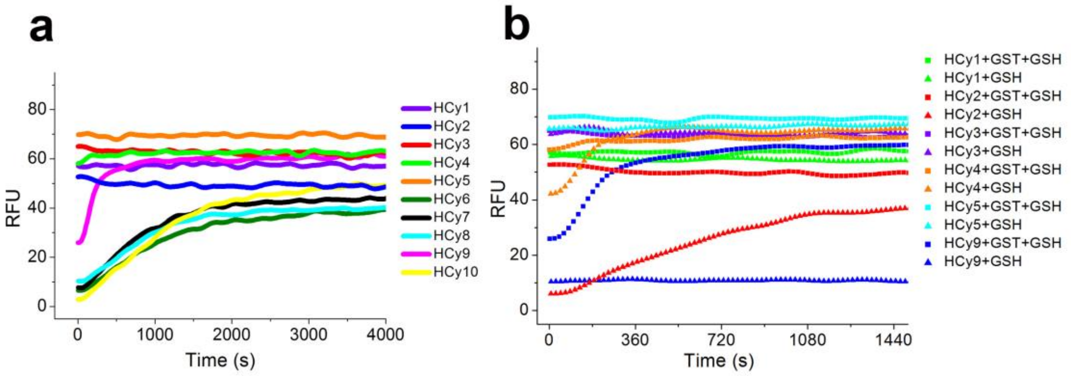

Figure S6. Comparison of probes in terms of (a) sensitivity and (b) background noise. Data in Figure S3 were reprocessed to give this figure.

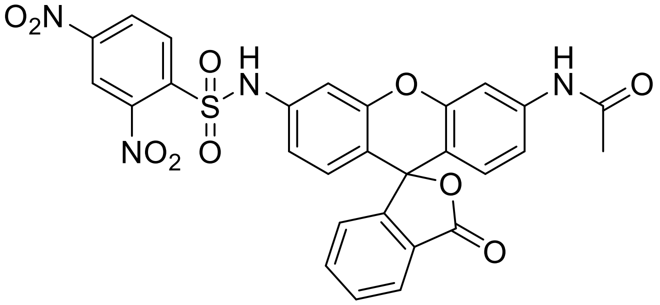

DNs-AcRh

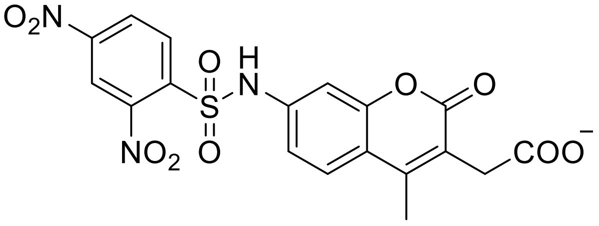

DNs-Coum

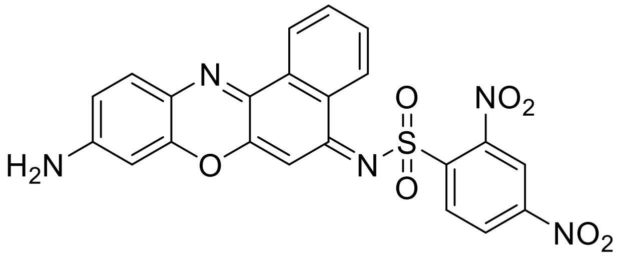

DNs-CV

Figure S7. Chemical structures of **DNs-AcRh**, **DNs-Coum** and **DNs-CV** reported in the literature.[1]

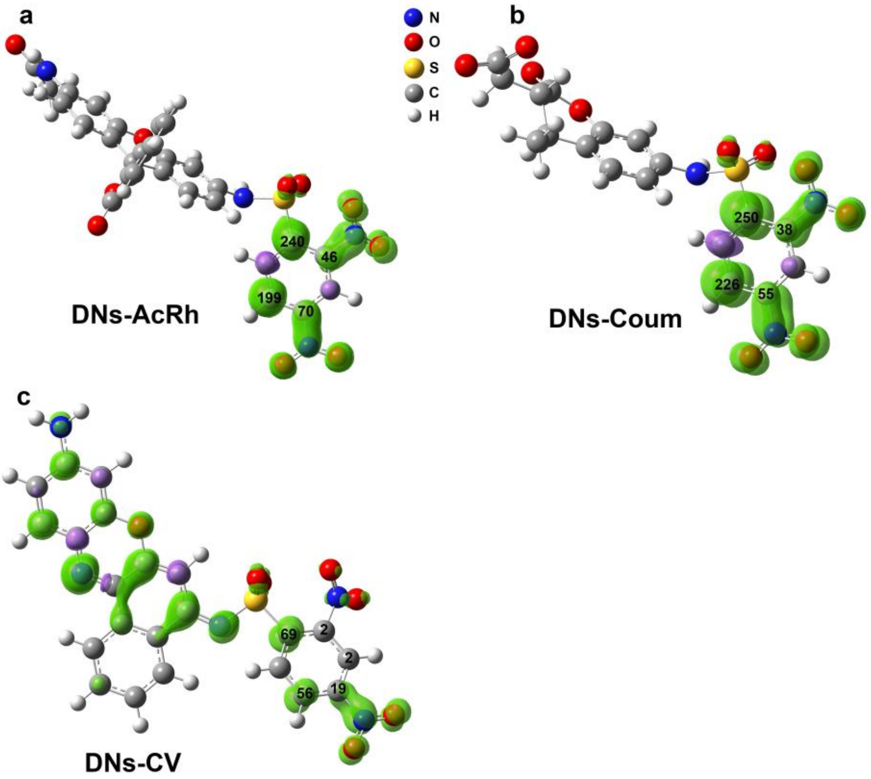

Figure S8. Spin density distributions of the anion radicals of (a) **DNs-AcRh**, (b) **DNs-Coum** and (c) **DNs-CV** appeared in the literature[1]. Respective positive P^{+}_{k} value (amplified by a factor of 1,000) of the carbon atoms in the nitrobenzene ring is marked, with positive and negative spin density colored by green and purple, respectively. Negative spin density or P^{+}_{k} values are herein regarded as meaningless.[18,19] Isodensity value = 0.002.

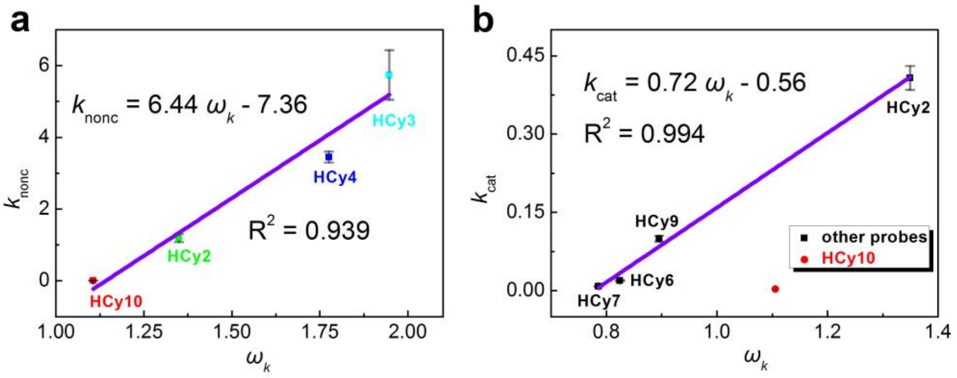

Figure S9. Structure-activity relationships between (a) nonenzymatic or (b) enzymatic kinetic parameters and the local electrophilicity ω_k of the α-carbon for the probes. The enzyme in (b) is GSTA1-1.

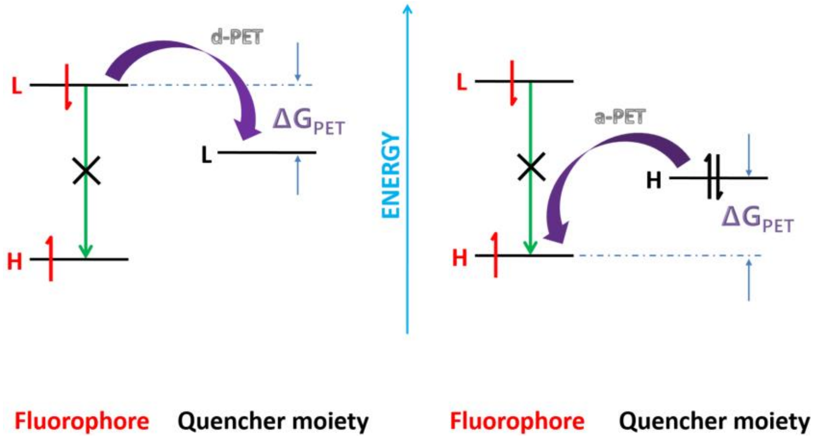

Scheme S2. Schematic diagram for the PET process in terms of frontier molecular orbitals. ΔG_{PET} means the driving force of PET. According to the role of the fluorophore as an electron donor or acceptor, two types of PET, namely d-PET and a-PET, are defined.

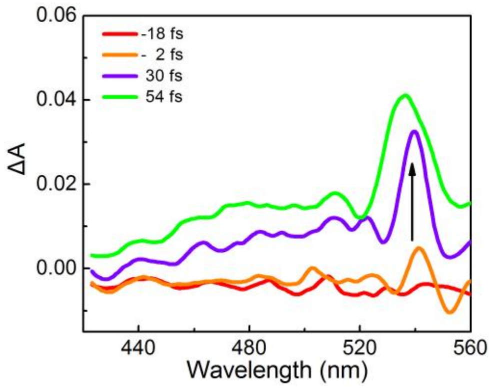

Figure S10. Femtosecond TA spectra recorded at different time-delays (-18−54 fs) after a femtosecond laser excitation (630 nm).

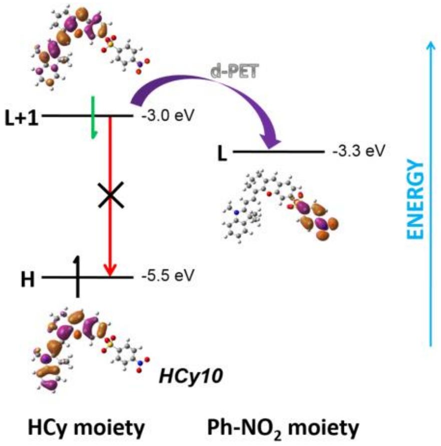

Figure S11. TD-DFT calculations on the electronic transitions of **HCy10** in DMSO at the B3LYP/aug-cc-pVDZ level.

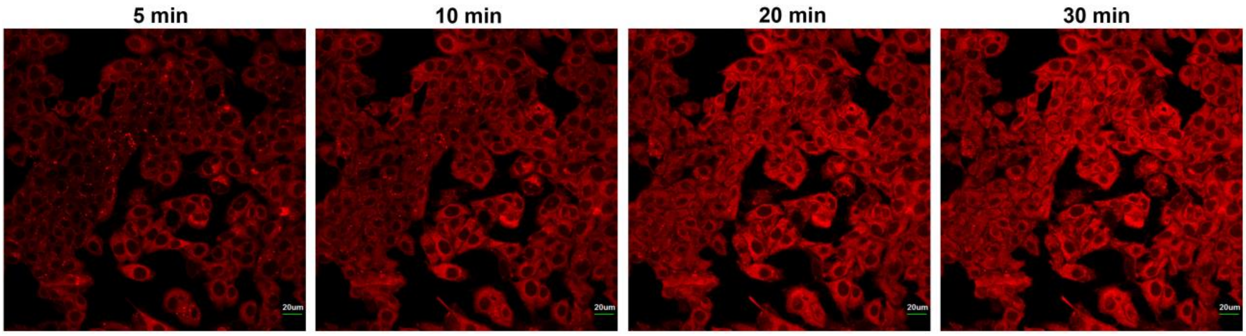

Figure S12. Fluorescence images of HepG2 cells at different time points after incubation with 10 μM **HCy2** in HEPES buffer (20 mM, 0.25% DMSO, 5% glucose, pH 7.4). Images were acquired upon excitation at 633 nm with a 40× objective. λ_{em} = 680−780 nm. Scale bar = 20 μm.

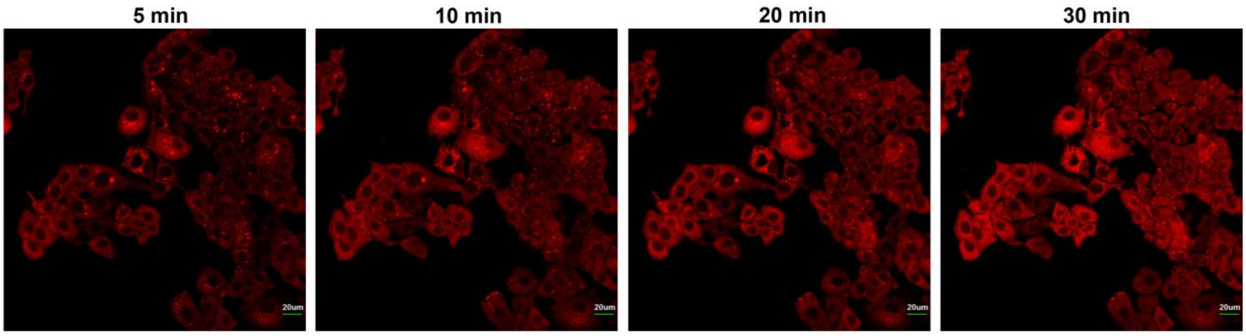

Figure S13. Fluorescence images of HepG2 cells at different time points after incubation with 10 μM **HCy9** in HEPES buffer (20 mM, 0.25% DMSO, 5% glucose, pH 7.4). Images were acquired upon excitation at 633 nm with a 40× objective. λ_{em} = 680−780 nm. Scale bar = 20 μm.

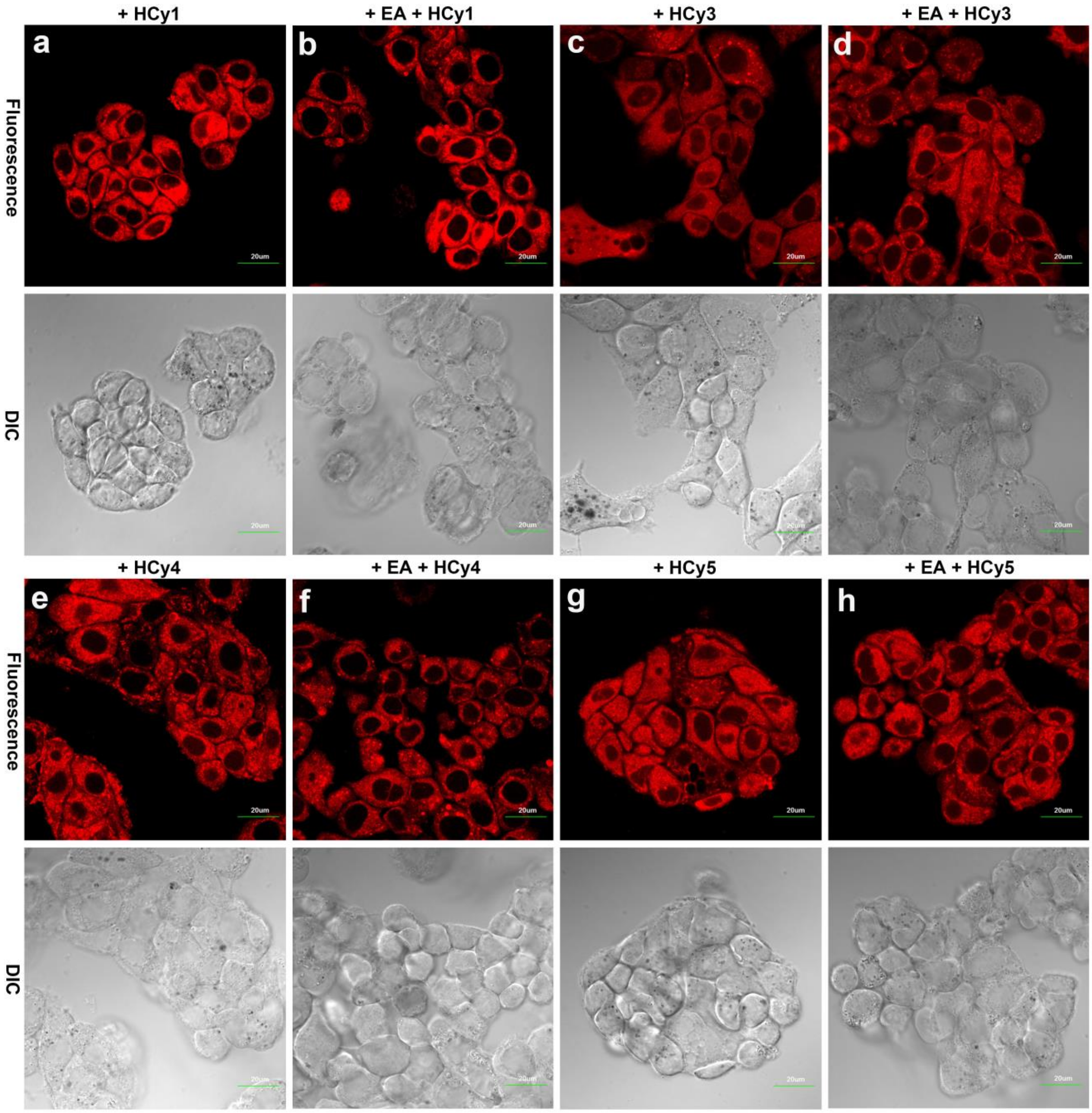

Figure S14. Fluorescence imaging of HepG2 cells incubated with 10 μM (a,b) **HCy1**, (c,d) **HCy3**, (e,f) **HCy4** and (g,h) **HCy5** in HEPES buffer (20 mM, 0.25% DMSO, 5% glucose, pH 7.4) for 30 min, respectively, with ((b,d,f,h) or without (a,c,e,g) pretreatment by EA. λ_{ex} = 633 nm. Scale bar = 20 μm.

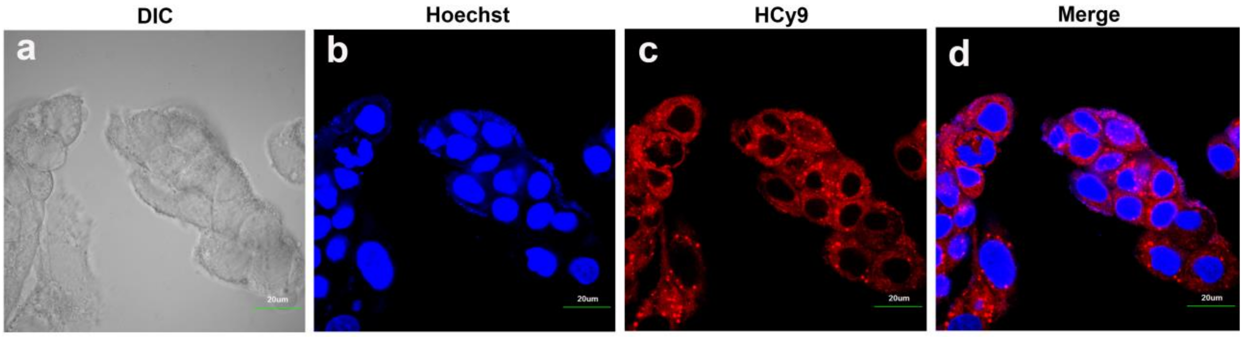

Figure S15. Fluorescence images of HepG2 cells co-incubated with Hoechst 33342 (a commercial nucleus-staining dye) and **HCy9**. (a) DIC image. (b) Hoechst 33342. (c) **HCy9**. (d) Merged image of (b) and (c). The excitation wavelengths for (b) and (c) were 405 and 633 nm, respectively, and the corresponding emissions were collected at 440–540 nm and 680–780 nm. Scale bar = 20 μm. Representative images from repeated experiments are shown.

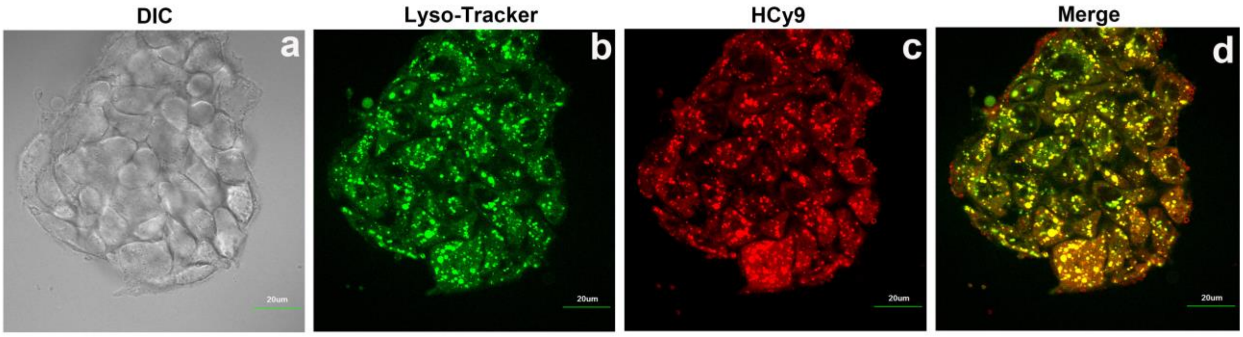

Figure S16. Fluorescence images of HepG2 cells co-incubated with Lyso-Tracker Green and **HCy9**. (a) DIC image. (b) Lyso-Tracker Green. (c) **HCy9**. (d) Merged image of (b) and (c). The excitation wavelengths for (b) and (c) were 488 and 633 nm, respectively, and the corresponding emissions were collected at 500–560 nm and 680–780 nm. Scale bar = 20 μm. Representative images from repeated experiments are shown.

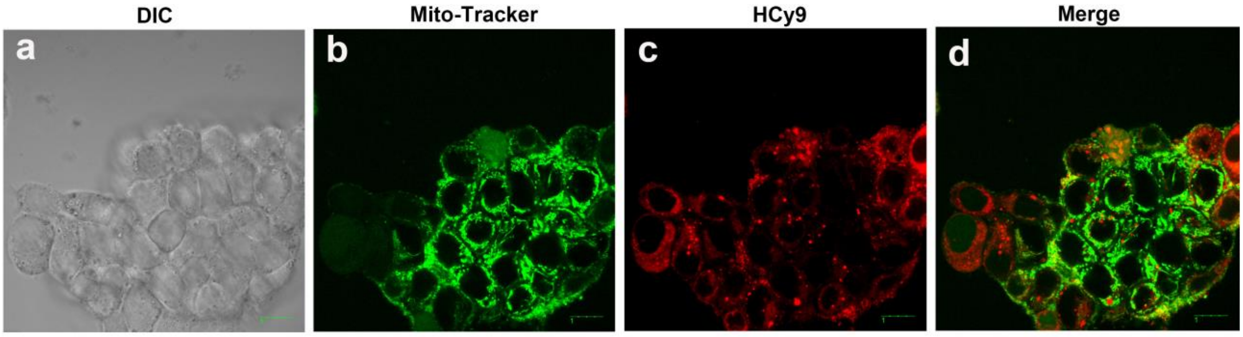

Figure S17. Fluorescence images of HepG2 cells co-incubated with Mito-Tracker Green and **HCy9**. (a) DIC image. (b) Mito-Tracker Green. (c) **HCy9**. (d) Merged image of (b) and (c). The excitation wavelengths for (b) and (c) were 488 and 633 nm, respectively, and the corresponding emissions were collected at 500–560 nm and 680–780 nm. Scale bar = 20 μm. Representative images from repeated experiments are shown.

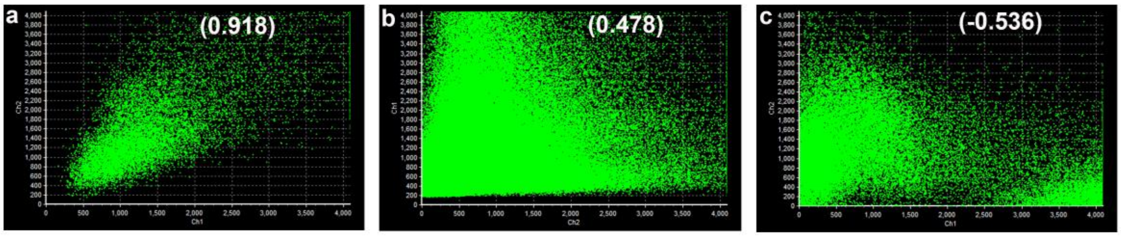

Figure S18. Scatter diagrams for colocalization analysis of **HCy9** and (a) Lyso-Tracker Green, (b) Mito-Tracker Green, or (c) Hoechst 33342. The numbers in parentheses are corresponding Pearson correlation coefficients.

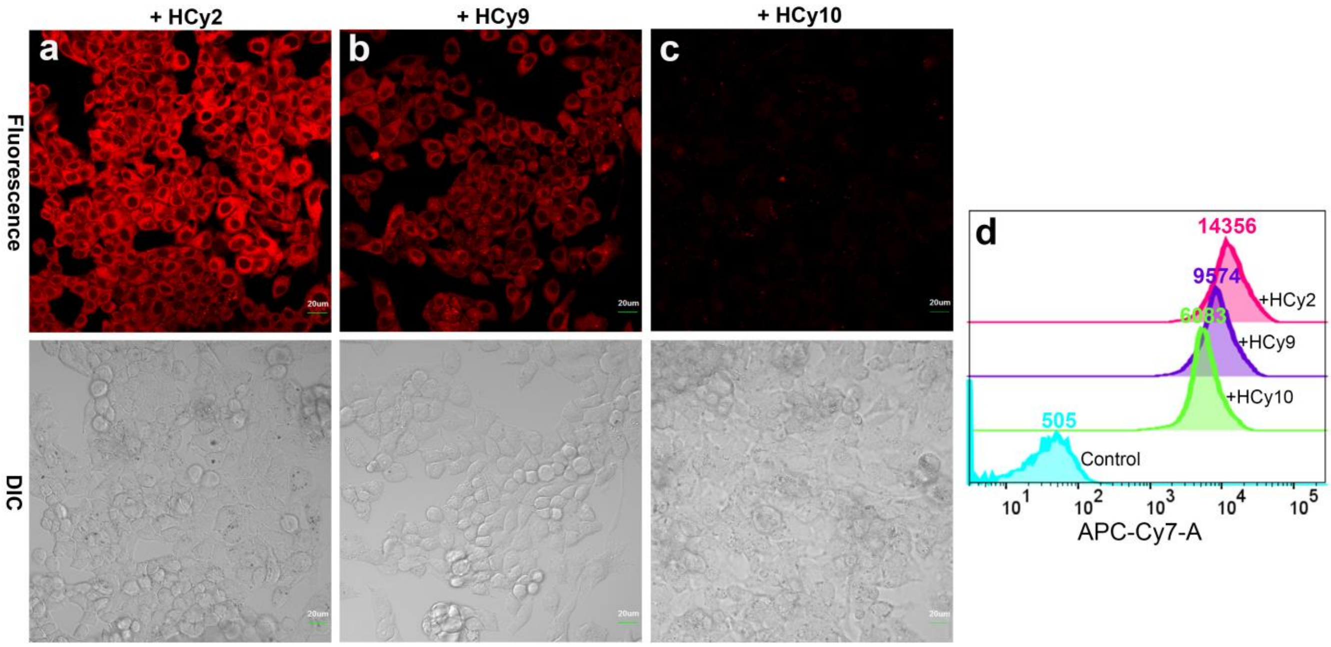

Figure S19. Fluorescence imaging of HepG2 cells incubated with 10 μM (a) **HCy2**, (b) **HCy9** or (c) **HCy10** for 30 min with 40× objective, respectively, and (d) flow cytometric analysis of HepG2 cells incubated with the same probes for 30 min. In panels a−c, λ_{ex} = 633 nm, λ_{em} = 680−780 nm, scale bar = 20 μm, and representative images from repeated experiments are shown. In panel d, λ_{ex} = 633 nm, and λ_{em} = 680−780 nm.

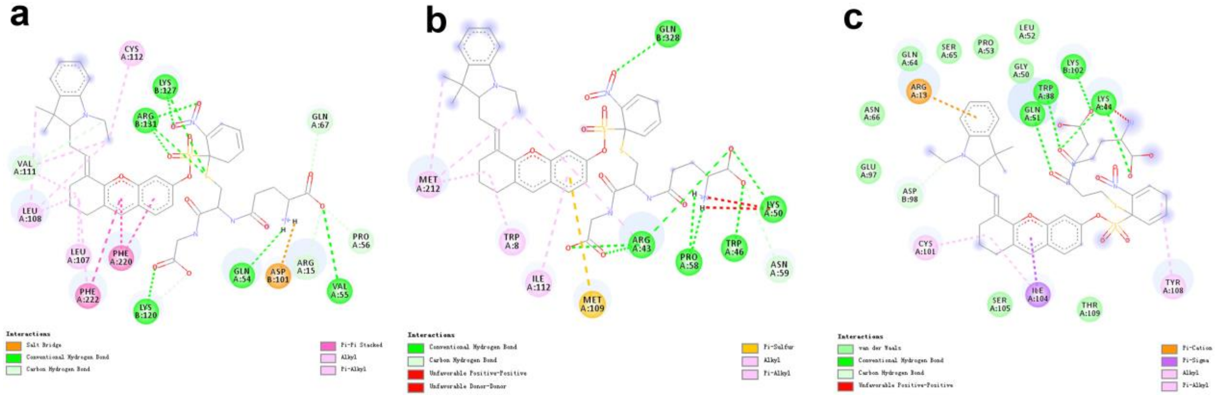

Figure S20. 2D visualization of active-site interactions between **GS-HCy9** σ complex and amino acid residues of (a) GSTA1-1, (b) GSTM1-1 and (c) GSTP1-1, respectively, by DS Visualizer analysis. H-bonds are indicated with green dotted lines.

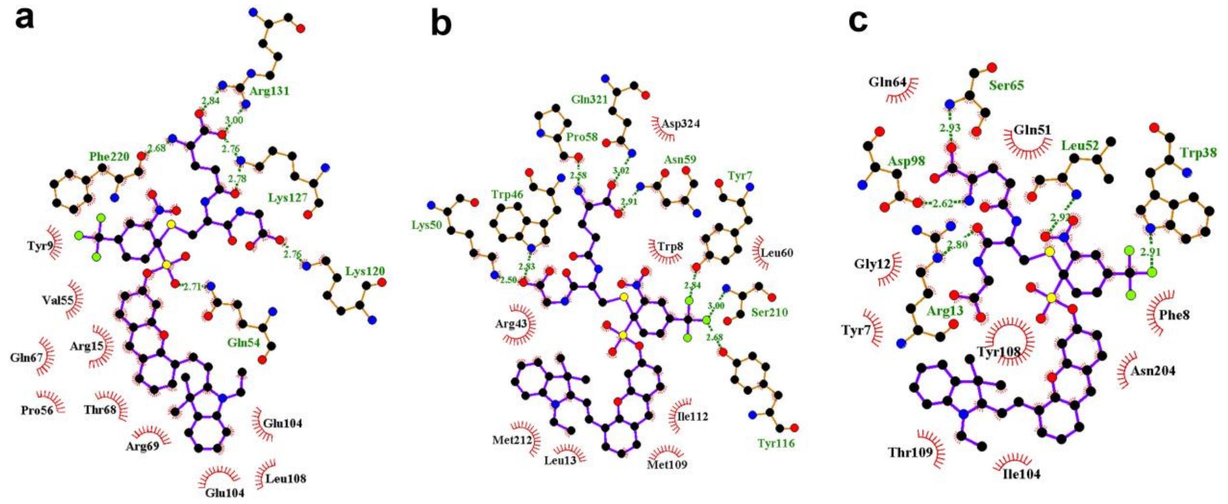

Figure S21. Docking simulations of **GS-HCy2** σ complex into (a) GSTA1-1, (b) GSTM1-1 and (c) GSTP1-1, respectively, with Ligplot+ analysis of active-site interactions shown. C, N, O, S and F atoms are shown in black, blue, red, yellow and green, respectively. H-bonds and the hydrophobic interactions between **GS-HCy2** σ complex and amino acid residues of GST are indicated with green dotted lines and red curves respectively.

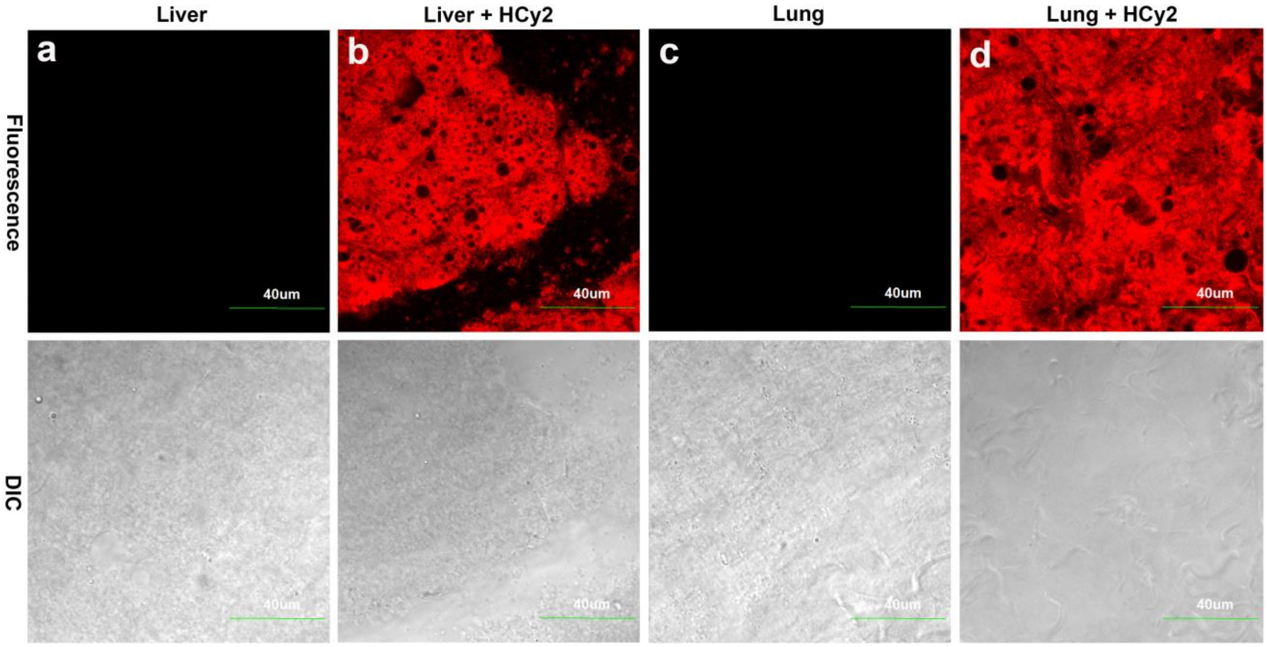

Figure S22. Fluorescence imaging of mice (a,b) liver and (c,d) lung tissue sections incubated with or without 20 μM **HCy2** in HEPES buffer (20 mM, 0.5% DMSO, 5% glucose, pH 7.4) at a depth of 50 μm with a 100× objective. Scale bar = 40 μm. λ_{ex} = 633 nm. λ_{em} = 680−780 nm.

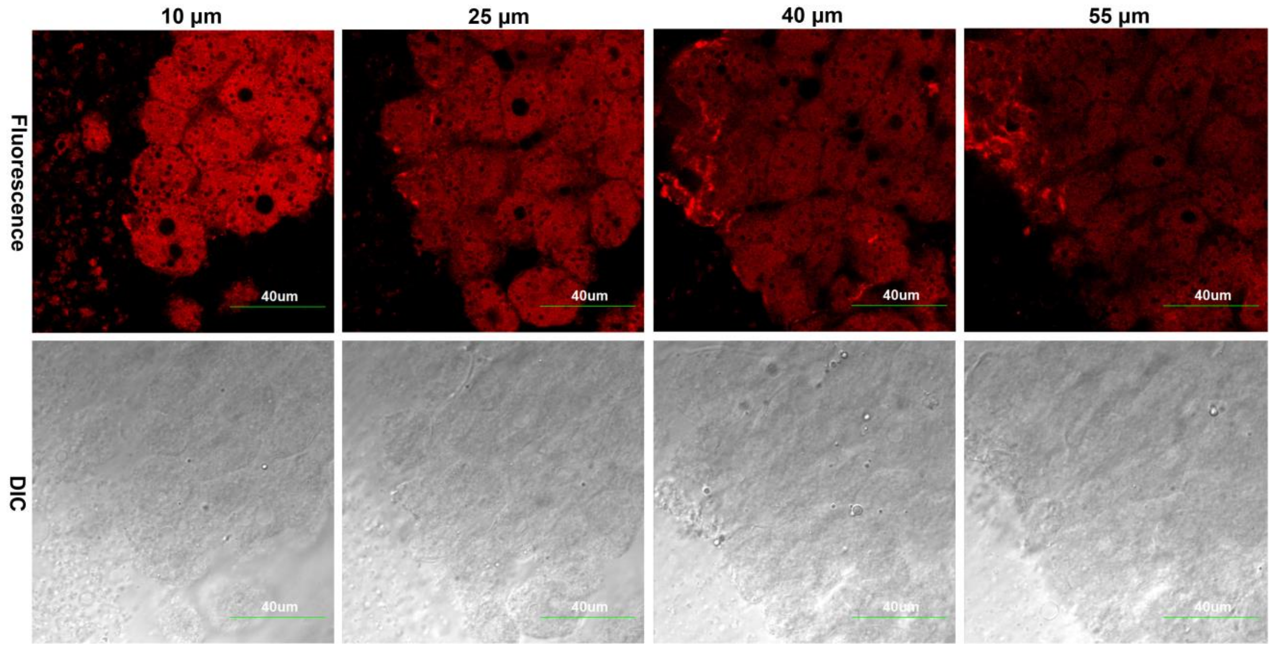

Figure S23. Fluorescence images of mice liver tissue sections incubated with 20 μM **HCy2** in HEPES buffer (20 mM, 0.5% DMSO, 5% glucose, pH 7.4) for 1 h at different depths with a 100× objective. λ_{ex} = 633 nm. λ_{em} = 680−780 nm. Scale bar = 40 μm.

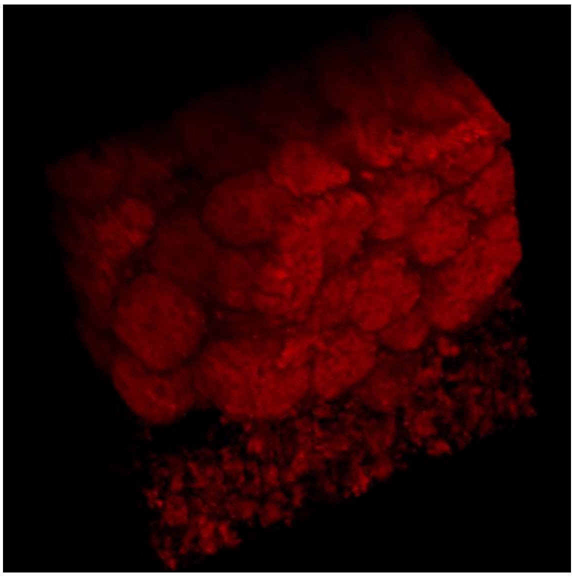

Figure S24. 3D reconstruction of the fluorescence images of mice liver tissue sections.

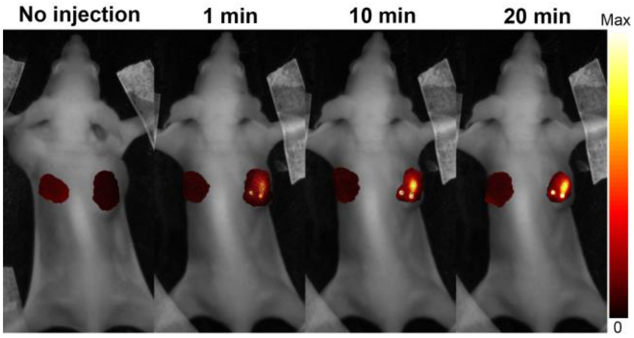

Figure S25. *In vivo* serial whole-body imaging of GST expression with **HCy9** in a nude mouse bearing HepG2 tumors. Into the right tumor 100 μL 20 μM **HCy9** in HEPES buffer (20 mM, 0.5% DMSO, pH 7.4) was injected whereas into the left tumor the HEPES buffer alone was injected. λ_{ex} = 661 nm. λ_{em} = 700−800 nm.

Table S1. Electron affinity *A*, Electronic chemical potential *μ*, chemical hardness *η*, global electrophilicity *ω*, the electrophilic Parr function P_k^+ of the α-carbon and the local electrophilicity ω_k of the α-carbon for probes[a]

	A (**NI-**)[b]	*A* (**HCy-**)	*μ* (**HCy-**)	*η* (**HCy-**)	*ω* (**HCy-**)	P_k^+ (**HCy-**)	ω_k (**HCy-**)	ω_k (**NI-**)[b]
**7**	1.413	4.451	-6.928	4.954	9.689	0.081	0.786	0.290
**6**	1.401	4.462	-6.940	4.955	9.720	0.085	0.825	0.304
**9**	1.475	4.486	-6.965	4.958	9.785	0.092	0.896	0.331
**8**	1.592	4.620	-7.105	4.970	10.156	0.102	1.040	0.369
**10**	1.757	4.576	-7.056	4.959	10.040	0.110	1.106	0.414
**2**	1.744	4.568	-7.051	4.966	10.011	0.135	1.349	0.517
**4**	1.758	4.525	-7.001	4.951	9.899	0.179	1.776	0.680
**3**	1.975	4.612	-7.095	4.965	10.137	0.192	1.947	0.785
**5**	2.021	4.558	-7.038	4.961	9.985	0.190	1.899	0.800
**1**	2.209	4.623	-7.107	4.969	10.167	0.206	2.092	0.909

[a] The list was sorted by ω_k from the lowest to the highest; apart from P_k^+, the unit of all the other parameters is eV. [b] The data were drawn from the reference.[2]

Table S2. Octanol-water partition coefficients (log *P*) for probes **HCy2−4** and **NI2−4** calculated with XLOGP3 program[20] and comparisons of hydrophilicity hereby

	log *P* (**NI-**)	log *P* (**HCy-**)	R_2[a]	R_3[b]
**3**	3.38	2.26	1.50	3.04
**4**	3.84	2.72	1.41	3.05
**2**	5.21	4.09	1.27	3.06

[a] R_2 = log *P* (**NI-**)/log *P* (**HCy-**). [b] $R_3 = x_w$ (**HCy-**)/x_w (**NI-**) = (1 + *P* (**NI-**))/(1 + *P* (**HCy-**)), and refer to Deduction II in this Supporting Information for more about the parameter x_w. The list was sorted by the values of R_2 or R_3 from the highest to the lowest.

Table S3. Nonenzymatic kinetic parameters k_{nonc}, the local electriphilicity ω_k and octanol-water partition coefficients (log *P*) for **DNs-AcRh**, **DNs-Coum** and **DNs-CV** appeared in the literature[1]

	k_{nonc}[a]	ω_k	log *P*[b]	log *P*[c]
**DNs-AcRh**	0.16 ± 0.01	1.019	4.02	3.49
**DNs-Coum**	0.017 ± 0.001	0.427	2.55	2.33
**DNs-CV**	0.0014 ± 0.0003	0.292	3.23	3.28

[a] Data drawn from that literature[1]. [b] Data calculated with ALOGPS 2.1 program[21-23]. [c] Data calculated with XLOGP3 program[20].

Table S4. Catalytic constants k_{cat} for enzymatic reactions of **DNs-AcRh**, **DNs-Coum** and **DNs-CV** reported in the literature[1] with GSH catalyzed by GST isoenzymes[a]

	GSTA1-1	GSTM1-1	GSTP1-1
**DNs-AcRh**	3.1 ± 0.1	1.2 ± 0.1	1.1 ± 0.1
**DNs-Coum**	1.0 ± 0.1	0.47 ± 0.05	0.43 ± 0.06
**DNs-CV**	0.60 ± 0.03	0.23 ± 0.02	0.25 ± 0.02

[a] The unit for k_{nonc} is s^{-1}. These data were drawn from that literature[1].

Table S5. Kinetic parameters for enzymatic reactions of probes with GSH catalyzed by GSTA1-1[a]

	k_{cat} (s^{-1})	K_m (μM)	k_{cat}/K_m (s^{-1} $μM^{-1}$)
**HCy1**	N.D.	N.D.	N.D.
**HCy2**	0.408 ± 0.023	1.2 ± 0.3	0.349 ± 0.064
**HCy3**	N.D.	N.D.	N.D.
**HCy4**	N.D.	N.D.	N.D.
**HCy5**	N.D.	N.D.	N.D.
**HCy6**	0.019 ± 0.001	1.2 ± 0.3	0.016 ± 0.003
**HCy7**	0.008 ± 0.000	1.0 ± 0.2	0.008 ± 0.001
**HCy8**	UD	UD	UD
**HCy9**	0.099 ± 0.006	0.8 ± 0.2	0.124 ± 0.021
**HCy10**	0.003 ± 0.000	1.3 ± 0.1	0.002 ± 0.000

[a] N.D. = Not determined, which means the data are unavailable due to too large nonenzymatic background noise and/or too high reaction rate hindering the data acquisition ahead of the reaction completeness. UD = undetectable, which means the data are unavailable due to the low sensitivity of corresponding probes to this isoenzyme.

Table S6. Kinetic parameters for enzymatic reactions of probes with GSH catalyzed by GSTM1-1[a]

	k_{cat} (s^{-1})	K_m (μM)	k_{cat}/K_m ($s^{-1}μM^{-1}$)
**HCy1**	N.D.	N.D.	N.D.
**HCy2**	0.379 ± 0.035	1.9 ± 0.6	0.204 ± 0.050
**HCy3**	N.D.	N.D.	N.D.
**HCy4**	N.D.	N.D.	N.D.
**HCy5**	N.D.	N.D.	N.D.
**HCy6**	0.016 ± 0.002	1.6 ± 0.6	0.010 ± 0.002
**HCy7**	UD	UD	UD
**HCy8**	0.009 ± 0.000	1.8 ± 0.4	0.005 ± 0.001
**HCy9**	0.006 ± 0.001	0.6 ± 0.2	0.010 ± 0.002
**HCy10**	0.009 ± 0.001	3.3 ± 1.3	0.003 ± 0.001

[a] N.D. = Not determined, which means the data are unavailable due to too large nonenzymatic background noise and/or too high reaction rate hindering the data acquisition ahead of the reaction completeness. UD = undetectable, which means the data are unavailable due to the low sensitivity of corresponding probes to this isoenzyme.

Table S7. Kinetic parameters for enzymatic reactions of probes with GSH catalyzed by GSTP1-1[a]

	k_{cat} (s^{-1})	K_m (μM)	k_{cat}/K_m ($s^{-1}μM^{-1}$)
**HCy1**	N.D.	N.D.	N.D.
**HCy2**	0.392 ± 0.041	2.0 ± 0.6	0.199 ± 0.045
**HCy3**	N.D.	N.D.	N.D.
**HCy4**	N.D.	N.D.	N.D.
**HCy5**	N.D.	N.D.	N.D.
**HCy6**	UD	UD	UD
**HCy7**	UD	UD	UD
**HCy8**	UD	UD	UD
**HCy9**	UD	UD	UD
**HCy10**	UD	UD	UD

[a] N.D. = Not determined, which means the data are unavailable due to too large nonenzymatic background noise and/or too high reaction rate hindering the data acquisition ahead of the reaction completeness. UD = undetectable, which means the data are unavailable due to the low sensitivity of corresponding probes to this isoenzyme.

Table S8. Calculated electronic transitions of **HCy9**, **HCy** and **HCy10** at TD-DFT/B3LYP/aug-cc-pVDZ level with the SMD solvation model (solvent: DMSO)

Transitions	λ_{cal} (nm)	λ_{exp} (nm)	f	CI expansion coefficients
**HCy9**				
$S_0 \rightarrow S_1$	592		0.007	0.706 (H→L)
$S_0 \rightarrow S_2$	548	580	1.274	0.707 (H→L+1)
**HCy**				
$S_0 \rightarrow S_1$	596	680	1.124	0.707 (H→L)
$S_0 \rightarrow S_2$	436	550	0.107	0.682 (H-1→L)
$S_0 \rightarrow S_3$	404		0.000	0.694 (H-2→L)
**HCy10**				
$S_0 \rightarrow S_1$	628		0.032	0.706 (H→L)
$S_0 \rightarrow S_2$	547	580	1.240	0.707 (H→L+1)

NMR Spectra Data

ZXX.fid

^{1}H NMR (400 MHz, DMSO-d_6) δ 8.27 (d, J = 14.1 Hz, 2H), 7.67 – 7.63 (m, 2H), 7.49 – 7.42 (m, 4H), 7.33 – 7.27 (m, 2H), 6.34 (d, J = 14.2 Hz, 2H), 4.27 (q, J = 7.1 Hz, 4H), 2.73 (t, J = 6.1 Hz, 4H), 1.90 – 1.82 (m, 2H), 1.68 (s, 12H), 1.32 (t, J = 7.1 Hz, 6H).

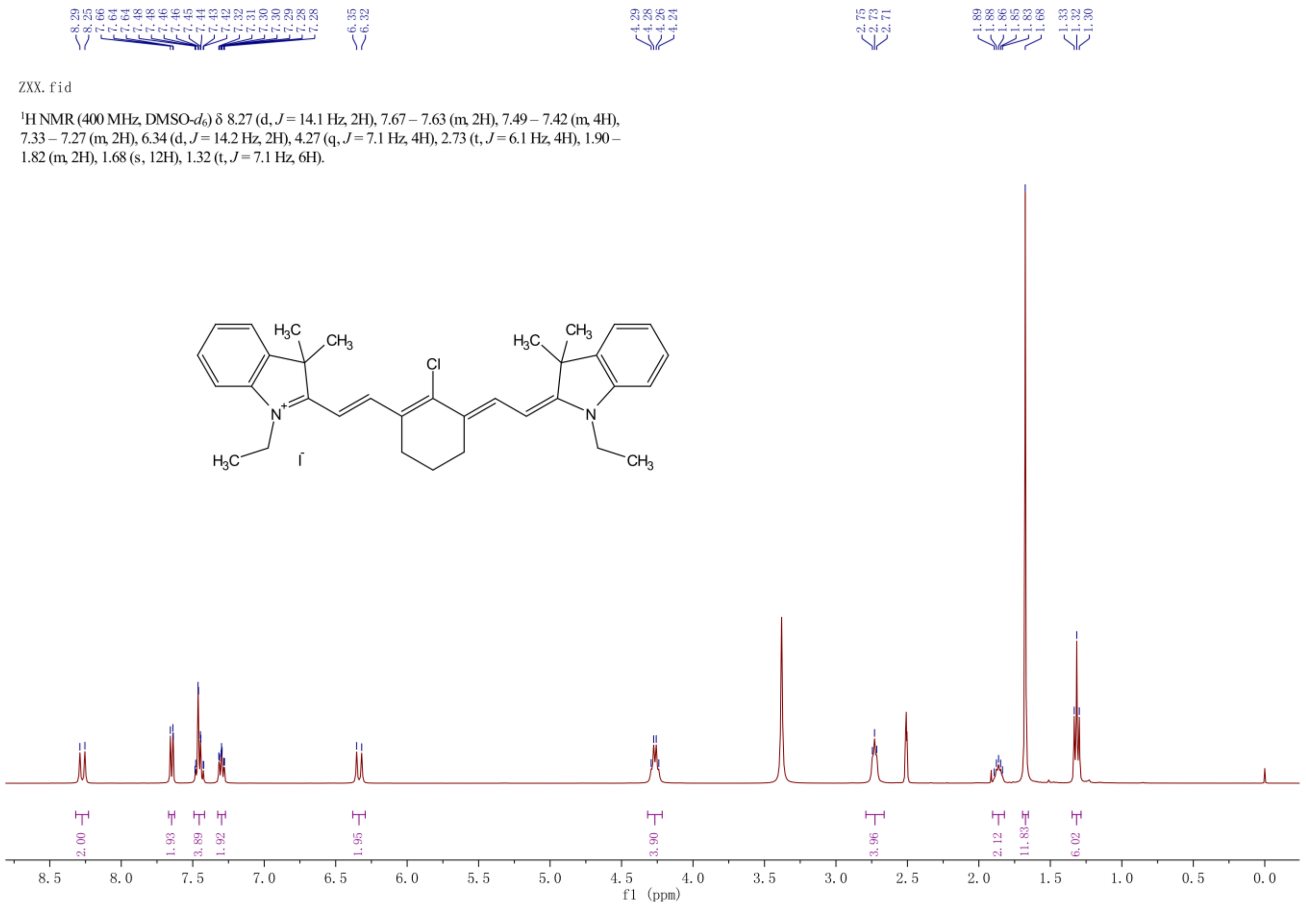

ZXX.fid

^{13}C NMR (101 MHz, DMSO) δ 172.25, 148.47, 143.56, 142.11, 141.66, 129.14, 126.57, 125.65, 123.05, 111.79, 101.77, 49.48, 39.50, 27.87, 26.37, 20.86, 12.72.

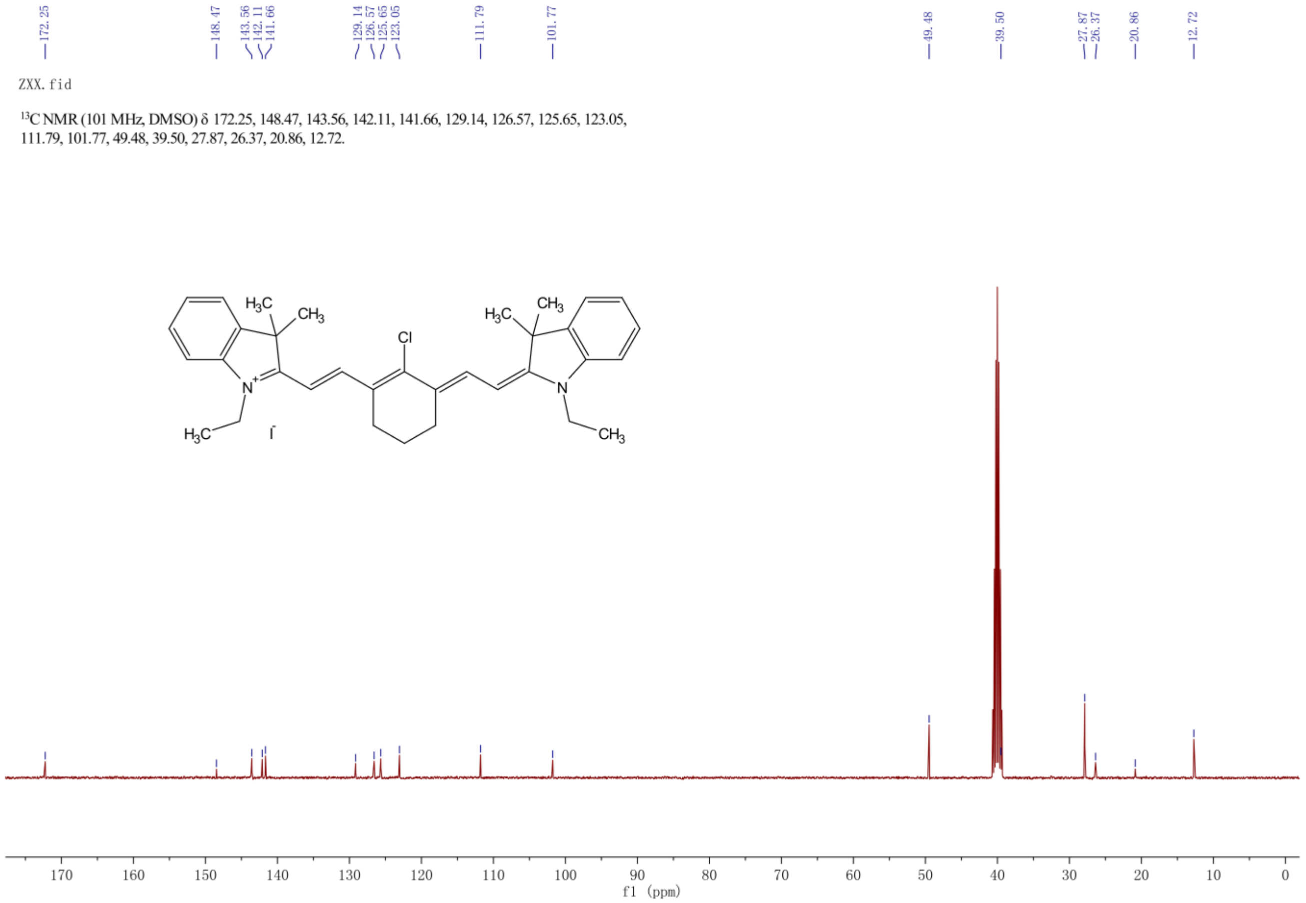

ZXX. fid

^{1}H NMR (400 MHz, Chloroform-*d*) δ 8.14 (d, *J* = 13.5 Hz, 1H), 7.36 – 7.28 (m, 3H), 7.23 (d, *J* = 9.0 Hz, 1H), 7.08 (t, *J* = 7.4 Hz, 1H), 6.88 (d, *J* = 8.0 Hz, 1H), 6.81 (dd, *J* = 9.0, 2.0 Hz, 1H), 6.63 (t, *J* = 1.3 Hz, 1H), 5.69 (d, *J* = 13.5 Hz, 1H), 3.91 (q, *J* = 7.2 Hz, 2H), 2.69 (t, *J* = 6.0 Hz, 2H), 2.63 (t, *J* = 6.1 Hz, 2H), 1.90 (p, *J* = 6.2 Hz, 2H), 1.67 (s, 6H), 1.37 (t, *J* = 7.2 Hz, 3H).

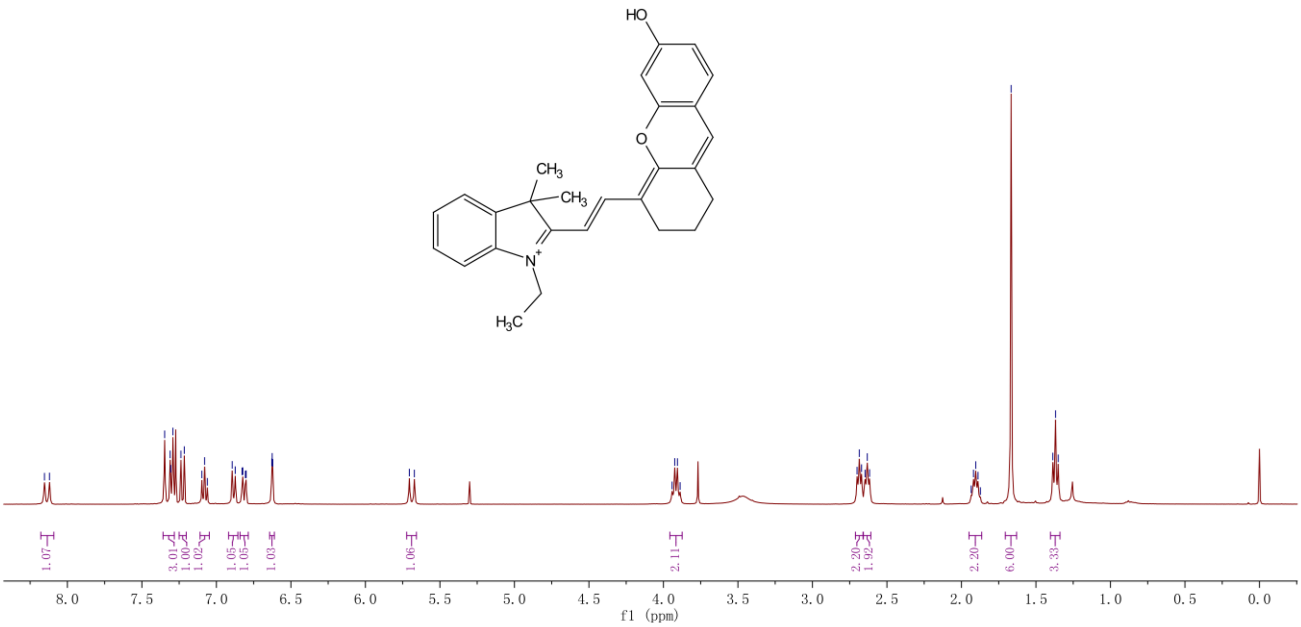

ZXX. fid

^{13}C NMR (101 MHz, $CDCl_3$) δ 165.92, 160.47, 158.72, 142.79, 140.01, 139.89, 133.86, 129.72, 128.25, 126.35, 122.67, 122.15, 117.27, 116.21, 115.83, 108.16, 103.50, 94.57, 77.37, 77.05, 76.73, 47.77, 38.25, 28.57, 28.06, 24.52, 21.26, 11.66.

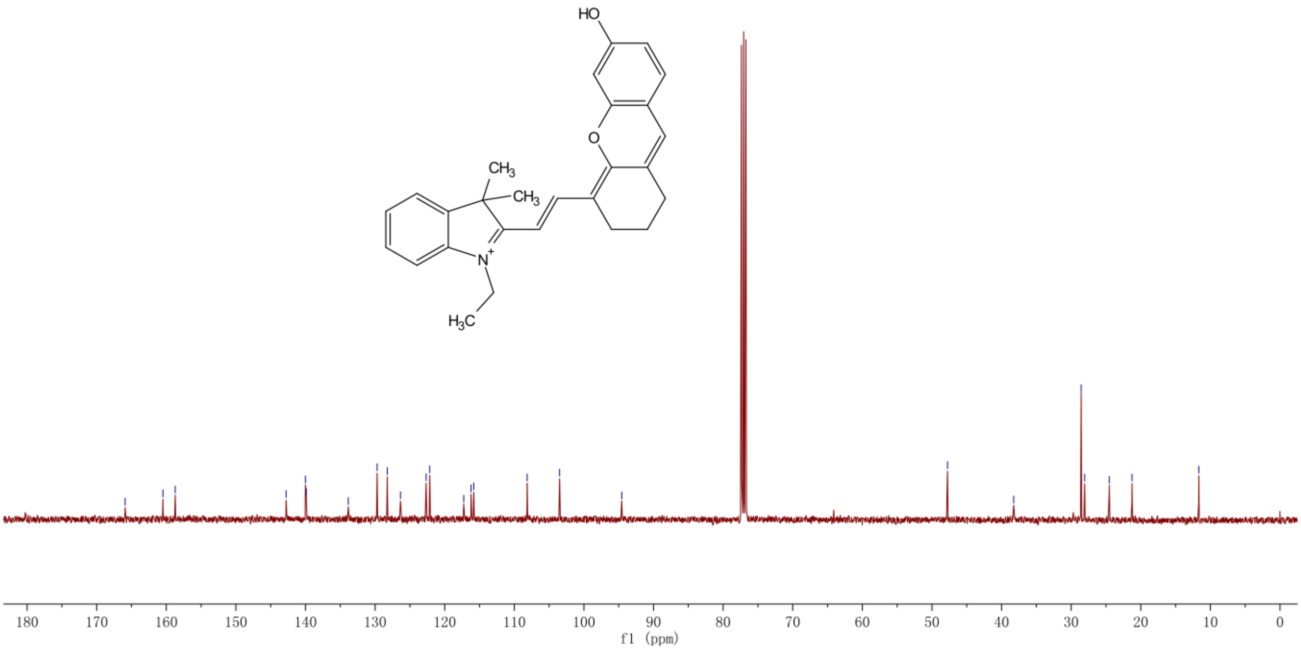

ZXX.fid

^{1}H NMR (400 MHz, Chloroform-*d*) δ 8.68 (d, *J* = 1.8 Hz, 1H), 8.65 – 8.58 (m, 2H), 8.39 (d, *J* = 8.6 Hz, 1H), 8.36 (d, *J* = 8.6 Hz, 1H), 8.28 (dd, *J* = 8.5, 2.2 Hz, 1H), 8.25 (d, *J* = 2.2 Hz, 1H), 7.54 (t, *J* = 7.6 Hz, 2H), 7.48 (dd, *J* = 7.4, 5.6 Hz, 2H), 7.34 (d, *J* = 8.4 Hz, 1H), 7.21 (d, *J* = 2.2 Hz, 1H), 7.07 – 6.96 (m, 2H), 6.73 (d, *J* = 15.1 Hz, 1H), 4.59 (d, *J* = 7.5 Hz, 2H), 2.78 (d, *J* = 6.2 Hz, 2H), 2.71 (t, *J* = 6.0 Hz, 2H), 1.92 (s, 2H), 1.81 (s, 6H), 1.56 (t, *J* = 6.8 Hz, 3H).

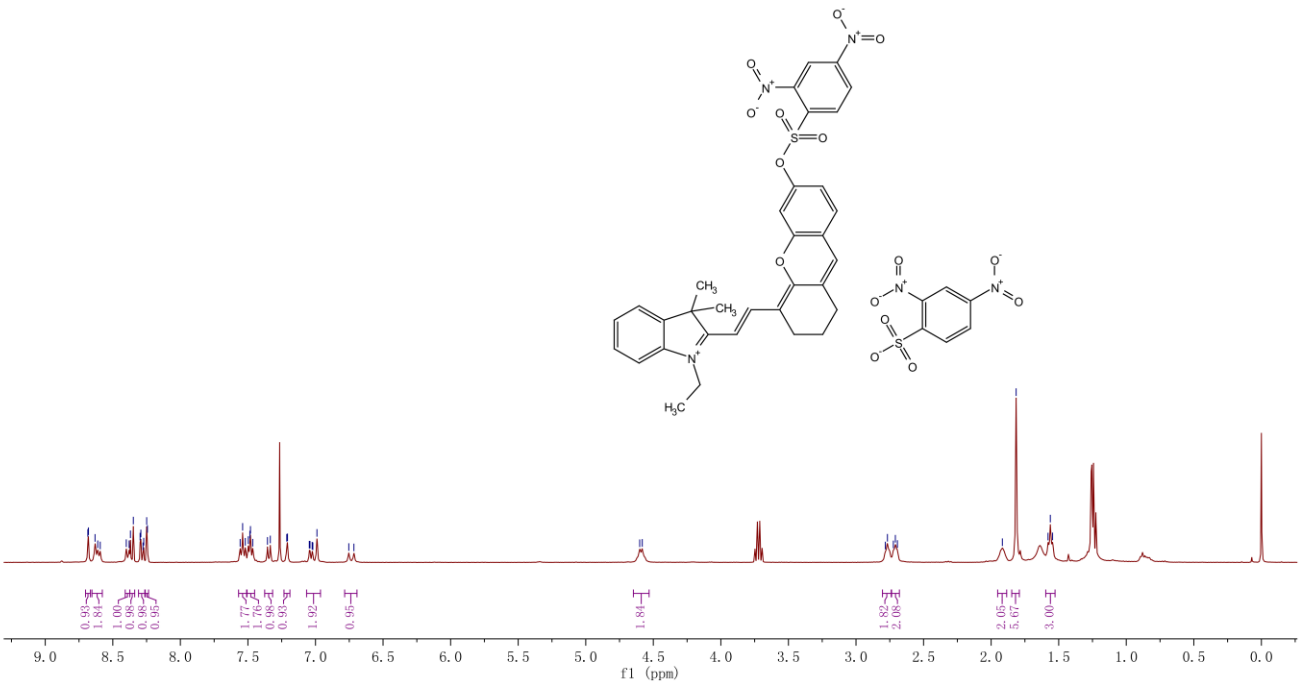

ZXX.fid

^{13}C NMR (101 MHz, $CDCl_3$) δ 178.69, 173.14, 158.51, 152.98, 151.14, 149.39, 148.78, 148.24, 147.46, 146.57, 145.37, 142.50, 140.84, 134.28, 133.16, 131.89, 131.78, 130.89, 129.45, 129.15, 128.67, 128.37, 127.16, 125.29, 122.65, 121.71, 120.48, 118.52, 118.24, 115.97, 113.37, 110.12, 107.17, 51.34, 41.81, 29.58, 27.80, 24.16, 20.08, 13.21.

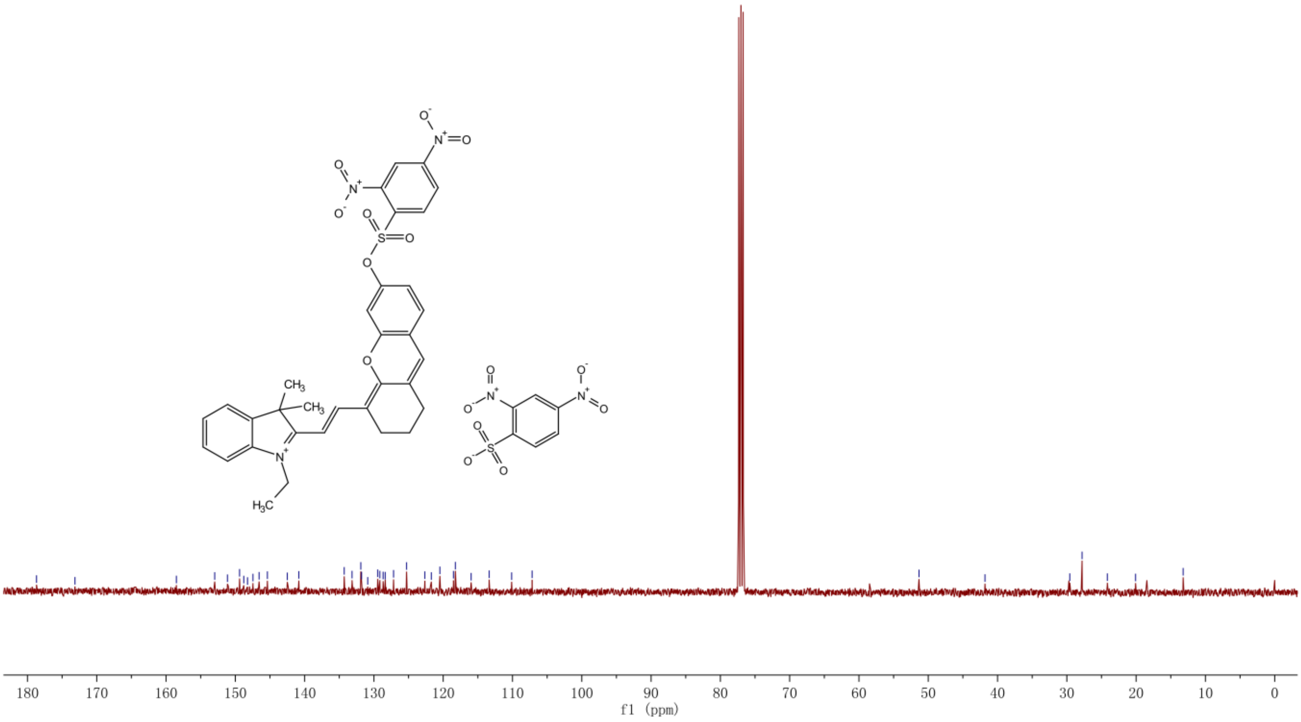

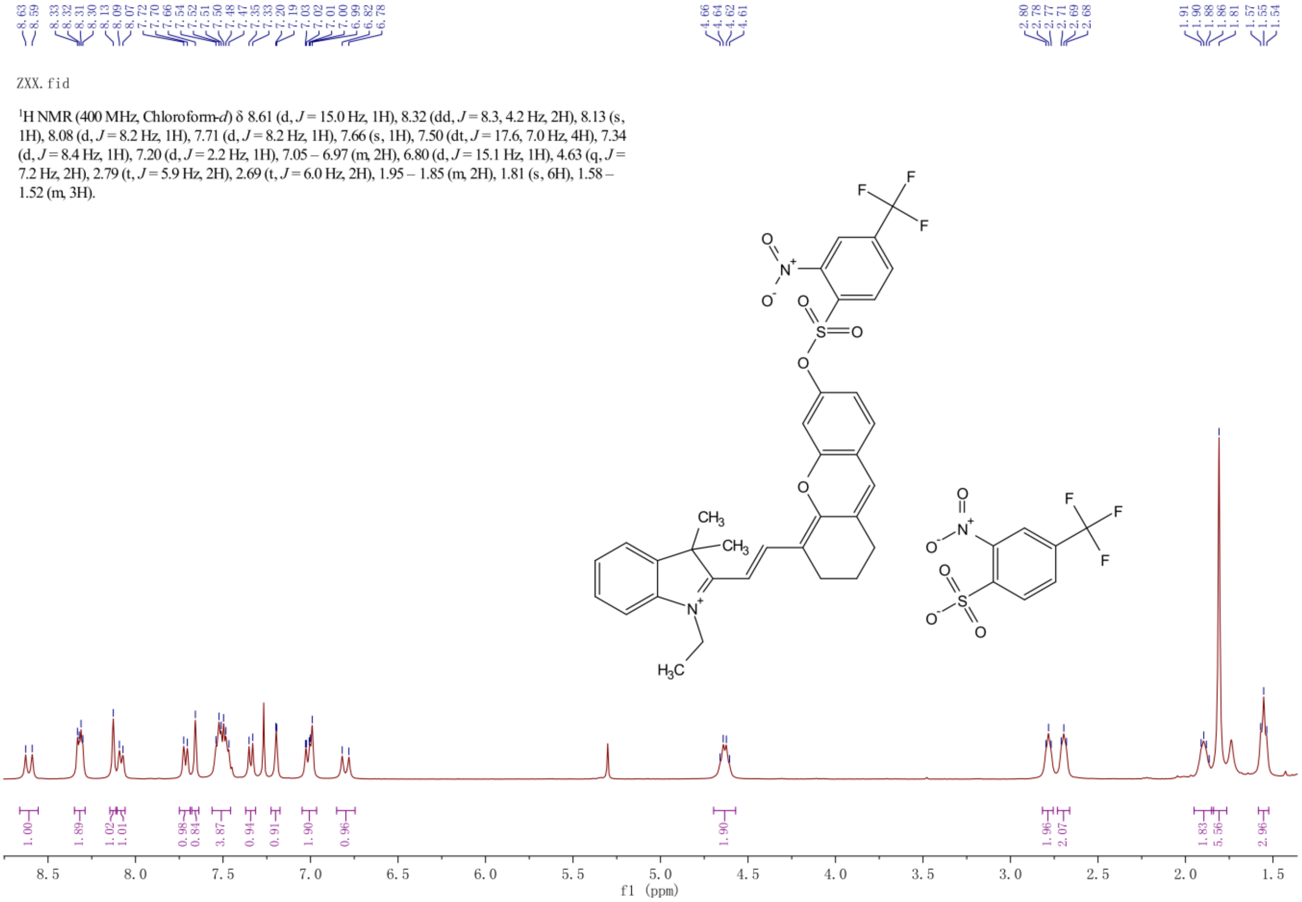

^{1}H NMR (400 MHz, Chloroform-*d*) δ 8.61 (d, *J* = 15.0 Hz, 1H), 8.32 (dd, *J* = 8.3, 4.2 Hz, 2H), 8.13 (s, 1H), 8.08 (d, *J* = 8.2 Hz, 1H), 7.71 (d, *J* = 8.2 Hz, 1H), 7.66 (s, 1H), 7.50 (dt, *J* = 17.6, 7.0 Hz, 4H), 7.34 (d, *J* = 8.4 Hz, 1H), 7.20 (d, *J* = 2.2 Hz, 1H), 7.05 – 6.97 (m, 2H), 6.80 (d, *J* = 15.1 Hz, 1H), 4.63 (q, *J* = 7.2 Hz, 2H), 2.79 (t, *J* = 5.9 Hz, 2H), 2.69 (t, *J* = 6.0 Hz, 2H), 1.95 – 1.85 (m, 2H), 1.81 (s, 6H), 1.58 – 1.52 (m, 3H).

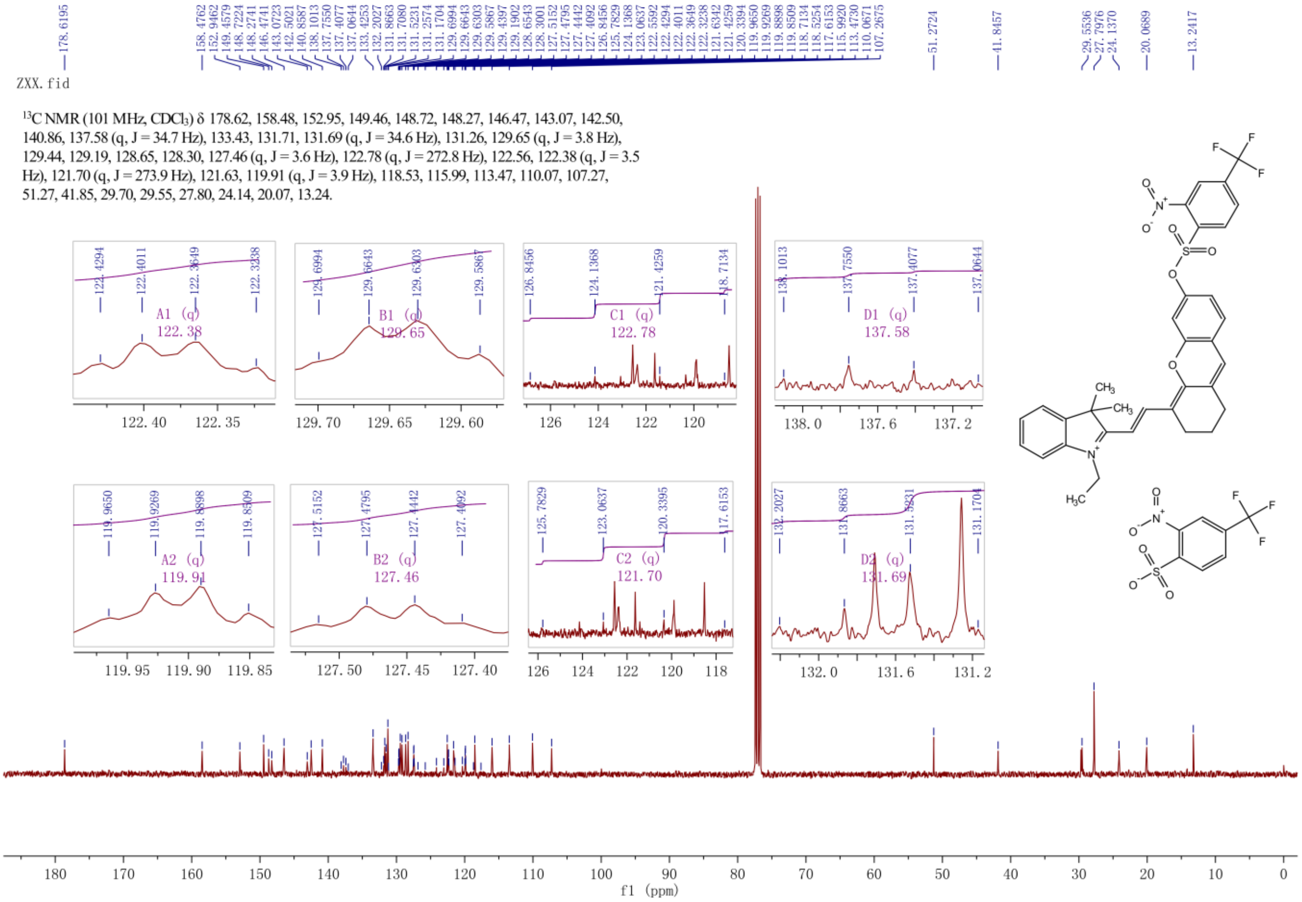

^{13}C NMR (101 MHz, $CDCl_3$) δ 178.62, 158.48, 152.95, 149.46, 148.72, 148.27, 146.47, 143.07, 142.50, 140.86, 137.58 (q, J = 34.7 Hz), 133.43, 131.71, 131.69 (q, J = 34.6 Hz), 131.26, 129.65 (q, J = 3.8 Hz), 129.44, 129.19, 128.65, 128.30, 127.46 (q, J = 3.6 Hz), 122.78 (q, J = 272.8 Hz), 122.56, 122.38 (q, J = 3.5 Hz), 121.70 (q, J = 273.9 Hz), 121.63, 119.91 (q, J = 3.9 Hz), 118.53, 115.99, 113.47, 110.07, 107.27, 51.27, 41.85, 29.70, 29.55, 27.80, 24.14, 20.07, 13.24.

ZXX.fid

^{19}F NMR (376 MHz, Chloroform-*d*) δ -62.82, -63.35.

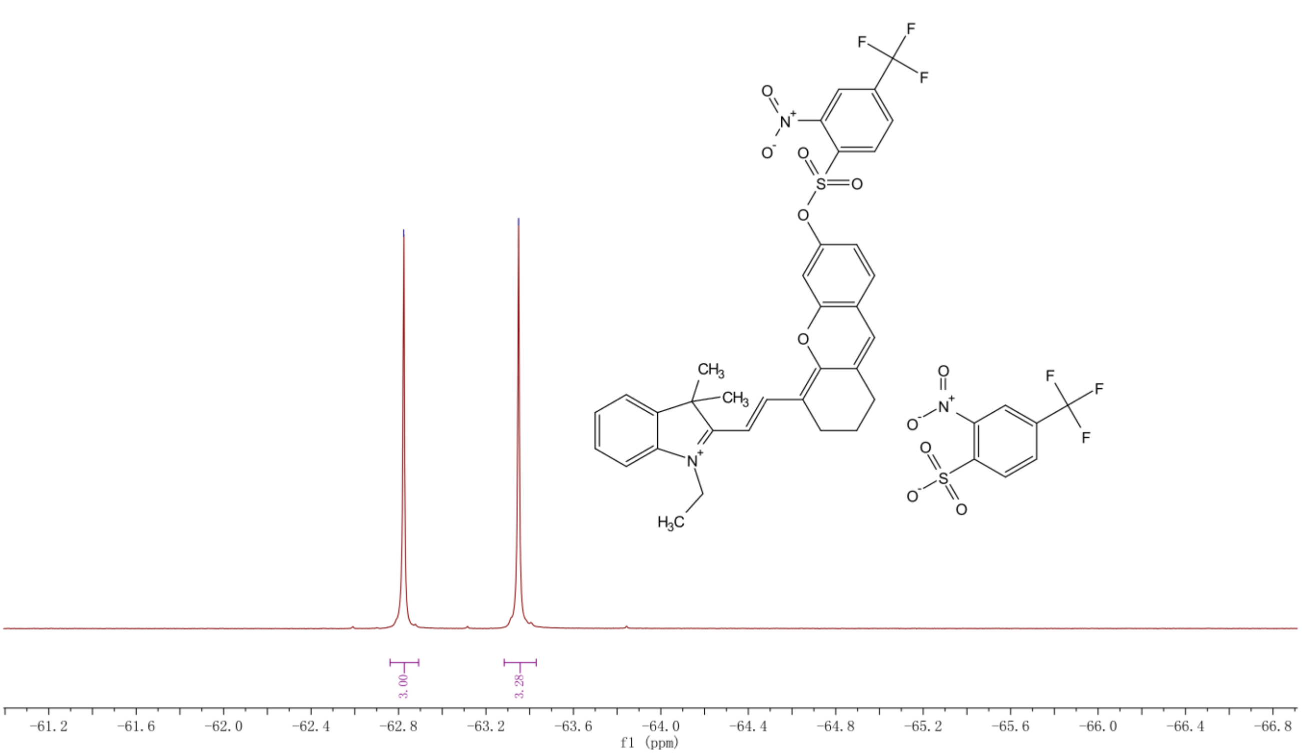

ZXX.fid

^{1}H NMR (400 MHz, Chloroform-*d*) δ 8.61 (d, *J* = 15.2 Hz, 1H), 8.30 (t, *J* = 7.2 Hz, 2H), 8.19 – 8.13 (m, 2H), 7.73 (d, *J* = 8.2 Hz, 1H), 7.67 (s, 1H), 7.60 – 7.44 (m, 4H), 7.37 (d, *J* = 8.4 Hz, 1H), 7.22 – 7.16 (m, 1H), 7.06 – 7.00 (m, 2H), 6.70 (d, *J* = 15.2 Hz, 1H), 4.57 (q, *J* = 7.3 Hz, 2H), 2.75 (t, *J* = 6.0 Hz, 2H), 2.70 (t, *J* = 6.1 Hz, 2H), 1.90 (p, *J* = 6.1 Hz, 2H), 1.81 (s, 6H), 1.55 (t, *J* = 7.2 Hz, 3H).

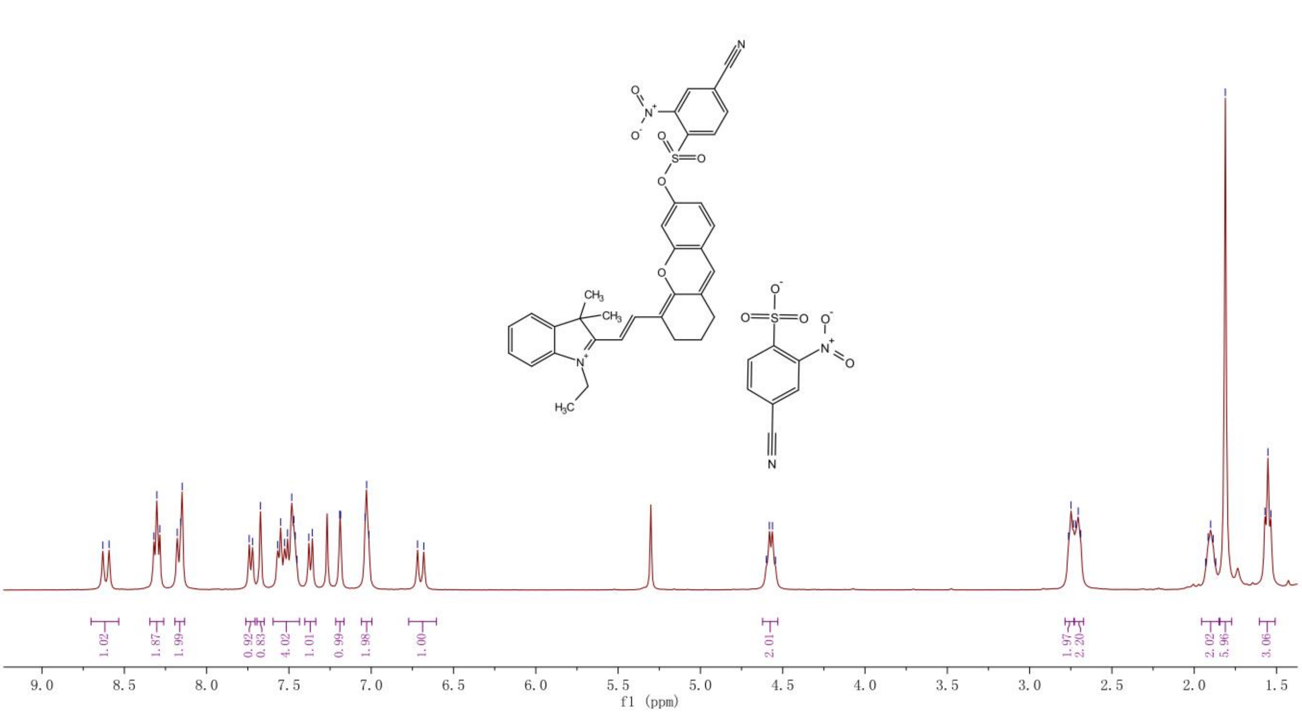

ZXX.fid

^{13}C NMR (101 MHz, $CDCl_3$) δ 178.64, 158.71, 152.94, 149.49, 148.55, 148.38, 146.60, 143.99, 142.48, 140.80, 136.40, 134.15, 133.46, 131.76, 131.62, 131.52, 129.50, 129.45, 128.80, 128.37, 128.24, 126.17, 122.72, 121.66, 119.69, 118.66, 116.64, 115.83, 114.95, 113.33, 110.09, 106.88, 51.35, 41.77, 29.52, 27.81, 24.17, 20.08, 13.19.

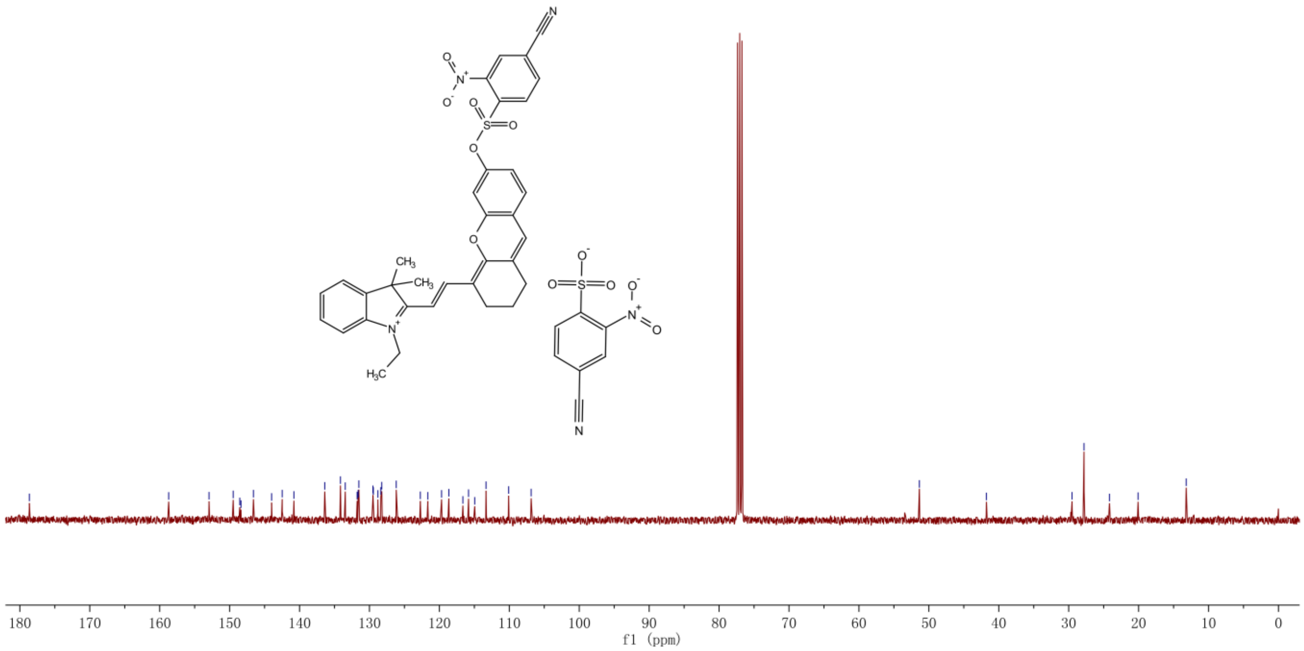

ZXX.fid

^{1}H NMR (400 MHz, Chloroform-*d*) δ 8.61 (d, *J* = 13.2 Hz, 2H), 8.17 (s, 1H), 7.90 (s, 1H), 7.73 (s, 1H), 7.58 – 7.43 (m, 4H), 7.41 (d, *J* = 8.4 Hz, 1H), 7.20 (d, *J* = 2.2 Hz, 1H), 7.11 (dd, *J* = 8.4, 2.2 Hz, 1H), 7.05 (s, 1H), 6.74 (d, *J* = 15.2 Hz, 1H), 4.61 (q, *J* = 7.2 Hz, 2H), 3.11 (s, 6H), 2.98 (s, 6H), 2.78 – 2.68 (m, 4H), 1.90 (p, *J* = 6.0 Hz, 2H), 1.81 (s, 6H), 1.55 (t, *J* = 7.1 Hz, 3H).

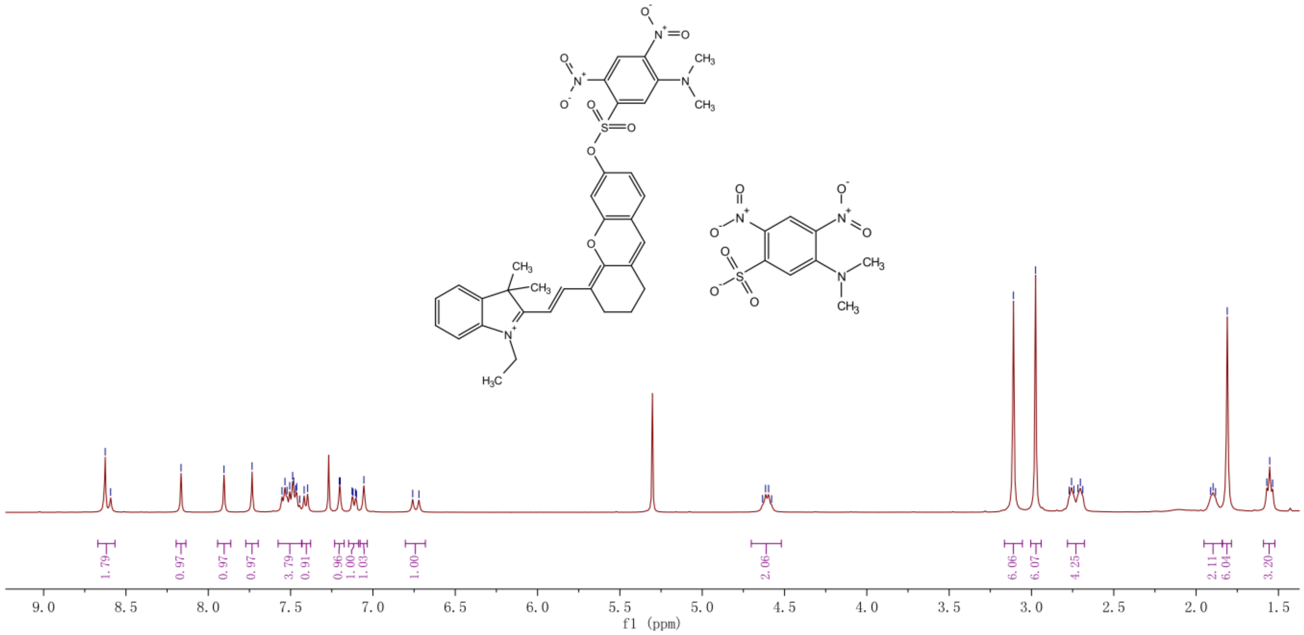

ZXX.fid

^{13}C NMR (101 MHz, $CDCl_3$) δ 178.52, 158.72, 152.95, 149.74, 147.35, 146.74, 146.45, 142.47, 140.87, 136.12, 134.46, 134.12, 133.33, 131.51, 129.60, 129.42, 128.71, 128.25, 127.10, 124.32, 122.59, 121.87, 121.46, 119.52, 118.77, 115.89, 113.38, 110.10, 107.00, 51.25, 42.84, 42.48, 41.77, 29.53, 27.83, 24.12, 20.10, 13.22.

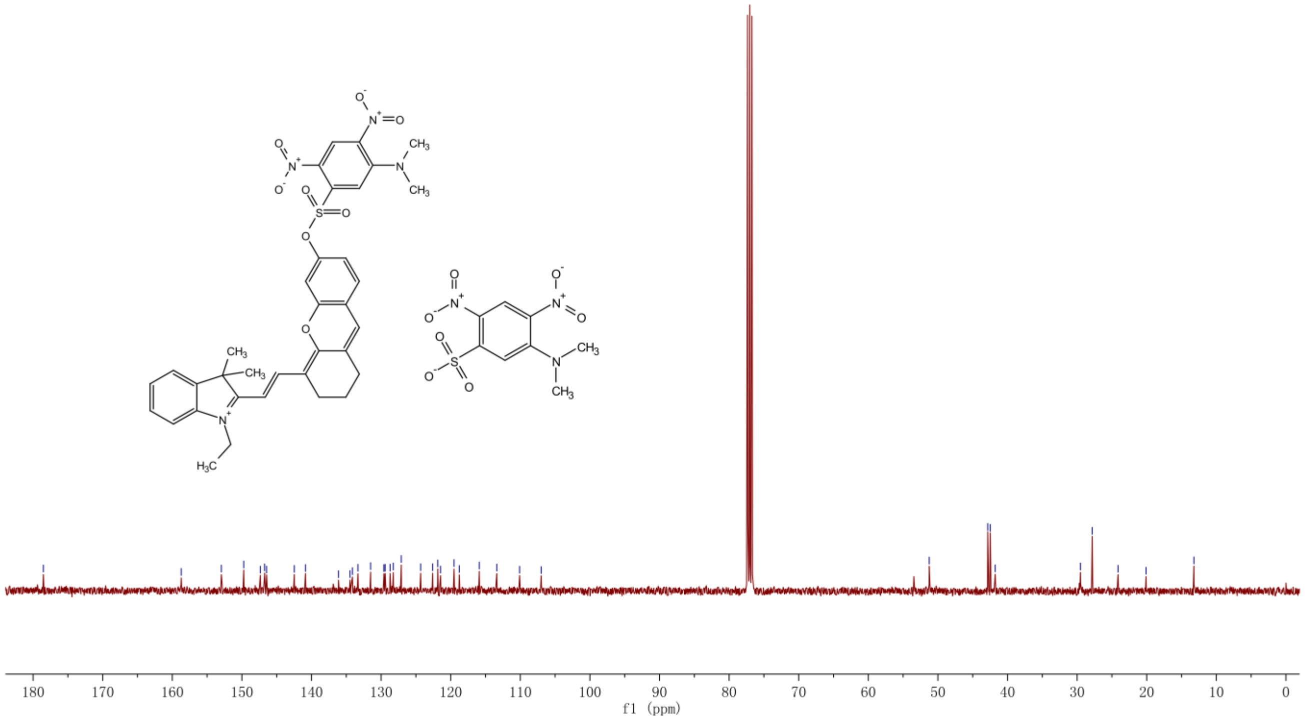

ZXX.fid

^{1}H NMR (400 MHz, Chloroform-*d*) δ 8.62 (d, *J* = 15.3 Hz, 1H), 8.51 (s, 1H), 8.08 (s, 1H), 7.95 (s, 1H), 7.84 (s, 1H), 7.58 – 7.45 (m, 4H), 7.40 (d, *J* = 8.5 Hz, 1H), 7.21 (d, *J* = 2.3 Hz, 1H), 7.09 (dd, *J* = 8.5, 2.3 Hz, 1H), 7.04 (s, 1H), 6.70 (d, *J* = 15.3 Hz, 1H), 4.57 (q, *J* = 7.2 Hz, 2H), 4.13 (s, 3H), 4.03 (s, 3H), 2.80 – 2.67 (m, 4H), 1.91 (p, *J* = 6.3 Hz, 2H), 1.82 (s, 6H), 1.56 (t, *J* = 7.2 Hz, 3H).

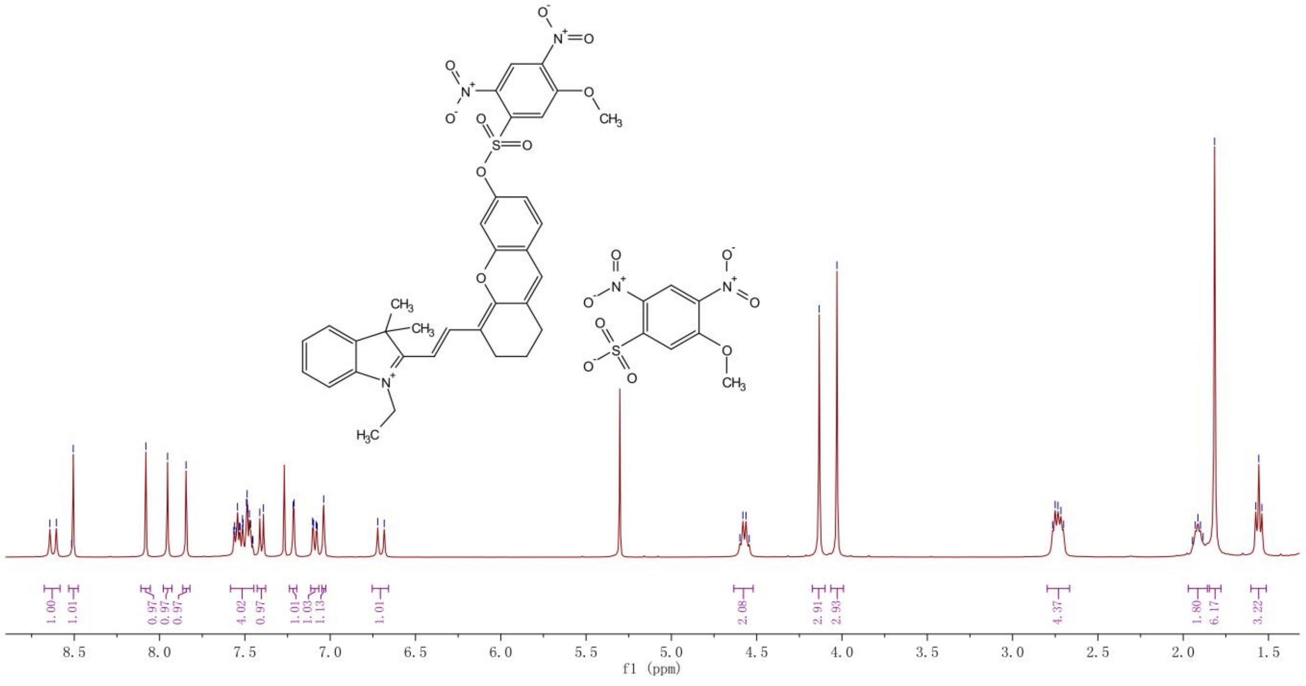

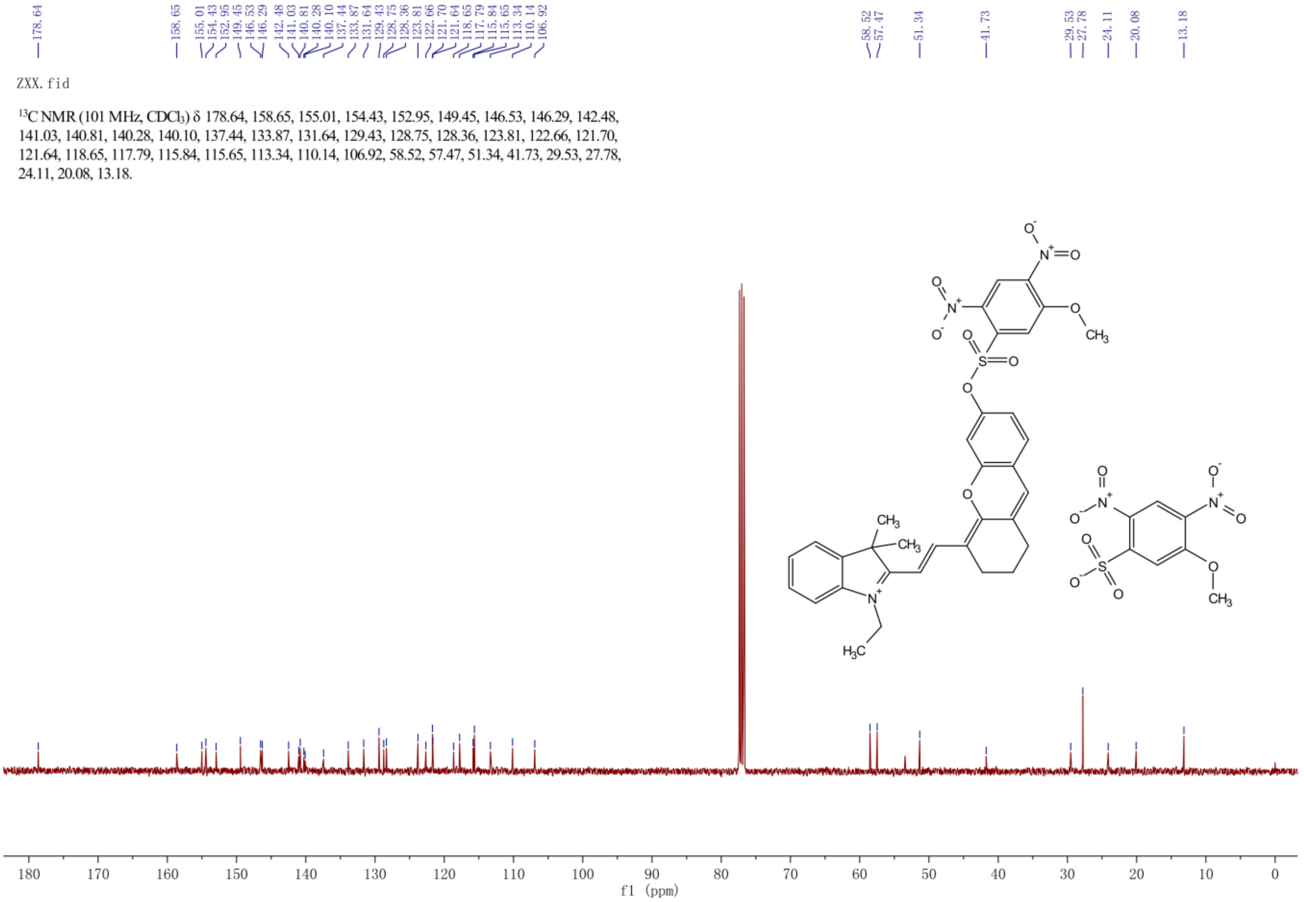

^{13}C NMR (101 MHz, $CDCl_3$) δ 178.64, 158.65, 155.01, 154.43, 152.95, 149.45, 146.53, 146.29, 142.48, 141.03, 140.81, 140.28, 140.10, 137.44, 133.87, 131.64, 129.43, 128.75, 128.36, 123.81, 122.66, 121.70, 121.64, 118.65, 117.79, 115.84, 115.65, 113.34, 110.14, 106.92, 58.52, 57.47, 51.34, 41.73, 29.53, 27.78, 24.11, 20.08, 13.18.

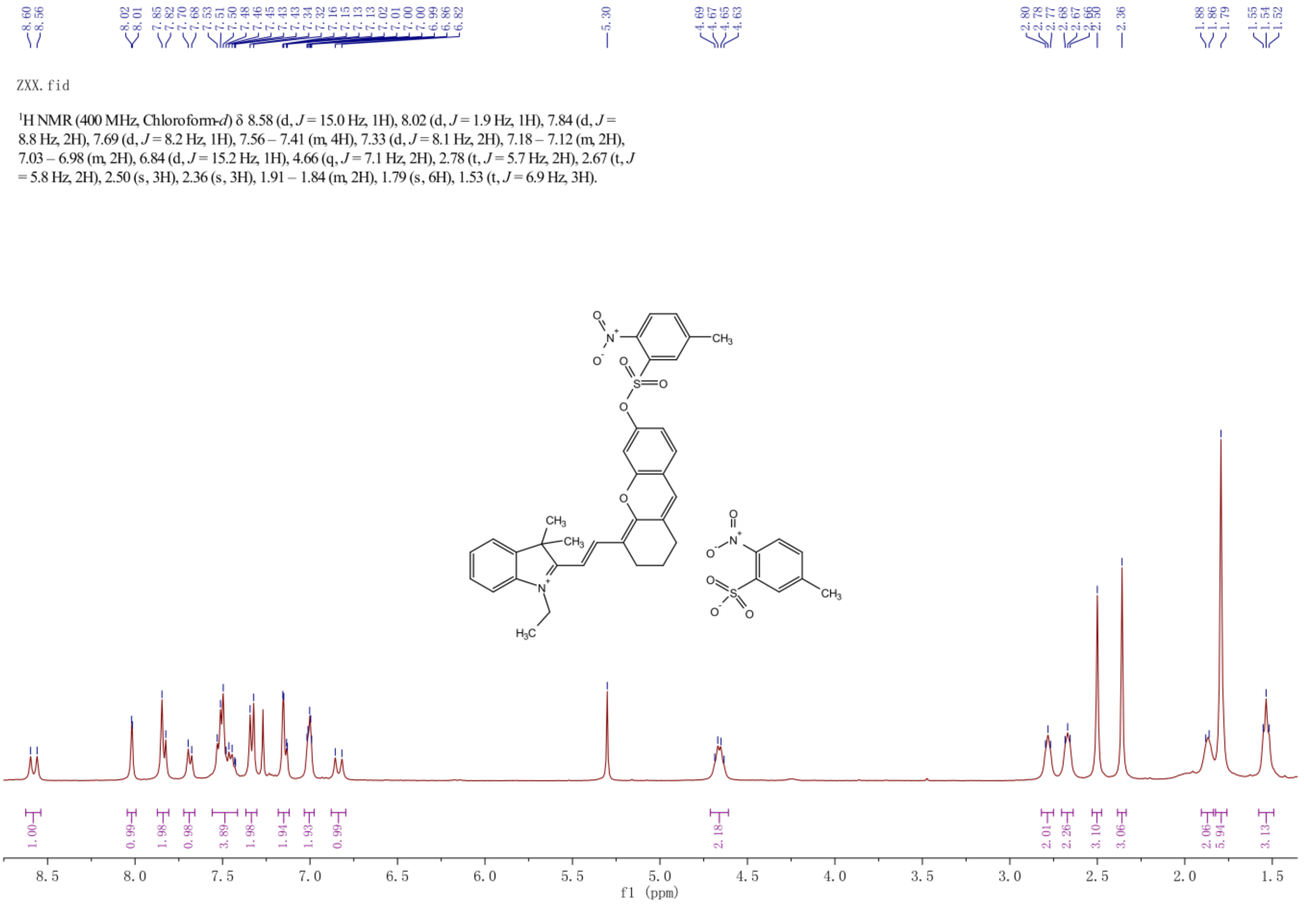

^{1}H NMR (400 MHz, Chloroform-*d*) δ 8.58 (d, *J* = 15.0 Hz, 1H), 8.02 (d, *J* = 1.9 Hz, 1H), 7.84 (d, *J* = 8.8 Hz, 2H), 7.69 (d, *J* = 8.2 Hz, 1H), 7.56 – 7.41 (m, 4H), 7.33 (d, *J* = 8.1 Hz, 2H), 7.18 – 7.12 (m, 2H), 7.03 – 6.98 (m, 2H), 6.84 (d, *J* = 15.2 Hz, 1H), 4.66 (q, *J* = 7.1 Hz, 2H), 2.78 (t, *J* = 5.7 Hz, 2H), 2.67 (t, *J* = 5.8 Hz, 2H), 2.50 (s, 3H), 2.36 (s, 3H), 1.91 – 1.84 (m, 2H), 1.79 (s, 6H), 1.53 (t, *J* = 6.9 Hz, 3H).

ZXX.fid

^{13}C NMR (101 MHz, $CDCl_3$) δ 178.52, 158.40, 152.90, 149.78, 146.35, 144.27, 142.51, 141.59, 140.96, 139.40, 136.35, 132.41, 131.60, 130.56, 129.90, 129.40, 129.17, 128.45, 128.14, 127.89, 125.41, 122.74, 122.47, 121.40, 118.67, 116.14, 113.57, 110.10, 107.56, 51.17, 41.92, 29.57, 27.84, 24.14, 21.48, 21.24, 20.10, 13.30.

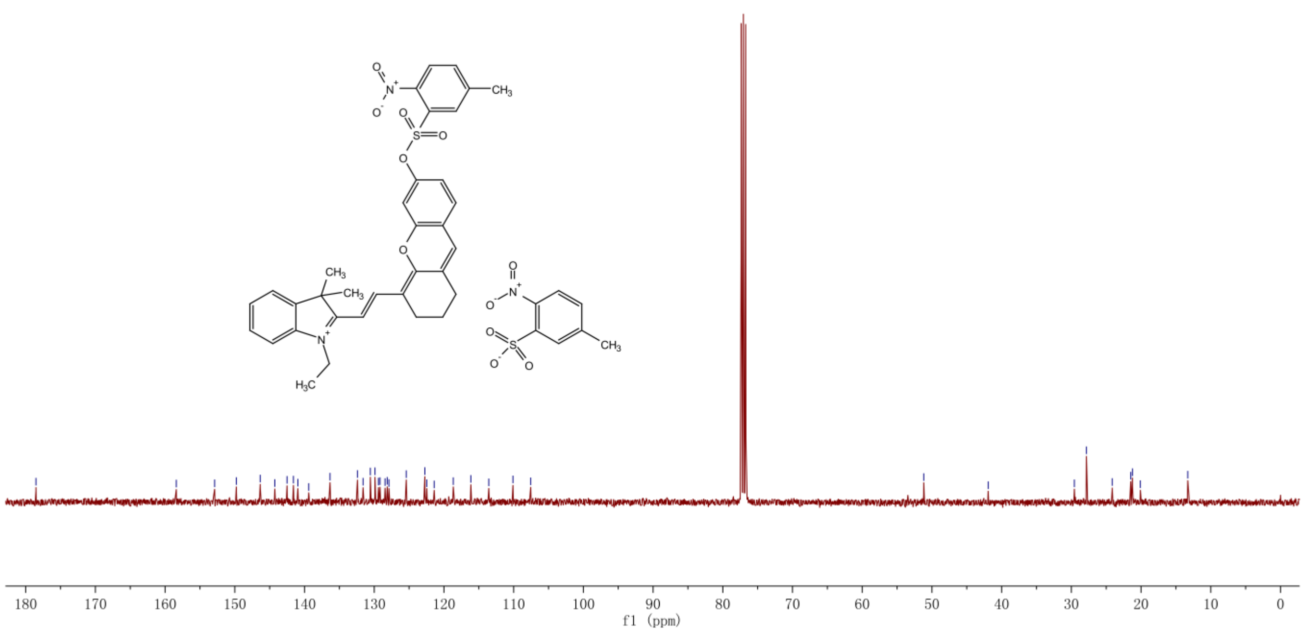

ZXX.fid

^{1}H NMR (400 MHz, Chloroform-*d*) δ 8.58 (d, *J* = 14.7 Hz, 1H), 8.05 (d, *J* = 7.9 Hz, 1H), 7.90 (d, *J* = 7.6 Hz, 1H), 7.66 (s, 1H), 7.50 (tt, *J* = 19.2, 7.4 Hz, 5H), 7.32 (d, *J* = 8.3 Hz, 1H), 7.27 – 7.14 (m, 3H), 7.04 – 6.94 (m, 2H), 6.84 (d, *J* = 14.9 Hz, 1H), 4.72 – 4.62 (m, 2H), 2.79 (t, *J* = 5.8 Hz, 2H), 2.67 (t, *J* = 5.6 Hz, 2H), 2.55 (s, 3H), 2.34 (s, 3H), 1.91 – 1.84 (m, 2H), 1.80 (s, 6H), 1.53 (t, *J* = 6.4 Hz, 3H).

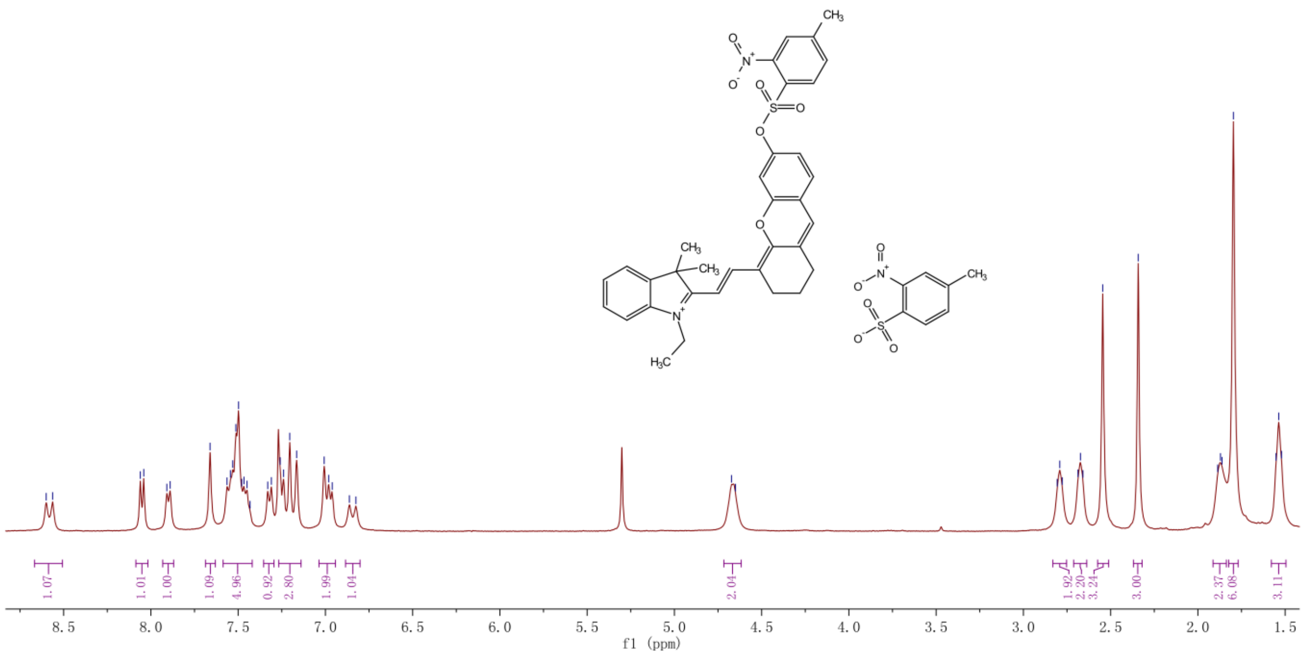

ZXX.fid

^{13}C NMR (101 MHz, $CDCl_3$) δ 178.51, 158.45, 152.89, 149.89, 148.54, 148.32, 146.38, 142.52, 140.96, 140.10, 136.94, 132.95, 132.16, 131.57, 131.29, 130.08, 129.40, 129.26, 128.47, 128.12, 125.50, 124.86, 122.82, 122.47, 121.36, 118.64, 116.15, 113.56, 110.12, 107.57, 51.16, 41.97, 29.59, 27.87, 24.20, 21.69, 20.88, 20.12, 13.32.

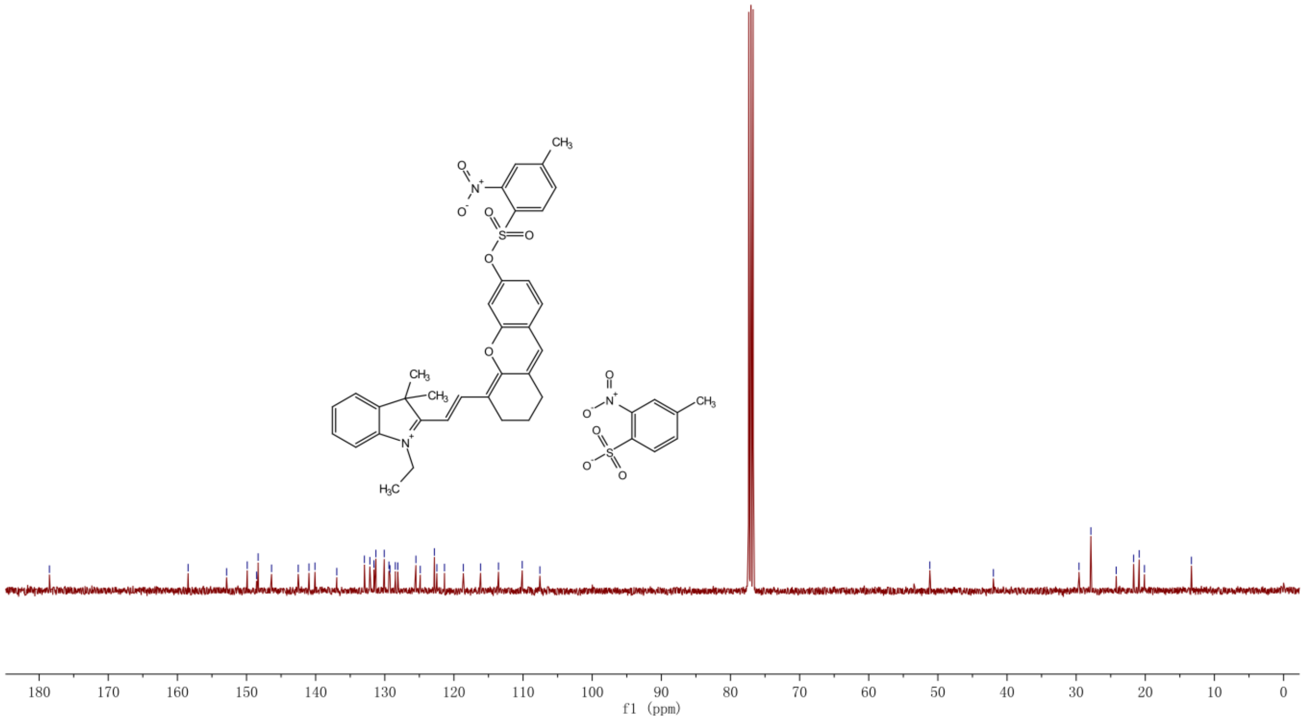

ZXX.fid

^{1}H NMR (400 MHz, Chloroform-*d*) δ 8.56 (d, *J* = 15.2 Hz, 1H), 8.32 (d, *J* = 2.2 Hz, 1H), 8.20 – 8.13 (m, 2H), 8.10 (d, *J* = 8.6 Hz, 1H), 7.96 (d, *J* = 2.3 Hz, 1H), 7.89 (dd, *J* = 8.5, 2.4 Hz, 1H), 7.58 – 7.46 (m, 4H), 7.37 – 7.28 (m, 1H), 7.04 – 6.98 (m, 2H), 6.80 (d, *J* = 15.3 Hz, 1H), 6.71 (dd, *J* = 8.4, 2.3 Hz, 1H), 4.66 (q, *J* = 7.3 Hz, 2H), 2.94 (s, 3H), 2.81 (s, 3H), 2.76 (t, *J* = 6.0 Hz, 2H), 2.67 (t, *J* = 6.2 Hz, 2H), 1.87 – 1.83 (m, 2H), 1.78 (s, 6H), 1.56 (t, *J* = 7.2 Hz, 3H).

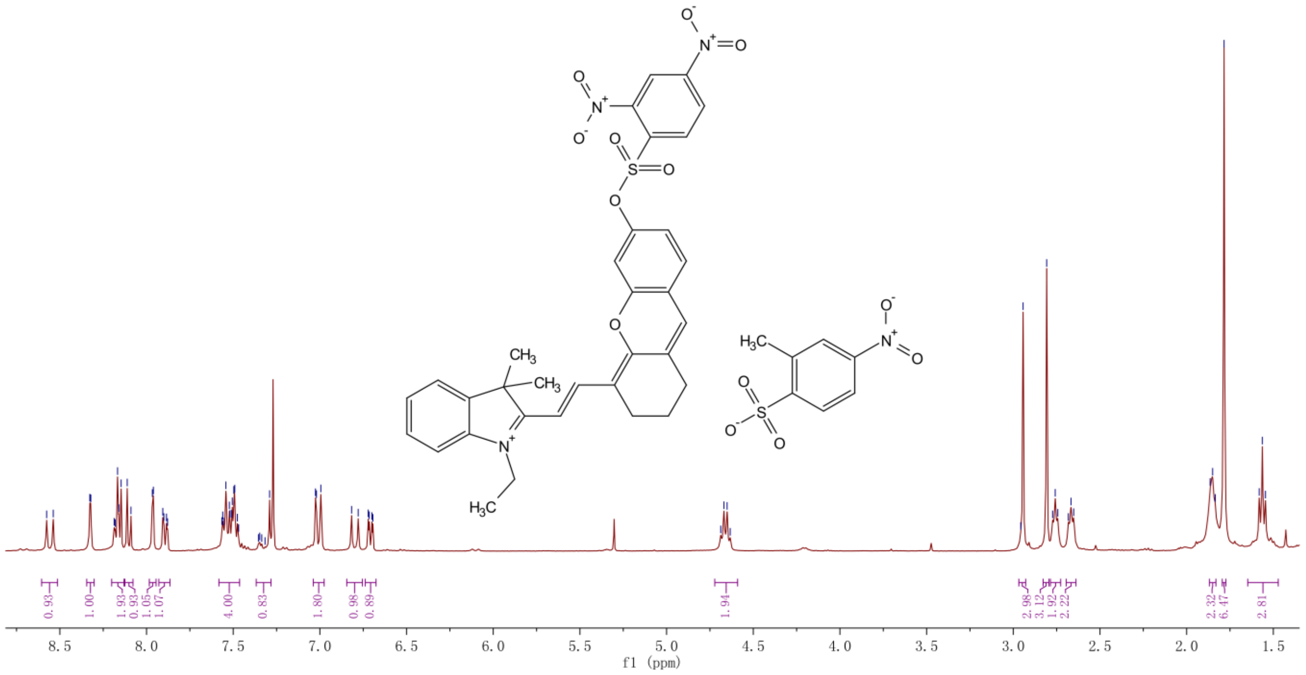

ZXX.fid

^{13}C NMR (101 MHz, $CDCl_3$) δ 178.51, 158.57, 152.88, 151.29, 150.89, 149.95, 147.67, 146.37, 142.36, 141.41, 140.79, 139.50, 138.84, 131.90, 131.44, 129.54, 129.39, 128.63, 128.53, 128.43, 127.44, 125.65, 122.58, 121.33, 121.31, 120.00, 118.32, 115.86, 113.44, 110.05, 107.17, 51.19, 41.85, 29.51, 27.80, 24.15, 20.94, 20.85, 20.03, 13.30.

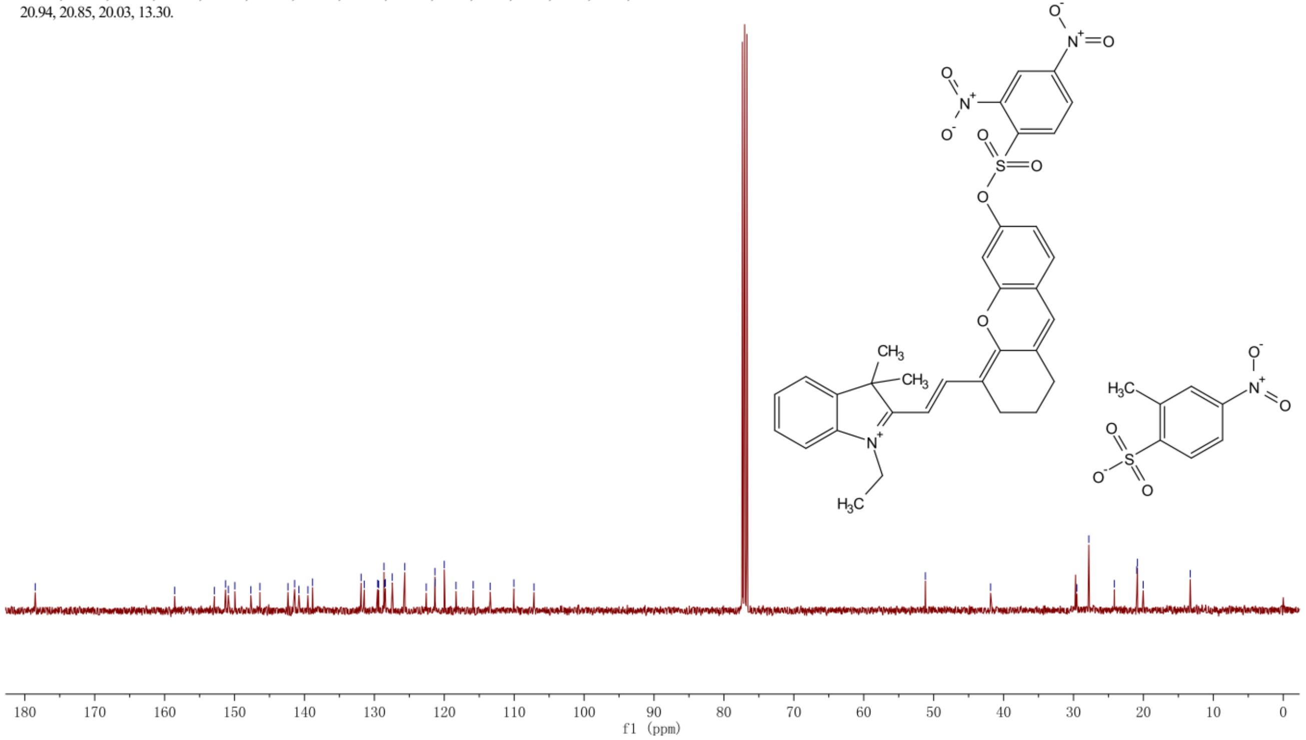

ZXX.fid

^{1}H NMR (400 MHz, Chloroform-*d*) δ 8.57 (d, *J* = 15.2 Hz, 1H), 8.19 (dd, *J* = 7.8, 1.3 Hz, 1H), 8.03 (dd, *J* = 7.9, 1.3 Hz, 1H), 7.95 (td, *J* = 7.7, 1.3 Hz, 1H), 7.89 (dd, *J* = 8.1, 1.3 Hz, 1H), 7.81 (td, *J* = 7.7, 1.4 Hz, 1H), 7.54 (d, *J* = 7.1 Hz, 1H), 7.52 – 7.35 (m, 6H), 7.32 (d, *J* = 8.5 Hz, 1H), 7.13 (d, *J* = 2.2 Hz, 1H), 7.02 – 6.97 (m, 2H), 6.79 (d, *J* = 15.3 Hz, 1H), 4.63 (q, *J* = 7.2 Hz, 2H), 2.76 (t, *J* = 6.0 Hz, 2H), 2.69 – 2.65 (m, 2H), 1.87 (p, *J* = 5.9 Hz, 2H), 1.79 (s, 6H), 1.53 (t, *J* = 7.1 Hz, 3H).

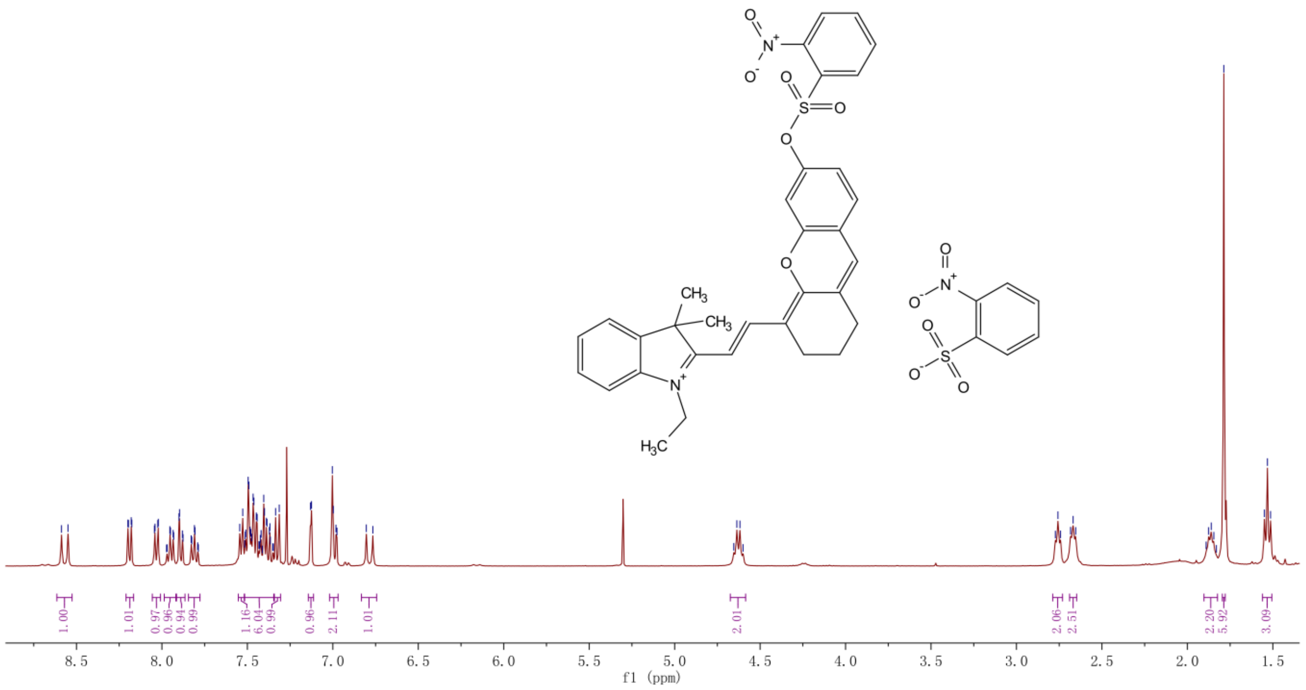

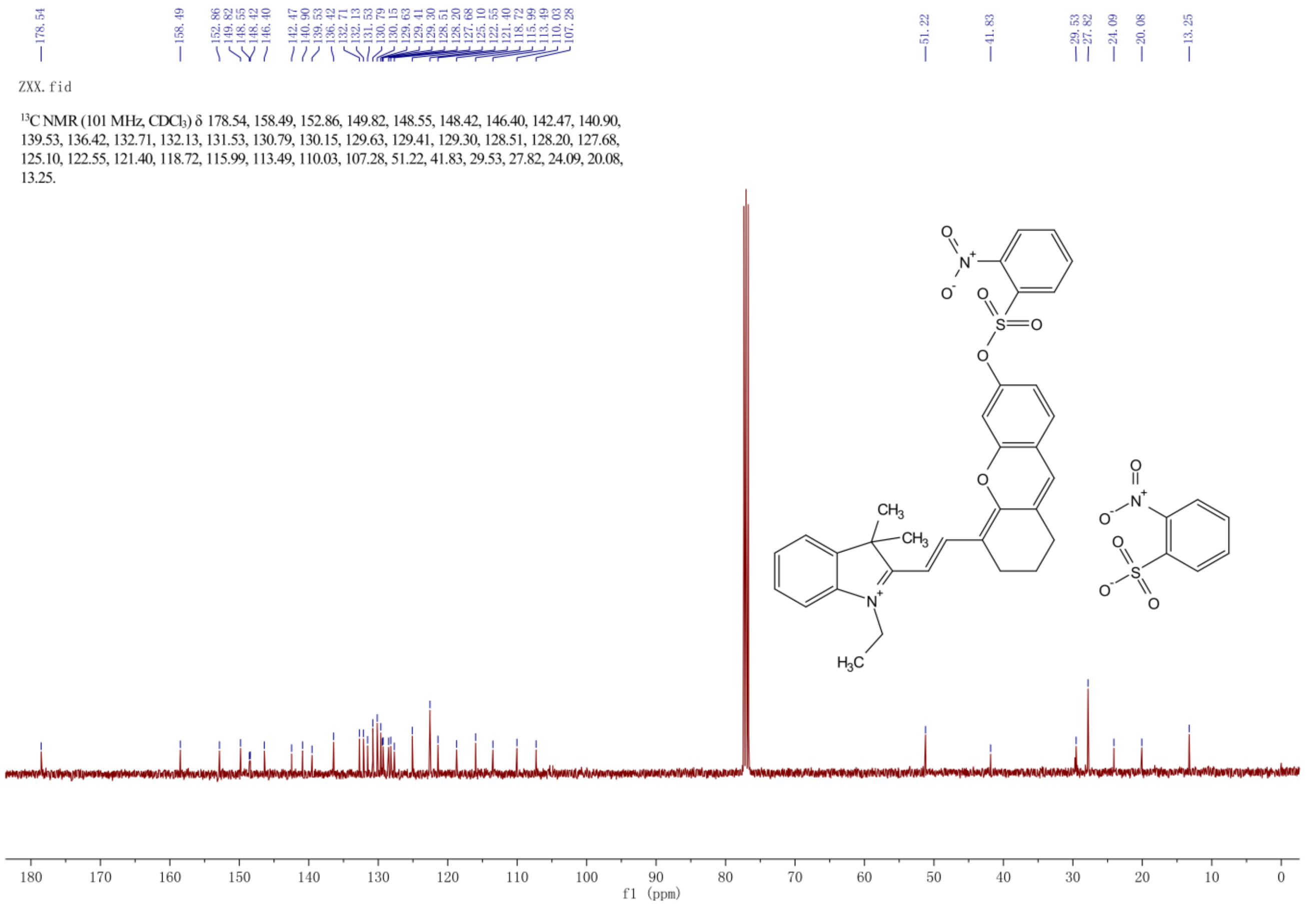

ZXX.fid

^{13}C NMR (101 MHz, $CDCl_3$) δ 178.54, 158.49, 152.86, 149.82, 148.55, 148.42, 146.40, 142.47, 140.90, 139.53, 136.42, 132.71, 132.13, 131.53, 130.79, 130.15, 129.63, 129.41, 129.30, 128.51, 128.20, 127.68, 125.10, 122.55, 121.40, 118.72, 115.99, 113.49, 110.03, 107.28, 51.22, 41.83, 29.53, 27.82, 24.09, 20.08, 13.25.

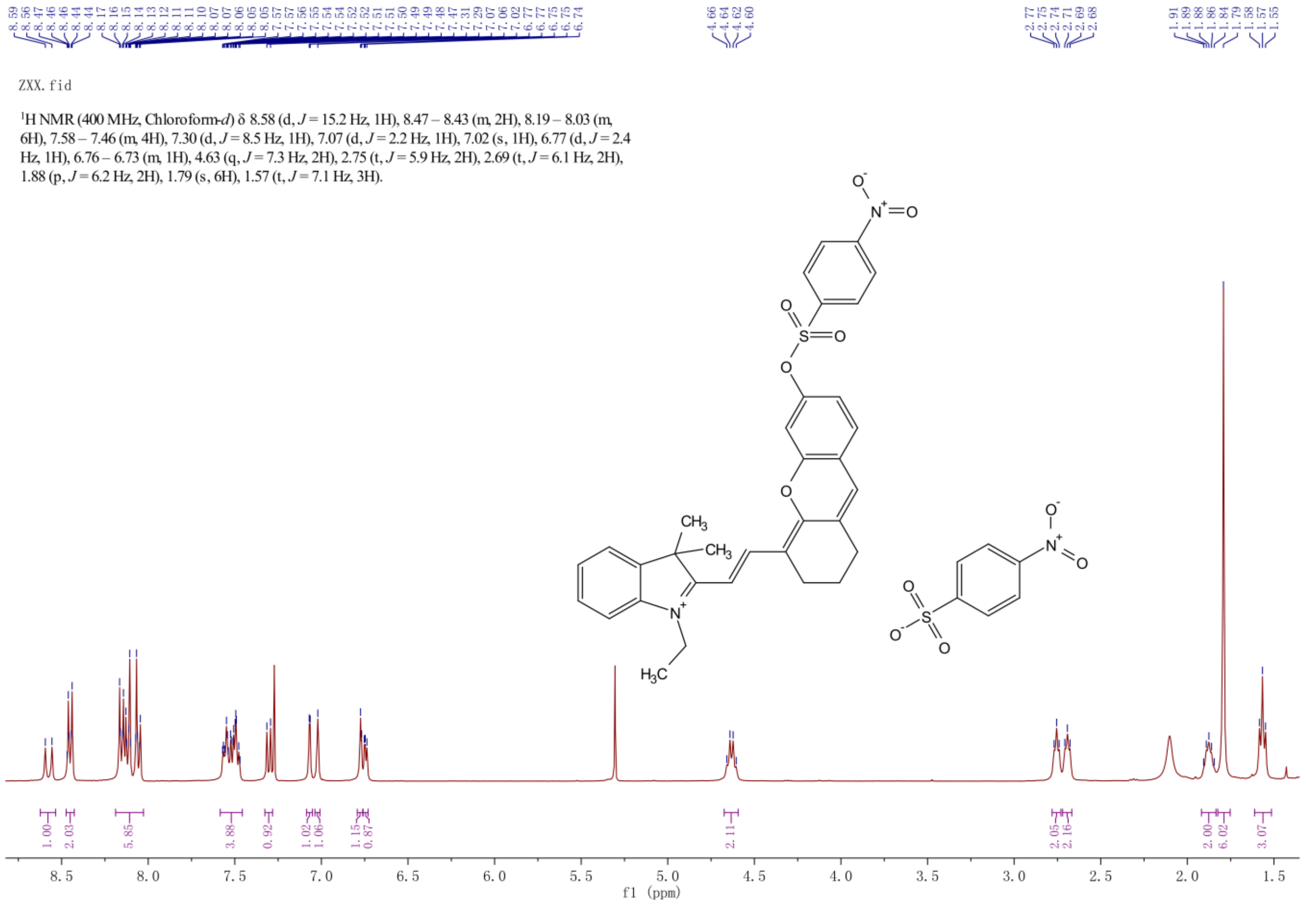

ZXX.fid

^{1}H NMR (400 MHz, Chloroform-*d*) δ 8.58 (d, *J* = 15.2 Hz, 1H), 8.47 – 8.43 (m, 2H), 8.19 – 8.03 (m, 6H), 7.58 – 7.46 (m, 4H), 7.30 (d, *J* = 8.5 Hz, 1H), 7.07 (d, *J* = 2.2 Hz, 1H), 7.02 (s, 1H), 6.77 (d, *J* = 2.4 Hz, 1H), 6.76 – 6.73 (m, 1H), 4.63 (q, *J* = 7.3 Hz, 2H), 2.75 (t, *J* = 5.9 Hz, 2H), 2.69 (t, *J* = 6.1 Hz, 2H), 1.88 (p, *J* = 6.2 Hz, 2H), 1.79 (s, 6H), 1.57 (t, *J* = 7.1 Hz, 3H).

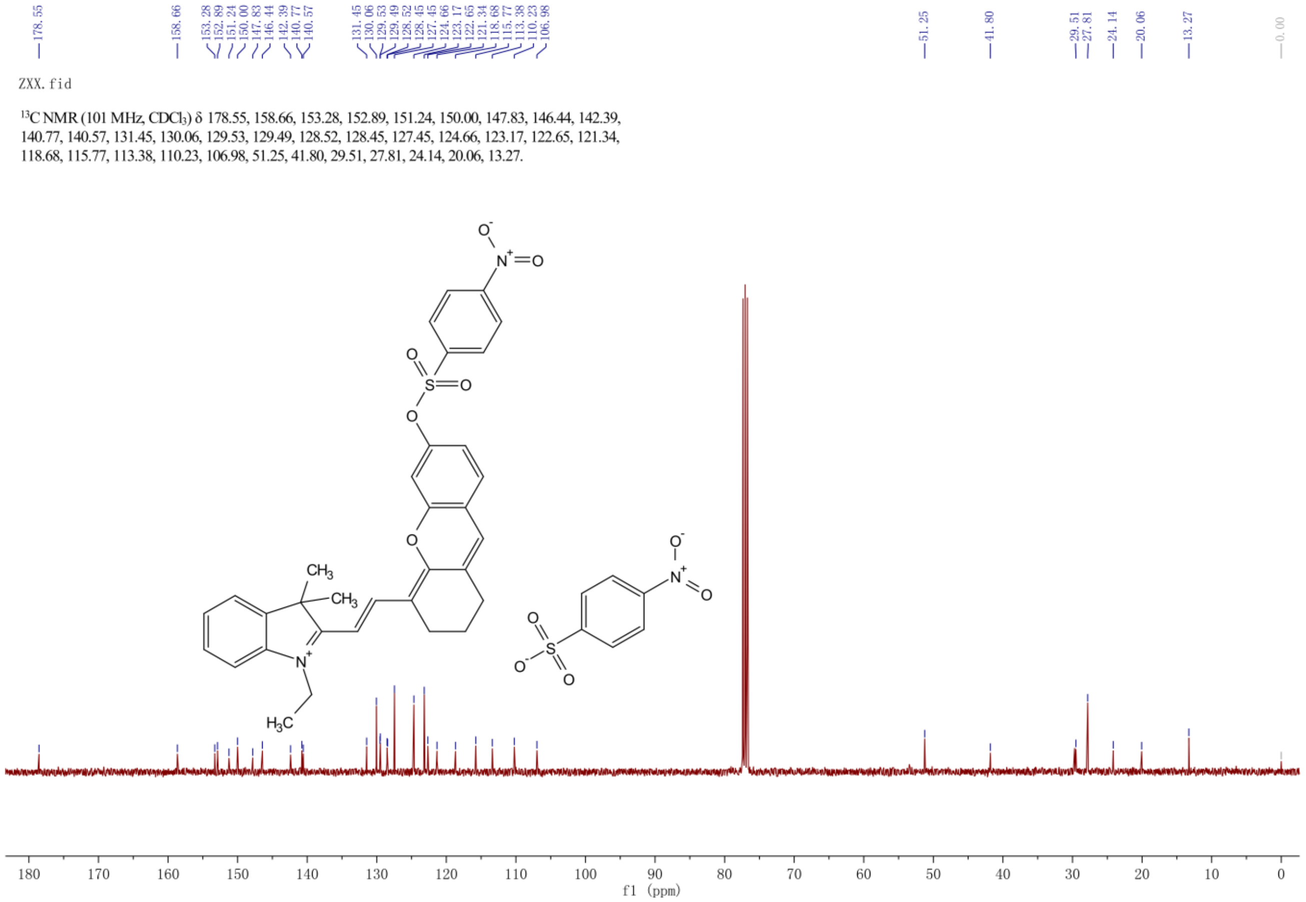

Cartesian coordinates (angstrom) for the optimized structures of probe models used for *ω* calculations

HCy1

C	-6.40153800	-3.00478000	-0.21206000
C	-5.04050900	-3.31584200	-0.23315500
C	-4.63716800	-4.64356000	-0.27099800
C	-5.61571800	-5.64529600	-0.28497800
C	-6.97383700	-5.31488100	-0.26069100
C	-7.39280300	-3.98141000	-0.22289900
C	-5.33885200	-0.97270900	-0.16088900
H	-3.58365000	-4.90855800	-0.28758400
H	-5.31653700	-6.68829700	-0.31314500
H	-7.72019300	-6.10305300	-0.26936500
H	-8.45039300	-3.74188100	-0.19899800
N	-6.53925900	-1.59301600	-0.17728800
C	-7.82679000	-0.90519000	-0.15287900
H	-8.62429000	-1.63136600	-0.29730500
H	-7.97588700	-0.40105800	0.80742400
H	-7.87615400	-0.17001800	-0.96099000
C	-4.22657500	-2.03320200	-0.20656500
C	-3.37314100	-1.91359100	-1.49477200
H	-2.78604500	-0.99154300	-1.51225600
H	-2.67726900	-2.75666000	-1.54563400
H	-4.00194300	-1.93893200	-2.38986500
C	-3.33886100	-1.98736100	1.06241500
H	-2.63944700	-2.82886900	1.04545900
H	-2.75424700	-1.06551400	1.11900900
H	-3.94318500	-2.06815200	1.97091900
C	-5.23323700	0.42479300	-0.09623300
H	-6.16093600	0.98188300	-0.02879900
C	-4.04017900	1.14185500	-0.09366600
H	-3.11075600	0.59110600	-0.15943000
C	-3.92169700	2.53953100	0.01082400
C	-2.64767700	3.12531500	0.04478800
C	-2.41011200	4.52027600	0.28732000
C	-5.13762800	3.44318800	0.12652700
H	-5.54018500	3.39220500	1.14927200
H	-5.93027200	3.07031300	-0.53260500
C	-4.83319800	4.90313100	-0.23460800
H	-5.70195100	5.53026500	-0.01135800

H	-4.64600300	4.98411400	-1.31274300
C	-3.60790100	5.40753900	0.53500800
H	-3.83158200	5.41452400	1.61198900
H	-3.36433000	6.43937400	0.26055800
O	-1.58516500	2.29997500	-0.15524600
C	-1.12171200	4.97860400	0.31252200
H	-0.92827900	6.03278500	0.49594500
C	-0.00891400	4.09908700	0.11704000
C	-0.29144300	2.73993500	-0.11589100
C	1.34457400	4.49372600	0.14681300
C	0.70210200	1.79146900	-0.33219500
C	2.35580400	3.56621300	-0.03684400
H	1.58796700	5.53595500	0.33041000
C	2.02506300	2.22283300	-0.27681600
H	0.44872000	0.75784700	-0.53097600
H	3.40038200	3.85348900	0.00195800
O	3.08083400	1.35597900	-0.52022700
S	3.12007600	-0.08650000	0.37523700
O	2.17264200	-0.99638900	-0.25153300
O	3.03410200	0.30159800	1.77258500
C	4.82858200	-0.51188700	-0.08007000
C	5.60234400	-1.39810900	0.68894800
C	5.40056300	0.07957900	-1.20976700
C	6.92740000	-1.66119200	0.36737000
C	6.72193400	-0.19683700	-1.56052700
H	4.82139500	0.76669400	-1.81074600
C	7.46444300	-1.05451300	-0.76027600
H	7.51756300	-2.33216100	0.97840400
N	5.04357000	-2.14886900	1.83275300
O	3.83410600	-2.36041500	1.80994800
O	5.82736400	-2.53549300	2.68876400
H	7.17613000	0.25039500	-2.43623100
N	8.87269100	-1.34079500	-1.12007700
O	9.31465200	-0.78123000	-2.11828300
O	9.48787100	-2.11307700	-0.39370800

HCy2

C	-6.61349500	-3.08126400	-0.21514200
C	-5.24624300	-3.36321600	-0.24180800
C	-4.81469600	-4.68194200	-0.28327800
C	-5.77157600	-5.70444400	-0.29549500
C	-7.13630800	-5.40309200	-0.26600200
C	-7.58359400	-4.07894300	-0.22444900
C	-5.59400000	-1.02685700	-0.16545800
H	-3.75578100	-4.92407500	-0.30412900
H	-5.45025900	-6.74076700	-0.32649800
H	-7.86570500	-6.20702400	-0.27365500
H	-8.64604300	-3.86218500	-0.19712000
N	-6.78125700	-1.67298000	-0.17717100
C	-8.08243200	-1.01226400	-0.14531000
H	-8.86599300	-1.75649000	-0.27283200
H	-8.23121100	-0.50159200	0.81157400
H	-8.15651500	-0.28616500	-0.95989700
C	-4.45952700	-2.06355200	-0.21632300
C	-3.61316700	-1.92445200	-1.50726600
H	-3.04485600	-0.99069300	-1.52530400
H	-2.90037200	-2.75299200	-1.56202500
H	-4.24447700	-1.96109900	-2.40022100
C	-3.56807400	-2.00119100	1.04943000
H	-2.85274000	-2.82911600	1.02924800
H	-3.00069800	-1.06852400	1.10448600
H	-4.16727000	-2.09409200	1.96018300
C	-5.51821200	0.37209900	-0.10177700
H	-6.45729200	0.90984100	-0.03410500
C	-4.33971700	1.11362500	-0.10051900
H	-3.39993100	0.58071000	-0.16504300
C	-4.24848300	2.51286100	0.00141400
C	-2.98533300	3.12315000	0.03686700
C	-2.77477500	4.52226700	0.27687700
C	-5.48118000	3.39405200	0.11322600
H	-5.88343400	3.33951800	1.13593000
H	-6.26638300	3.00433600	-0.54503600
C	-5.20328800	4.85808300	-0.25283100
H	-6.08423200	5.46968500	-0.03417400
H	-5.01487900	4.93822800	-1.33082100
C	-3.98951200	5.38832200	0.51761500
H	-4.21626600	5.39629600	1.59394900
H	-3.76441700	6.42324900	0.23894400
O	-1.90750700	2.31758000	-0.15856400
C	-1.49460800	5.00410100	0.30628200
H	-1.32143900	6.06216100	0.48789200
C	-0.36552200	4.14516100	0.11805500
C	-0.62189600	2.78052600	-0.11359100
C	0.98101700	4.56327600	0.15426400
C	0.38872800	1.84911400	-0.32254000
C	2.00872300	3.65296900	-0.02044300
H	1.20534500	5.61000200	0.33675600
C	1.70446300	2.30202900	-0.25861500
H	0.15294500	0.81143100	-0.52170900

H	3.04793300	3.95827700	0.02498400
O	2.77839300	1.45808100	-0.48656600
S	2.79989500	-0.02154200	0.35551700
O	1.89769800	-0.91605000	-0.35537400
O	2.62886300	0.31504000	1.75887700
C	4.53586900	-0.39885900	-0.02266800
C	5.28195200	-1.31272000	0.73996000
C	5.16387000	0.25702800	-1.08342400
C	6.62773700	-1.53592600	0.47802000
C	6.50903900	0.01612300	-1.36517600
H	4.60922200	0.96421500	-1.68420100
C	7.24380900	-0.86848300	-0.57888400
H	7.16932500	-2.24261200	1.09470700
N	4.67345600	-2.13438100	1.80507300
O	3.46417300	-2.33457400	1.72264400
O	5.41775800	-2.58557900	2.66598400
H	6.98102500	0.52291500	-2.19965100
C	8.72000200	-1.07745800	-0.83830100
F	9.45522000	-0.20157300	-0.12618000
F	9.10602400	-2.31747000	-0.48742000
F	9.01522800	-0.89584400	-2.13952000

HCy3

C	-6.18012600	-2.89667600	-0.17917100
C	-4.82675900	-3.23879900	-0.21131100
C	-4.45416900	-4.57524500	-0.25553100
C	-5.45538000	-5.55440500	-0.26433600
C	-6.80532200	-5.19312700	-0.22858100
C	-7.19341100	-3.85048800	-0.18433000
C	-5.07071800	-0.88961100	-0.13011700
H	-3.40710100	-4.86415700	-0.28088200
H	-5.18030900	-6.60388100	-0.29733900
H	-7.56957800	-5.96399500	-0.23310000
H	-8.24498500	-3.58690900	-0.15087500
N	-6.28522700	-1.48231800	-0.14031400
C	-7.55665300	-0.76561300	-0.10641200
H	-8.37049400	-1.47090800	-0.26322300
H	-7.69582600	-0.27244700	0.86112100
H	-7.58798800	-0.01770200	-0.90354200
C	-3.98344300	-1.97524800	-0.18786400
C	-3.13839200	-1.87169500	-1.48293100
H	-2.53070200	-0.96321300	-1.50312400
H	-2.46227700	-2.73018700	-1.54190400
H	-3.77527800	-1.88032300	-2.37261900
C	-3.08483000	-1.95314900	1.07391800
H	-2.40338700	-2.80908600	1.04853800
H	-2.48059100	-1.04400400	1.12913800
H	-3.68363000	-2.02446600	1.98687000
C	-4.93209300	0.50462500	-0.06017900
H	-5.84574800	1.08291100	0.01970600
C	-3.72258000	1.19394100	-0.06750300
H	-2.80683600	0.62240800	-0.14668800
C	-3.56997800	2.58776600	0.04124400
C	-2.28177900	3.14295700	0.05943700
C	-2.00738700	4.53116100	0.30065800
C	-4.76220800	3.51973700	0.17686500
H	-5.15134000	3.47495600	1.20508000
H	-5.57284200	3.16781600	-0.47186800
C	-4.42822000	4.97319300	-0.18429600
H	-5.27831400	5.62015200	0.05377900
H	-4.25523500	5.05341900	-1.26487100
C	-3.17989700	5.44589800	0.56852500
H	-3.38756200	5.45483600	1.64870400
H	-2.91599500	6.47254100	0.29336400
O	-1.24255600	2.29270700	-0.15699700
C	-0.70802800	4.95867600	0.30574500
H	-0.48676700	6.00769100	0.48738400
C	0.38015000	4.05337700	0.09131700
C	0.06176700	2.70149000	-0.13776100
C	1.74316700	4.41519700	0.09921200
C	1.02843400	1.72955200	-0.37049100
C	2.72855700	3.46379500	-0.10110300
H	2.01461800	5.45108700	0.27938200
C	2.36235800	2.12843700	-0.33613800
H	0.74670900	0.70264100	-0.56575700
H	3.78023800	3.72582100	-0.07865100
O	3.39367800	1.23846300	-0.59554300
S	3.40046600	-0.21831500	0.28008200
O	2.42367200	-1.09213600	-0.35351100
O	3.33022700	0.15341900	1.68294900
C	5.09300000	-0.68122900	-0.19084200
C	5.84877400	-1.59903000	0.55729500
C	5.67751000	-0.08913000	-1.31345800
C	7.16389300	-1.89117700	0.22364200
C	6.98779000	-0.39551600	-1.67420300
H	5.11493600	0.62278700	-1.90134800
C	7.74173800	-1.28964400	-0.90236500
H	7.71867600	-2.59127000	0.83626200
N	5.27715300	-2.35420800	1.69180000

O	4.06171400	-2.52801600	1.67728400
O	6.05543100	-2.78198400	2.53386700
H	7.42858600	0.06597700	-2.55112200
C	9.09491400	-1.59625500	-1.26466800
N	10.19095600	-1.83942700	-1.56487500

HCy4

C	-6.92178500	-2.92281400	-0.42149800
C	-5.56353300	-3.24613200	-0.40327700
C	-5.17001800	-4.57553300	-0.47215600
C	-6.15541500	-5.56699800	-0.55727400
C	-7.51061800	-5.22463600	-0.57202500
C	-7.91968200	-3.88925700	-0.50339000
C	-5.84419900	-0.90390400	-0.26195100
H	-4.11877100	-4.84966300	-0.45846900
H	-5.86384600	-6.61118900	-0.61043300
H	-8.26269800	-6.00482300	-0.63591400
H	-8.97544300	-3.64071500	-0.51106700
N	-7.04894300	-1.51209100	-0.34201000
C	-8.32961900	-0.81187600	-0.33879800
H	-9.13068300	-1.52712900	-0.51458900
H	-8.49769100	-0.32227300	0.62586300
H	-8.35171200	-0.06235500	-1.13510500
C	-4.73990200	-1.97336400	-0.30101900
C	-3.83371900	-1.82007300	-1.54887300
H	-3.23636000	-0.90521100	-1.51155500
H	-3.14494300	-2.66888800	-1.60019300
H	-4.42583900	-1.80793300	-2.46895500
C	-3.90388000	-1.98004400	1.00351400
H	-3.20987500	-2.82595000	0.98457600
H	-3.31585700	-1.06563400	1.11676300
H	-4.54513100	-2.08805500	1.88345300
C	-5.72901700	0.48893300	-0.14816000
H	-6.65299000	1.05410100	-0.09790900
C	-4.52887100	1.19189400	-0.07834600
H	-3.60431500	0.63149000	-0.12790200
C	-4.39731600	2.58267100	0.07666100
C	-3.11678700	3.14875100	0.17533300
C	-2.86879600	4.52906900	0.47787100
C	-5.60380400	3.49981200	0.18197100
H	-6.04167000	3.42113800	1.18836000
H	-6.37895300	3.16095500	-0.51539000
C	-5.26599700	4.96616500	-0.11868600
H	-6.13335300	5.59892100	0.09410500
H	-5.03844400	5.07982400	-1.18610300
C	-4.06237600	5.42633500	0.71095200
H	-4.32571100	5.40247900	1.77868400
H	-3.79443500	6.46265800	0.47919000
O	-2.06068800	2.31546200	-0.01947600
C	-1.57465700	4.96414900	0.57170800
H	-1.37278900	6.00746100	0.80263300
C	-0.46920100	4.07413600	0.38953100
C	-0.76247300	2.73000600	0.09091600
C	0.88898700	4.44027600	0.49478900
C	0.22193900	1.77218900	-0.12050100
C	1.88977300	3.50027000	0.32179100
H	1.14259500	5.46934600	0.73162600
C	1.55049000	2.17106000	0.01347400
H	-0.04249500	0.75264200	-0.37047900
H	2.93600200	3.76494300	0.42515600
O	2.59902700	1.29808200	-0.20517700
S	2.51612500	-0.22134000	0.58519100
O	1.61057900	-1.03337600	-0.21532200
O	2.27737500	0.09327500	1.98335100
C	4.25585500	-0.67575300	0.28892000
C	4.86600400	-1.75256500	0.96913900
C	4.98653500	0.06020600	-0.62539700
C	6.21942900	-1.98853600	0.77962500
C	6.35890600	-0.21080200	-0.90863200
H	4.49088400	0.84817900	-1.16845300
C	6.96373000	-1.21176600	-0.09408200
H	6.70367400	-2.77317100	1.34754600
N	4.14188300	-2.65826200	1.85165900
O	2.91079300	-2.59431500	1.82574500
O	4.79262400	-3.43979700	2.53853100
N	8.42022200	-1.39392700	-0.00834000
O	9.11288000	-0.37869800	-0.08455700
O	8.84257700	-2.52903100	0.19580200
N	6.98404800	0.44141500	-1.92448400
C	8.11896900	-0.12204900	-2.65766500
H	7.96139300	0.06512100	-3.72440300
H	9.06998200	0.31883200	-2.34471900
H	8.16740900	-1.20343500	-2.51431600
C	6.40829100	1.65257200	-2.50472300
H	7.20530300	2.20154800	-3.01032500
H	5.62024100	1.43004600	-3.23802400
H	6.00324900	2.30107500	-1.72412700

HCy5

C	-6.73039700	-3.01323600	-0.35754800
C	-5.36718400	-3.31499700	-0.34274900
C	-4.95353500	-4.63938300	-0.38595900
C	-5.92404300	-5.64744700	-0.44214200
C	-7.28453600	-5.32640500	-0.45383300
C	-7.71383000	-3.99636400	-0.41092400
C	-5.68355800	-0.97485400	-0.25099300
H	-3.89810700	-4.89695800	-0.37482900
H	-5.61672800	-6.68794200	-0.47527800
H	-8.02476400	-6.11932200	-0.49530200
H	-8.77336400	-3.76431300	-0.41627000
N	-6.87900400	-1.60314000	-0.30802200
C	-8.17066200	-0.92315000	-0.31046700
H	-8.96171700	-1.65539200	-0.45924600
H	-8.33705900	-0.41115000	0.64273400
H	-8.21233500	-0.19528600	-1.12586900
C	-4.56306400	-2.02755400	-0.27501300
C	-3.66960200	-1.88690900	-1.53351700
H	-3.08770400	-0.96145200	-1.52113500
H	-2.96721600	-2.72519900	-1.57211500
H	-4.26912400	-1.90494300	-2.44866400
C	-3.71634700	-1.99260500	1.02219500
H	-3.01162500	-2.82979600	1.01694100
H	-3.13925200	-1.06837700	1.10891200
H	-4.34872300	-2.08827700	1.90993800
C	-5.58972900	0.42224300	-0.17195000
H	-6.52261100	0.97344800	-0.13319500
C	-4.40141600	1.14625700	-0.12338400
H	-3.46760100	0.60016300	-0.15747300
C	-4.29383700	2.54348400	-0.00958500
C	-3.02419500	3.13500700	0.07471900
C	-2.80229800	4.53044700	0.32648300
C	-5.51686100	3.44192600	0.06268600
H	-5.95450900	3.39080100	1.07093300
H	-6.28470800	3.06512600	-0.62302400
C	-5.20547200	4.90264200	-0.28946200
H	-6.08472300	5.52641300	-0.10037000
H	-4.97795300	4.98244300	-1.35991200
C	-4.01229300	5.41380200	0.52488900
H	-4.27740200	5.42349900	1.59241400
H	-3.76220100	6.44573300	0.25656900
O	-1.95185600	2.31430300	-0.08338400
C	-1.51723500	4.99342400	0.40383300
H	-1.33540900	6.04849400	0.59419500
C	-0.39450000	4.11824200	0.25552100
C	-0.66201700	2.75801300	0.01097700
C	0.95564200	4.51651500	0.34333000
C	0.34197900	1.81204200	-0.16092600
C	1.97579100	3.59131700	0.20520300
H	1.18804100	5.55955000	0.53658200
C	1.66095700	2.24555800	-0.04736700
H	0.10044800	0.77749300	-0.36844500
H	3.01684100	3.88143900	0.29016100
O	2.72643400	1.38210600	-0.24074800
S	2.72670900	-0.05829900	0.66883900
O	1.78208700	-0.95183900	0.01466400
O	2.60051900	0.35315900	2.05647900
C	4.44391300	-0.52172700	0.26581500
C	5.12497400	-1.55299700	0.95020100
C	5.11900800	0.19054700	-0.70992100
C	6.43677900	-1.85016300	0.62303400
C	6.45971900	-0.09270400	-1.06146000
H	4.64995800	1.01561300	-1.22552600
C	7.09091400	-1.16202600	-0.39441900
H	6.94573500	-2.65121900	1.14485300
N	4.48916000	-2.39756400	1.96759300
O	3.26251200	-2.34759000	2.04128600
O	5.21403600	-3.10722100	2.65533900
N	8.40076800	-1.69982300	-0.79142800
O	8.64378000	-1.72201100	-1.99725100
O	9.12586900	-2.13712300	0.09545900
O	6.96435600	0.73542500	-1.97811100
C	8.36546800	1.06772000	-2.06085500
H	8.87647500	0.39846700	-2.75194200
H	8.38748600	2.09370200	-2.43014400
H	8.83292400	1.02006000	-1.07226000

HCy6

C	6.11131500	-2.64431800	0.21591200
C	4.77637400	-3.04910000	0.27880400
C	4.46837800	-4.39925400	0.37392100
C	5.51495500	-5.32942100	0.40291800
C	6.84542700	-4.90599200	0.33656700
C	7.16859200	-3.54881300	0.24072400
C	4.90574900	-0.69460700	0.11696800
H	3.43649900	-4.73579000	0.42367300
H	5.29062600	-6.38890600	0.47598400
H	7.64573700	-5.63913800	0.35781200

H	8.20610800	-3.23731400	0.18540100
N	6.14805800	-1.22931300	0.12634800
C	7.38158500	-0.45335900	0.04894900
H	8.23316200	-1.11737300	0.18364200
H	7.46692000	0.03823700	-0.92555700
H	7.40199100	0.30230300	0.83951000
C	3.87270500	-1.82864200	0.22438400
C	3.03828300	-1.72288900	1.52585900
H	2.38800500	-0.84426900	1.52427400
H	2.40457900	-2.60994000	1.62185400
H	3.68506900	-1.67159300	2.40699100
C	2.96019900	-1.89266700	-1.02612600
H	2.32292900	-2.78018000	-0.96508400
H	2.30995300	-1.01750500	-1.10332900
H	3.55211000	-1.96368800	-1.94362700
C	4.70027100	0.68790800	0.01227000
H	5.58424100	1.30795600	-0.08785200
C	3.45796700	1.31877600	0.01269200
H	2.57187500	0.70448900	0.10788200
C	3.23677000	2.69973900	-0.11996500
C	1.92114200	3.19056700	-0.14174500
C	1.57767000	4.55972600	-0.39497400
C	4.37974600	3.68875500	-0.27460800
H	4.76262900	3.65390900	-1.30559700
H	5.21215700	3.38407600	0.37042700
C	3.97561400	5.12687500	0.07575600
H	4.79135100	5.81328100	-0.17218400
H	3.80407700	5.20774600	1.15655000
C	2.70163500	5.53057500	-0.67440400
H	2.90447300	5.54321800	-1.75547700
H	2.38780800	6.54453200	-0.40442100
O	0.92746200	2.29178300	0.08567800
C	0.25733600	4.92116100	-0.39834000
H	-0.01661700	5.95647700	-0.58724700
C	-0.78237900	3.96549800	-0.17322200
C	-0.39649300	2.63269800	0.06567900
C	-2.16293400	4.25622500	-0.18033900
C	-1.31031500	1.61389400	0.30715700
C	-3.09629500	3.25673700	0.02748300
H	-2.48794700	5.27545100	-0.36725100
C	-2.66479600	1.94004600	0.27036900
H	-0.97316000	0.60559000	0.51093400
H	-4.16024800	3.46336900	0.00341000
O	-3.65217400	1.01049200	0.52683100
S	-3.51922300	-0.51347200	-0.23868500
O	-2.53326700	-1.26554500	0.52556200
O	-3.35785100	-0.22264300	-1.65392500
C	-5.21000800	-1.04587600	0.14635600
C	-5.83947000	-2.08254300	-0.56200900
C	-5.91873700	-0.39287800	1.15576100
C	-7.15926900	-2.42445300	-0.28394100
C	-7.24206200	-0.74269500	1.46359600
H	-5.44394500	0.40837800	1.70581800
C	-7.85224700	-1.76086800	0.72237400
H	-7.61552500	-3.22313700	-0.85636400
H	-8.87740200	-2.04733900	0.93914400
N	-5.13558000	-2.89911800	-1.56244700
O	-3.90828300	-2.93402300	-1.48678500
O	-5.81491700	-3.51439000	-2.37623600
C	-7.99377700	-0.01593300	2.55060700
H	-8.69497500	0.70947700	2.11894900
H	-8.58097800	-0.71209500	3.15817300
H	-7.31664900	0.52972500	3.21360700

HCy7

C	-6.02331800	-2.84252100	-0.15944700
C	-4.67110100	-3.18787400	-0.20544300
C	-4.30220300	-4.52465700	-0.26604100
C	-5.30566400	-5.50156300	-0.27726600
C	-6.65424100	-5.13731700	-0.22777000
C	-7.03863600	-3.79415700	-0.16682400
C	-4.90773400	-0.83858500	-0.09868400
H	-3.25595300	-4.81544200	-0.30223500
H	-5.03330100	-6.55128500	-0.32301400
H	-7.42056200	-5.90617700	-0.23449900
H	-8.08930300	-3.52840800	-0.12284100
N	-6.12474800	-1.42883400	-0.10515200
C	-7.39298100	-0.70867000	-0.05407200
H	-8.21082100	-1.41004000	-0.20808300
H	-7.52171300	-0.22162700	0.91810300
H	-7.42956000	0.04542100	-0.84531500
C	-3.82387600	-1.92691100	-0.17605100
C	-2.98869100	-1.81362900	-1.47670200
H	-2.37733600	-0.90758300	-1.49276300
H	-2.31621300	-2.67387600	-1.54989200
H	-3.63290400	-1.81070000	-2.36118300
C	-2.91496800	-1.92035500	1.07846000
H	-2.23581700	-2.77759200	1.03898400
H	-2.30765500	-1.01359000	1.13820000

H	-3.50677700	-1.99931300	1.99537300
C	-4.76464500	0.55321100	-0.01652300
H	-5.67531100	1.13445100	0.07602700
C	-3.55114300	1.23792100	-0.02851500
H	-2.63968700	0.66161200	-0.12046300
C	-3.38816000	2.62822400	0.08795800
C	-2.09383700	3.17358400	0.09485900
C	-1.80509900	4.55749400	0.33464100
C	-4.57018600	3.57008800	0.24210900
H	-4.94501100	3.52727700	1.27574600
H	-5.39335400	3.22663700	-0.39530800
C	-4.22799000	5.02126900	-0.12111300
H	-5.06954300	5.67525900	0.12816800
H	-4.06759600	5.10133100	-1.20367300
C	-2.96623700	5.48271300	0.61657100
H	-3.16124200	5.49436100	1.69910400
H	-2.69686100	6.50697200	0.33755200
O	-1.06597900	2.31470300	-0.13376400
C	-0.50063600	4.97298100	0.32399500
H	-0.26813300	6.02008500	0.50324900
C	0.57559400	4.05932200	0.09596700
C	0.24281800	2.70992700	-0.12911100
C	1.94322100	4.40620100	0.08653400
C	1.19494500	1.72737500	-0.37246700
C	2.91459000	3.44420500	-0.12398200
H	2.22823900	5.43923900	0.26253200
C	2.53520800	2.10885400	-0.35256400
H	0.89688200	0.70487700	-0.56587100
H	3.96934900	3.69453400	-0.11316600
O	3.55794200	1.21937800	-0.61220200
S	3.49105400	-0.30900900	0.15244700
O	2.51899400	-1.09614900	-0.59457500
O	3.34196200	-0.02932100	1.57147400
C	5.18872000	-0.77560300	-0.26209000
C	5.88447000	-1.77434700	0.44201900
C	5.85605700	-0.11260900	-1.29229300
C	7.20799200	-2.07080500	0.14826300
C	7.18026700	-0.43268600	-1.59847300
H	5.35047700	0.66347400	-1.85018000
C	7.88443700	-1.40400100	-0.88212300
H	7.69637600	-2.84374000	0.73125300
N	5.23295400	-2.60753500	1.46975200
O	4.01072300	-2.71961500	1.39649900
O	5.94966800	-3.15791900	2.29710600
H	7.67034000	0.09656500	-2.41062400
C	9.32096600	-1.73727100	-1.19458400
H	9.97629600	-1.45707900	-0.36115100
H	9.44978600	-2.81277300	-1.35925400
H	9.66849900	-1.21161400	-2.08786000

HCy8

C	-6.16324400	-3.06272600	-0.09682100
C	-4.80291200	-3.37774700	-0.10074400
C	-4.40283900	-4.70690900	-0.08893900
C	-5.38362600	-5.70640800	-0.07141500
C	-6.74092200	-5.37205500	-0.06495600
C	-7.15664900	-4.03706200	-0.07708000
C	-5.09526000	-1.03261300	-0.11836600
H	-3.34988200	-4.97475400	-0.09214600
H	-5.08693400	-6.75045900	-0.06145500
H	-7.48928500	-6.15821500	-0.04917300
H	-8.21379200	-3.79455000	-0.06790200
N	-6.29759200	-1.65052000	-0.11466300
C	-7.58276700	-0.95815500	-0.12222200
H	-8.38375000	-1.68875000	-0.21564300
H	-7.72238900	-0.39685800	0.80719300
H	-7.63678100	-0.27222100	-0.97251400
C	-3.98570500	-2.09691200	-0.11723600
C	-3.12473800	-2.02652400	-1.40370100
H	-2.53825000	-1.10524300	-1.45254400
H	-2.42865200	-2.87093000	-1.41882700
H	-3.74825600	-2.08589600	-2.30085100
C	-3.10509800	-2.00665300	1.15439600
H	-2.41028700	-2.85195900	1.17343400
H	-2.51561700	-1.08645500	1.17951000
H	-3.71509700	-2.04981400	2.06167700
C	-4.98659600	0.36544000	-0.11641000
H	-5.91402800	0.92649300	-0.09146300
C	-3.79203500	1.08134800	-0.12906800
H	-2.86204400	0.52766000	-0.14435700
C	-3.67492000	2.48212500	-0.10377000
C	-2.40147300	3.07145700	-0.07343400
C	-2.17185700	4.48120500	0.07016400
C	-4.89326400	3.38983600	-0.07780600
H	-5.31708200	3.41135900	0.93737300
H	-5.67161900	2.97013800	-0.72543700
C	-4.58172400	4.82093100	-0.53559400
H	-5.45595700	5.46031100	-0.37817400
H	-4.36929500	4.82472400	-1.61205400

C	-3.37529600	5.38184200	0.22416900
H	-3.62449300	5.46671300	1.29219600
H	-3.12525000	6.39141600	-0.11869700
O	-1.33361400	2.23646600	-0.17920000
C	-0.88512300	4.94480300	0.08493100
H	-0.69787900	6.01080900	0.18947800
C	0.23334700	4.05782400	-0.02097500
C	-0.04112800	2.68265600	-0.14842500
C	1.58408900	4.46274600	-0.00044200
C	0.96107700	1.72524000	-0.26336500
C	2.60235400	3.52976100	-0.09473000
H	1.81833800	5.51814300	0.10174300
C	2.28019100	2.16986800	-0.22818300
H	0.72271100	0.67489000	-0.37242500
H	3.64525100	3.82417100	-0.06733300
O	3.33188500	1.28017900	-0.39307600
S	3.50676800	0.07452600	0.78017400
O	2.46588900	-0.92659800	0.55118900
O	3.60399100	0.73725000	2.07498600
C	5.10453600	-0.55320100	0.24983300
C	5.75342500	-1.46700500	1.10762400
C	5.63240700	-0.17269800	-0.98761100
C	6.97895300	-1.97629100	0.66570600
C	6.85431200	-0.69375300	-1.40243300
H	5.10031000	0.52498000	-1.62008800
C	7.50417500	-1.58827000	-0.56135600
H	7.53153000	-2.68104300	1.27543900
H	7.29631500	-0.42015100	-2.35207700
N	8.80288200	-2.15480600	-0.98891000
O	9.24285200	-1.79137500	-2.07616500
O	9.34990800	-2.94870200	-0.22872000
C	5.20092600	-1.90319100	2.44414500
H	5.17521500	-1.06941700	3.15287800
H	4.17811800	-2.28392000	2.35062500
H	5.82054300	-2.69750700	2.86638900

HCy9

C	5.94291400	-2.61735400	0.07432000
C	4.62335800	-3.03152200	0.26694700
C	4.34173900	-4.37962100	0.44092300
C	5.39901000	-5.29779400	0.41944700
C	6.71393100	-4.86470300	0.22595000
C	7.01041900	-3.50971300	0.04881100
C	4.70958400	-0.68360000	0.01029500
H	3.32220200	-4.72372000	0.59109400
H	5.19528600	-6.35542400	0.55391700
H	7.52281200	-5.58854300	0.21165500
H	8.03628900	-3.19067900	-0.10052900
N	5.95360700	-1.20708100	-0.07670700
C	7.16425500	-0.42255800	-0.29762200
H	8.02390700	-1.08871900	-0.33072500
H	7.10058400	0.11550000	-1.24838300
H	7.30720700	0.29500000	0.51630700
C	3.70310100	-1.82240600	0.24197100
C	2.97478200	-1.67517900	1.60152300
H	2.31710400	-0.80219000	1.61980100
H	2.36054300	-2.56299200	1.78085700
H	3.68939100	-1.58563700	2.42532700
C	2.69282900	-1.94235900	-0.92717300
H	2.07860600	-2.83660100	-0.78412300
H	2.02277700	-1.08035400	-0.97944400
H	3.20873200	-2.03665800	-1.88742500
C	4.48345100	0.69489900	-0.10490900
H	5.35353300	1.32011000	-0.27144900
C	3.23910900	1.31713800	-0.03328300
H	2.36416500	0.69793200	0.11761000
C	3.00319800	2.69661900	-0.15952500
C	1.68619000	3.18162700	-0.11707700
C	1.32639300	4.55201100	-0.34181500
C	4.13218800	3.69144600	-0.37079100
H	4.45506300	3.66803700	-1.42247200
H	5.00121200	3.38486100	0.22270700
C	3.74336000	5.12435200	0.01570700
H	4.54330100	5.81597100	-0.26676500
H	3.62808300	5.19276300	1.10478300
C	2.43141000	5.53177200	-0.66311000
H	2.57911400	5.56063300	-1.75278400
H	2.12719400	6.54032900	-0.36345000
O	0.70667000	2.27669800	0.14662300
C	0.00630200	4.90816700	-0.28118000
H	-0.27965500	5.94430900	-0.44616400
C	-1.01902000	3.94597200	-0.02040800
C	-0.61823600	2.61238700	0.18709800
C	-2.39933700	4.23201500	0.03390400
C	-1.51835700	1.58740800	0.45385400
C	-3.32023900	3.22668200	0.26786800
H	-2.73501400	5.25221800	-0.12696400
C	-2.87394100	1.90948900	0.47582300
H	-1.17024200	0.57763500	0.63079700

H	-4.38495500	3.42946800	0.29038400
O	-3.84668700	0.97069500	0.75897400
S	-3.74855700	-0.53184700	-0.04749400
O	-2.74576800	-1.31156100	0.66469700
O	-3.63298700	-0.21196300	-1.46092300
C	-5.43034500	-1.05913800	0.37884300
C	-6.10288800	-2.05624100	-0.34838200
C	-6.09519100	-0.43867400	1.43870100
C	-7.41767300	-2.39740500	-0.04960700
C	-7.40711700	-0.80025200	1.75478900
H	-5.59724200	0.33433900	2.00746500
C	-8.07268200	-1.76884400	1.00772800
H	-7.89968800	-3.16262000	-0.64607900
H	-9.09481300	-2.04397900	1.24688000
N	-5.44054500	-2.83782900	-1.40881200
O	-4.21563700	-2.92334700	-1.34924200
O	-6.15309500	-3.37606600	-2.24734600
H	-7.90498800	-0.31044000	2.58570800

HCy10

C	-6.13245500	-2.91619200	0.02273500
C	-4.78366800	-3.27767800	0.02188300
C	-4.42901800	-4.61925100	0.05565000
C	-5.44306500	-5.58449200	0.09204200
C	-6.78807000	-5.20395000	0.09559100
C	-7.15817400	-3.85586600	0.06143700
C	-4.99546700	-0.92435700	-0.03578400
H	-3.38569500	-4.92265400	0.05498900
H	-5.18199500	-6.63771300	0.11920800
H	-7.56280900	-5.96369100	0.12642600
H	-8.20640800	-3.57729100	0.06895100
N	-6.21856200	-1.50100700	-0.02011400
C	-7.47898000	-0.76516400	-0.03962900
H	-8.30468300	-1.46872100	-0.12576800
H	-7.60110000	-0.18681700	0.88181600
H	-7.50774200	-0.08906700	-0.89901500
C	-3.92292700	-2.02616000	-0.01807900
C	-3.06377300	-2.00739900	-1.30737500
H	-2.44766400	-1.10690000	-1.37442700
H	-2.39556200	-2.87418800	-1.30906200
H	-3.69178000	-2.06231100	-2.20170200
C	-3.03693300	-1.94508200	1.25023200
H	-2.37039900	-2.81251900	1.28176500
H	-2.41735800	-1.04463000	1.25967300
H	-3.64609000	-1.95336300	2.15907100
C	-4.83836200	0.46836600	-0.05956700
H	-5.74530800	1.06244100	-0.04537900
C	-3.61882000	1.14174200	-0.08589600
H	-2.70896700	0.55534500	-0.08683300
C	-3.45145200	2.53686000	-0.09422400
C	-2.15690200	3.08067500	-0.07552700
C	-1.87587600	4.48384300	0.02326000
C	-4.63518800	3.48978500	-0.09772700
H	-5.05750800	3.55980600	0.91594400
H	-5.42903400	3.07894300	-0.73206000
C	-4.27016000	4.89276400	-0.60059200
H	-5.12050800	5.56874700	-0.46679900
H	-4.05553700	4.85356300	-1.67590800
C	-3.04510700	5.43331600	0.14419100
H	-3.29334300	5.56274000	1.20798300
H	-2.75720700	6.42109300	-0.23108300
O	-1.12031500	2.20341000	-0.14928200
C	-0.57195600	4.89937300	0.02429000
H	-0.34541500	5.96073700	0.09256900
C	0.51165300	3.96921000	-0.05078700
C	0.18753300	2.60159200	-0.13085700
C	1.87792200	4.32047500	-0.04514700
C	1.15060800	1.60163200	-0.21283700
C	2.85811900	3.34652900	-0.10293300
H	2.15463300	5.36871900	0.01760400
C	2.48746200	1.99276300	-0.18539900
H	0.86012700	0.56238900	-0.30111800
H	3.91163700	3.60165700	-0.08661000
O	3.53004700	1.08925300	-0.29545500
S	3.44083500	-0.38745900	0.52800800
O	2.53536000	-1.25782500	-0.21622200
O	3.21656800	-0.09683500	1.93780200
C	5.13143600	-0.87937400	0.24502300
C	6.08927900	-0.57111200	1.21362000
C	5.44224200	-1.57878600	-0.92334400
C	7.40737000	-0.96630000	0.99917100
C	6.76129400	-1.97097600	-1.13595300
H	4.66839000	-1.81860300	-1.64377000
C	7.71535800	-1.65417400	-0.17194600
H	8.18841700	-0.75512900	1.71894100
H	7.05671300	-2.51537700	-2.02418200
N	9.11852900	-2.07270300	-0.40020300
O	9.36061400	-2.68044500	-1.43868200
O	9.93884200	-1.78045600	0.46452800
H	5.80662600	-0.04436300	2.11821700

Cartesian coordinates (angstrom) for the optimized structures of probe models used for P_k^+ calculations

HCy1

C	-6.79215600	-3.03612600	0.12624400
C	-5.45714600	-3.47898000	0.11308200
C	-5.16368300	-4.83287600	0.18381900
C	-6.22527800	-5.74608800	0.26773800
C	-7.55256200	-5.30101900	0.28009800
C	-7.85360700	-3.93816200	0.20951700
C	-5.66950600	-1.16168400	-0.01423300
H	-4.13610000	-5.19032000	0.17595500
H	-6.01467500	-6.81069200	0.32438800
H	-8.35937500	-6.02631000	0.34599700
H	-8.87827000	-3.57911100	0.21892100
N	-6.88809200	-1.63813900	0.04820100
C	-4.57107500	-2.25088000	0.01641200
C	-3.73799900	-2.27471500	-1.28553300
H	-3.13783700	-1.36664500	-1.39898500
H	-3.05606900	-3.13288300	-1.27708700
H	-4.38604100	-2.36621700	-2.16314600
C	-3.66003800	-2.13085900	1.25975000
H	-2.98203200	-2.99049100	1.31035900
H	-3.05083400	-1.22235500	1.23196700
H	-4.25356300	-2.11432000	2.17937300
C	-5.49143900	0.26912700	-0.10102000
H	-6.43560700	0.80767300	-0.10160700
C	-4.31442400	0.95122100	-0.16823600
H	-3.38335100	0.39778900	-0.15636700
C	-4.18128200	2.38570500	-0.23089600
C	-2.93980200	2.97292400	-0.24765700
C	-2.70363700	4.40168800	-0.21459400
C	-5.41408800	3.27016100	-0.25982000
H	-5.85030100	3.33288000	0.74902900
H	-6.17935300	2.79890900	-0.88714500
C	-5.12773300	4.68036400	-0.79028600
H	-6.00789600	5.31870600	-0.65460400
H	-4.92743500	4.63203800	-1.86842500
C	-3.91656700	5.29013600	-0.07727600
H	-4.15199000	5.40620400	0.99165300
H	-3.68788000	6.29169800	-0.45891400
O	-1.83865100	2.12861700	-0.28648500
C	-1.43632900	4.88401000	-0.27014500
H	-1.25684800	5.95603300	-0.24726600
C	-0.30052400	3.99216100	-0.33375400
C	-0.56273000	2.60806700	-0.32922500
C	1.03712900	4.41440200	-0.38404700
C	0.46395900	1.66933400	-0.37381400
C	2.08287200	3.49684700	-0.42898200
H	1.25219500	5.47940000	-0.38333300
C	1.77484600	2.13727200	-0.42760400
H	0.24508600	0.60868100	-0.36413900
H	3.11839900	3.81469000	-0.46184200
O	2.82339100	1.20689800	-0.54826800
S	3.39238700	0.56807100	0.89167900
O	2.35697400	-0.28988000	1.44674600
O	3.99691400	1.65499600	1.64589800
C	4.69818900	-0.43371700	0.11067600
C	5.84109800	-0.85369000	0.81037000
C	4.57787200	-0.76878800	-1.24152400
C	6.85844300	-1.55637200	0.17713600
C	5.57600200	-1.49784100	-1.88570800
H	3.70809400	-0.44312400	-1.79536100
C	6.70359700	-1.87266700	-1.16600900
H	7.74259200	-1.85509700	0.72475500
N	6.01115800	-0.63629700	2.26324700
O	4.99109100	-0.62647500	2.94239000
O	7.15922400	-0.53002900	2.68042600
H	5.49019100	-1.76614400	-2.93138400
N	7.77328900	-2.63515000	-1.84385900
O	7.60961800	-2.89205000	-3.03346300
O	8.74791700	-2.95667500	-1.17094800

HCy2

C	-7.07930600	-3.09437700	0.05096000
C	-5.74093900	-3.52726600	0.05063500
C	-5.43817600	-4.87924900	0.11858200
C	-6.49372700	-5.80078900	0.18669900
C	-7.82434300	-5.36566300	0.18647900
C	-8.13475400	-4.00475000	0.11862000
C	-5.96920400	-1.21106100	-0.06952600
H	-4.40789400	-5.22896800	0.12041200
H	-6.27573100	-6.86405000	0.24087900
H	-8.62642700	-6.09720400	0.24028200
H	-9.16213200	-3.65335900	0.11827500
N	-7.18493700	-1.69688200	-0.02255300
C	-4.86314800	-2.29215100	-0.03082300

C	-4.01510700	-2.30401700	-1.32322100
H	-3.42064700	-1.39097500	-1.42566700
H	-3.32668000	-3.15691500	-1.31073200
H	-4.65247900	-2.39645900	-2.20854200
C	-3.96715100	-2.17052700	1.22327400
H	-3.28357500	-3.02543600	1.27829900
H	-3.36406200	-1.25773600	1.20577700
H	-4.57125300	-2.16173400	2.13610700
C	-5.80094500	0.22114600	-0.15045900
H	-6.74923500	0.75227100	-0.16183800
C	-4.62867000	0.91292700	-0.20207000
H	-3.69311500	0.36741300	-0.17851200
C	-4.50668600	2.34829500	-0.26401400
C	-3.26993900	2.94610100	-0.26467800
C	-3.04710000	4.37714800	-0.23522700
C	-5.74657100	3.22208500	-0.31194600
H	-6.19651800	3.28442600	0.69090200
H	-6.49956600	2.74231600	-0.94759900
C	-5.46565200	4.63302500	-0.84330300
H	-6.35310300	5.26406400	-0.72129700
H	-5.25078900	4.58294400	-1.91857500
C	-4.26923300	5.25558800	-0.11661900
H	-4.51978600	5.37333100	0.94869900
H	-4.04421100	6.25781600	-0.49877100
O	-2.16128000	2.11173200	-0.28356600
C	-1.78353400	4.87022600	-0.27893800
H	-1.61380700	5.94396000	-0.25978800
C	-0.63926900	3.98838800	-0.32395300
C	-0.88867400	2.60212200	-0.31259700
C	0.69504600	4.42252500	-0.36130800
C	0.14681000	1.67219600	-0.33635700
C	1.74889100	3.51402800	-0.38475600
H	0.90032300	5.48949200	-0.36542500
C	1.45455200	2.15115700	-0.37611200
H	-0.06208800	0.60955700	-0.32242700
H	2.78200600	3.84079700	-0.40470700
O	2.51438800	1.23388400	-0.47113800
S	3.02254700	0.56397400	0.98152000
O	2.02382000	-0.40800200	1.40088300
O	3.45489100	1.65889700	1.83638000
C	4.47014100	-0.27554000	0.26843700
C	5.59203100	-0.62291800	1.03496900
C	4.48468800	-0.55914400	-1.09978000
C	6.71574700	-1.19642800	0.45040600
C	5.59595000	-1.16197200	-1.68659100
H	3.63052800	-0.29415600	-1.70787900
C	6.71576000	-1.47035200	-0.91487600
H	7.56654900	-1.43570900	1.07576100
N	5.62927800	-0.47058500	2.50396700
O	4.56351300	-0.58070200	3.09965000
O	6.72801400	-0.29270500	3.02063200
H	5.58693500	-1.38856900	-2.74729600
C	7.94330900	-2.06028400	-1.56783100
F	8.72256600	-1.09258000	-2.09428100
F	8.69254800	-2.74962900	-0.68620700
F	7.60755900	-2.89513500	-2.57259500

HCy3

C	-6.68741800	-2.81637800	0.09764700
C	-5.37550000	-3.32346000	0.07804100
C	-5.14842400	-4.69115200	0.12349600
C	-6.25344700	-5.55293900	0.18853900
C	-7.55750800	-5.04395900	0.20731000
C	-7.79175100	-3.66700700	0.16209900
C	-5.47468700	-0.99640600	-0.00561800
H	-4.13941400	-5.09780400	0.11046300
H	-6.09516000	-6.62743700	0.22532000
H	-8.39880400	-5.73024600	0.25825100
H	-8.79776200	-3.25877700	0.17674700
N	-6.71510100	-1.41418300	0.04657000
C	-4.43038900	-2.13822500	0.00659900
C	-3.59383300	-2.17905900	-1.29263400
H	-2.94652200	-1.30157500	-1.38591600
H	-2.95736800	-3.07146200	-1.29897500
H	-4.24187700	-2.21948200	-2.17408100
C	-3.52056400	-2.08428300	1.25518800
H	-2.88163700	-2.97407200	1.29143300
H	-2.87177500	-1.20333000	1.24719400
H	-4.11709800	-2.05910900	2.17268000
C	-5.22697900	0.42554700	-0.06303300
H	-6.14361500	1.00950500	-0.04761200
C	-4.01869800	1.05124400	-0.12295300
H	-3.11519500	0.45369900	-0.12917100
C	-3.81725500	2.47876100	-0.15574500
C	-2.54923600	3.00611400	-0.17754700
C	-2.24445900	4.42088500	-0.11369900
C	-5.00623000	3.42187200	-0.14844200
H	-5.42781800	3.48118900	0.86685800
H	-5.80040700	3.00312100	-0.77712800

C	-4.65858800	4.82909500	-0.64911300
H	-5.50549200	5.50524400	-0.48731500
H	-4.47316500	4.79737600	-1.73052400
C	-3.41119200	5.36254300	0.06275100
H	-3.62790800	5.46212800	1.13730500
H	-3.13928400	6.36153300	-0.29668500
O	-1.49073500	2.11157100	-0.25387100
C	-0.95643000	4.84335000	-0.17747400
H	-0.72528600	5.90468800	-0.13123500
C	0.13394900	3.90044600	-0.28314800
C	-0.19392900	2.53071800	-0.30897200
C	1.48917700	4.25996500	-0.34810900
C	0.78570100	1.54600400	-0.39855000
C	2.48867400	3.29554900	-0.43812500
H	1.75512500	5.31316600	-0.32379300
C	2.11642000	1.95234500	-0.46701200
H	0.51665600	0.49698800	-0.41300400
H	3.53740800	3.56480700	-0.48350900
O	3.11605600	0.97803200	-0.63700200
S	3.68647200	0.27039200	0.77106800
O	2.62708800	-0.56321200	1.31857500
O	4.34462300	1.30909200	1.54843100
C	4.93634100	-0.75249300	-0.06853000
C	6.08035300	-1.23223300	0.58740200
C	4.77529800	-1.04363600	-1.42625500
C	7.05847200	-1.94617100	-0.09223600
C	5.73358800	-1.78337700	-2.11402800
H	3.90425000	-0.67362500	-1.94966700
C	6.88565000	-2.23096600	-1.45274200
H	7.93679400	-2.28008200	0.44614000
N	6.28986500	-1.06948000	2.04205700
O	5.28623000	-1.04574200	2.74474600
O	7.44994800	-1.01872700	2.43744200
H	5.59351100	-2.00608900	-3.16624900
C	7.88192400	-2.98164000	-2.16069700
N	8.68501200	-3.58934800	-2.74114700

HCy4

C	7.27484000	-3.03583200	-0.01130100
C	5.94112700	-3.47858800	-0.07347200
C	5.65223700	-4.83160400	-0.17365500
C	6.71704300	-5.74427500	-0.21065000
C	8.04295800	-5.29933200	-0.14822100
C	8.33940500	-3.93733000	-0.04771200
C	6.14580200	-1.16254000	0.08912500
H	4.62574300	-5.18872500	-0.22431000
H	6.51005600	-6.80818700	-0.28961000
H	8.85241700	-6.02405600	-0.17910700
H	9.36305300	-3.57846400	0.00061700
N	7.36627500	-1.63888500	0.08656600
C	5.05109700	-2.25107000	-0.01191600
C	4.14889500	-2.28746400	1.24272600
H	3.54333100	-1.38036300	1.33197700
H	3.46841100	-3.14522700	1.18961000
H	4.74895800	-2.38732100	2.15301900
C	4.20879600	-2.11721000	-1.30145400
H	3.53540800	-2.97636000	-1.39933600
H	3.59807000	-1.20941400	-1.29602100
H	4.85172700	-2.08950100	-2.18697200
C	5.96318000	0.26702000	0.18344600
H	6.90621500	0.80439200	0.24122800
C	4.78478400	0.95030100	0.19756500
H	3.85459000	0.39949000	0.12799800
C	4.65011700	2.38362400	0.27588300
C	3.40960000	2.97288400	0.23920900
C	3.17771400	4.40274100	0.22198600
C	5.88082200	3.26525100	0.38398400
H	6.36873400	3.34509700	-0.59968200
H	6.61240400	2.78164400	1.04139900
C	5.56926500	4.66639700	0.92381900
H	6.45605600	5.30523900	0.84497800
H	5.31374700	4.59947200	1.98929100
C	4.39689000	5.29090400	0.16054600
H	4.68672300	5.42460300	-0.89286100
H	4.15053600	6.28645800	0.54703700
O	2.30733200	2.13100100	0.20562300
C	1.90980600	4.88644800	0.22716300
H	1.73316100	5.95921900	0.21659800
C	0.77117900	3.99646800	0.21919200
C	1.03052400	2.61222800	0.19659000
C	-0.56681600	4.42093100	0.21600100
C	0.00157000	1.67519400	0.17122200
C	-1.61387200	3.50497100	0.19031300
H	-0.77974300	5.48640100	0.22687700
C	-1.31105000	2.14347700	0.17326100
H	0.21809300	0.61423400	0.14775400
H	-2.64932300	3.82472500	0.17677600
O	-2.36462600	1.21951600	0.22085700
S	-2.81529500	0.56233500	-1.26652100

O	-1.80292100	-0.41858300	-1.62813200
O	-3.17389400	1.68157100	-2.12325700
C	-4.32179700	-0.25816000	-0.64130700
C	-5.35205600	-0.70513700	-1.49687700
C	-4.45179100	-0.43059000	0.72464800
C	-6.51686600	-1.21422200	-0.94580200
C	-5.59805000	-1.02816700	1.32318300
H	-3.63524900	-0.12162700	1.35720900
C	-6.66501100	-1.33184800	0.42966100
H	-7.33250500	-1.49571900	-1.59928900
N	-5.25344700	-0.69420600	-2.95466400
O	-4.12881400	-0.57917300	-3.43809100
O	-6.29076700	-0.83450000	-3.60046200
N	-8.02381600	-1.64354900	0.88792100
O	-8.41466100	-1.08462500	1.91467500
O	-8.70822500	-2.38958100	0.18933100
N	-5.60098200	-1.30457100	2.65770800
C	-6.37564600	-2.39789100	3.24109400
H	-5.73416400	-2.93272200	3.94940400
H	-7.27130800	-2.03941600	3.75768500
H	-6.67776000	-3.10709800	2.46756100
C	-4.61934500	-0.69283500	3.54946600
H	-5.01022800	-0.73518700	4.56863800
H	-3.65076100	-1.21270400	3.52692900
H	-4.46739200	0.35875300	3.29557600

HCy5

C	-6.76491400	-3.27225700	0.38626600
C	-5.40390100	-3.62750100	0.38679900
C	-5.02282200	-4.95432500	0.52263500
C	-6.02243100	-5.92924300	0.65856400
C	-7.37581700	-5.57115400	0.65815300
C	-7.76503000	-4.23591600	0.52188700
C	-5.76785600	-1.33860200	0.13967200
H	-3.97427200	-5.24486800	0.52519500
H	-5.74262900	-6.97381700	0.76604400
H	-8.13348800	-6.34304400	0.76527500
H	-8.81084500	-3.94398300	0.51969800
N	-6.95240600	-1.88971300	0.23565200
C	-4.60029000	-2.35059300	0.22222800
C	-3.76807000	-2.38832300	-1.07986600
H	-3.22715200	-1.45090200	-1.24208700
H	-3.03284100	-3.19989100	-1.03055600
H	-4.40950500	-2.56592200	-1.94908200
C	-3.69872700	-2.10522900	1.45396900
H	-2.96932600	-2.91755100	1.55100300
H	-3.14657600	-1.16380500	1.37322100
H	-4.29232900	-2.07329000	2.37313900
C	-5.68514200	0.09308400	-0.03070700
H	-6.66316700	0.56621600	-0.06095700
C	-4.55725400	0.84825100	-0.14436100
H	-3.59047900	0.36190900	-0.09719900
C	-4.52401300	2.28043500	-0.30783300
C	-3.32708800	2.95192600	-0.36433200
C	-3.19466800	4.39191100	-0.45235000
C	-5.81611700	3.07015100	-0.40852200
H	-6.26063700	3.18551200	0.59212600
H	-6.54340100	2.49560500	-0.99349100
C	-5.62807800	4.44827800	-1.05405400
H	-6.55267500	5.03058700	-0.97251500
H	-5.41760100	4.32534700	-2.12433900
C	-4.46846600	5.20031000	-0.39279200
H	-4.72033300	5.38867700	0.66203000
H	-4.30800800	6.18005600	-0.85695800
O	-2.16820500	2.18948900	-0.32504200
C	-1.96487800	4.95730600	-0.55028000
H	-1.86287800	6.03746400	-0.62040000
C	-0.76756100	4.14793000	-0.53593000
C	-0.92915800	2.75466000	-0.40754100
C	0.53652300	4.65967900	-0.62308100
C	0.16346500	1.89417800	-0.35937700
C	1.64641700	3.82054200	-0.58577300
H	0.67415300	5.73313000	-0.71985000
C	1.43770200	2.44737200	-0.45556200
H	0.02165400	0.82611600	-0.24709700
H	2.65603000	4.20838600	-0.65097000
O	2.54142100	1.57815200	-0.48207000
S	3.32134000	1.36244600	0.98820300
O	2.34130600	0.85573800	1.93514500
O	4.11240300	2.55873000	1.23504600
C	4.42065100	0.02499400	0.40702600
C	5.58976800	-0.35100400	1.10299700
C	4.13948200	-0.59278900	-0.79984500
C	6.42160700	-1.32920500	0.58741000
C	4.96704300	-1.59770600	-1.34689100
H	3.28324400	-0.29246700	-1.38655900
C	6.10294400	-1.97736100	-0.60381800
H	7.31045100	-1.61690800	1.13436100
N	5.95261800	0.18825000	2.42145500

O	5.04938300	0.68203000	3.08911100
O	7.12368700	0.07627400	2.77472800
N	6.94362900	-3.13027000	-0.95022600
O	6.37970800	-4.09927600	-1.45900900
O	8.13534100	-3.07331700	-0.65993700
O	4.56814600	-2.04467900	-2.54393400
C	5.49085100	-2.47284000	-3.56184400
H	5.63495200	-3.55215000	-3.51976100
H	5.02593700	-2.18324600	-4.50561700
H	6.45284200	-1.96106000	-3.45346400

HCy6

C	6.67727700	-2.50592500	-0.13687100
C	5.40116300	-3.09401100	-0.06999500
C	5.26143400	-4.47406100	-0.06402400
C	6.41773300	-5.26620200	-0.12517100
C	7.68581900	-4.67674700	-0.19135100
C	7.83208300	-3.28696500	-0.19818400
C	5.35310600	-0.76361900	-0.06506100
H	4.28108300	-4.94334300	-0.01406000
H	6.32762400	-6.34921200	-0.12194400
H	8.56819100	-5.30972600	-0.23850000
H	8.80935200	-2.81656200	-0.24981200
N	6.61652400	-1.10399600	-0.13266300
C	4.38389600	-1.96887700	-0.01636300
C	3.57741200	-2.01858100	1.30158800
H	2.87454600	-1.18324700	1.37872800
H	3.00217300	-2.95028600	1.35237700
H	4.24376300	-1.98477100	2.16959700
C	3.44683900	-2.01463000	-1.24510500
H	2.86998700	-2.94652400	-1.24124300
H	2.73909800	-1.18014400	-1.24735200
H	4.02101000	-1.97656600	-2.17632500
C	5.01717300	0.64060300	-0.04443800
H	5.89437600	1.28004900	-0.09822100
C	3.77355000	1.19179600	0.02776400
H	2.90966300	0.53953900	0.06996500
C	3.48452300	2.60411200	0.03321600
C	2.18674400	3.05341500	0.07410600
C	1.79644400	4.44652300	-0.00357000
C	4.61284300	3.61795000	-0.01923900
H	5.00371200	3.68792900	-1.04613400
H	5.44744100	3.25823100	0.59345800
C	4.19252100	5.00847500	0.47109200
H	4.99315600	5.73203700	0.28071600
H	4.03391100	4.98114700	1.55692400
C	2.89929100	5.45515800	-0.21842600
H	3.08623900	5.55402400	-1.29865900
H	2.57470300	6.44043300	0.13528200
O	1.18713400	2.09911500	0.18796500
C	0.48686600	4.79121400	0.08421300
H	0.19099700	5.83606400	0.02901600
C	-0.54211800	3.78696200	0.22764100
C	-0.13269700	2.44011700	0.26532300
C	-1.91506000	4.06591900	0.31748200
C	-1.04907800	1.40008400	0.38956200
C	-2.85171700	3.04489100	0.44276700
H	-2.24406500	5.10100500	0.28343200
C	-2.40113100	1.72494800	0.48217200
H	-0.71810900	0.36905800	0.41125000
H	-3.91380500	3.25061500	0.50571500
O	-3.33381300	0.69932300	0.68654800
S	-3.89555300	-0.05854100	-0.70969100
O	-2.79711500	-0.84354800	-1.25493100
O	-4.59115900	0.95380900	-1.49089500
C	-5.09303400	-1.12003700	0.15107400
C	-6.21623100	-1.66336700	-0.49489300
C	-4.91790300	-1.37113800	1.51067600
C	-7.15259400	-2.40218100	0.21934100
C	-5.83815400	-2.14032200	2.24085400
H	-4.06129200	-0.94548900	2.01727600
C	-6.96150900	-2.64045500	1.57768300
H	-8.01453700	-2.79152400	-0.30895100
H	-7.69385500	-3.22897400	2.12284100
N	-6.43149700	-1.55198700	-1.94676600
O	-5.43306700	-1.44663900	-2.65286900
O	-7.58912000	-1.61800300	-2.35334500
C	-5.60344100	-2.40378800	3.70783400
H	-5.46039500	-1.46803800	4.26022400
H	-6.44516700	-2.93801500	4.15723600
H	-4.70133900	-3.00919300	3.85825600

HCy7

C	6.66394900	-2.66506600	-0.10437000
C	5.36989200	-3.21405100	-0.05122300
C	5.18752700	-4.58913100	-0.05769600
C	6.31920100	-5.41612200	-0.11767700
C	7.60527200	-4.86555500	-0.17029400

C	7.79449700	-3.48094500	-0.16435900
C	5.39364600	-0.88332600	-0.02746000
H	4.19275700	-5.02799600	-0.01829400
H	6.19577600	-6.49582900	-0.12414300
H	8.46800000	-5.52511900	-0.21677900
H	8.78620300	-3.04067600	-0.20531400
N	6.64643900	-1.26200600	-0.08906500
C	4.38734800	-2.05860500	0.00441200
C	3.56962000	-2.09420700	1.31573100
H	2.89225800	-1.23830000	1.39480400
H	2.96557200	-3.00808800	1.35422700
H	4.23003200	-2.08816100	2.18890000
C	3.45946400	-2.06533600	-1.23201000
H	2.85115900	-2.97701700	-1.23875700
H	2.78071300	-1.20721600	-1.23481000
H	4.04243100	-2.04120000	-2.15822900
C	5.10012600	0.53035300	0.00006000
H	5.99649700	1.14348500	-0.04455700
C	3.87311200	1.11828000	0.06705900
H	2.98970800	0.49223700	0.10167700
C	3.62615200	2.53851700	0.07422800
C	2.34196700	3.02577800	0.11044900
C	1.99261200	4.42939200	0.02756100
C	4.78414600	3.51841300	0.02628800
H	5.18403100	3.57311400	-0.99807700
H	5.60354700	3.13629900	0.64598500
C	4.40207800	4.92270400	0.50929900
H	5.22428500	5.62187900	0.31973800
H	4.23793600	4.90444700	1.59450000
C	3.12535500	5.40408600	-0.18763300
H	3.31939900	5.49206100	-1.26756200
H	2.82861700	6.40021000	0.16011900
O	1.31474400	2.10132600	0.22353100
C	0.69335600	4.81230400	0.10995200
H	0.42823000	5.86511300	0.05008100
C	-0.36474800	3.83884300	0.25399800
C	0.00526300	2.48077100	0.29722000
C	-1.72921500	4.15769900	0.33975300
C	-0.94105300	1.46820000	0.42359500
C	-2.69527900	3.16470500	0.46710300
H	-2.02810200	5.20174000	0.30156100
C	-2.28322100	1.83240000	0.51262300
H	-0.63995100	0.42817500	0.44986000
H	-3.75102300	3.40140200	0.52800400
O	-3.24540600	0.83515600	0.72046100
S	-3.83404600	0.09168200	-0.67156700
O	-2.75327100	-0.70464200	-1.23575800
O	-4.52688800	1.11405200	-1.44257200
C	-5.03114000	-0.95240800	0.20136700
C	-6.18037500	-1.46930000	-0.41835300
C	-4.83894400	-1.22902100	1.55543500
C	-7.11891600	-2.20125800	0.29753700
C	-5.77123300	-1.98755800	2.26523000
H	-3.96776600	-0.83458300	2.06082800
C	-6.92970400	-2.47763600	1.65704600
H	-7.99413900	-2.56232700	-0.23105900
N	-6.42481500	-1.33382500	-1.86735300
O	-5.43844300	-1.27399600	-2.59442900
O	-7.59308100	-1.33766200	-2.24641500
H	-5.58982600	-2.18984900	3.31719900
C	-7.95134000	-3.28034400	2.42359900
H	-8.91058100	-2.75100600	2.47171300
H	-8.13910800	-4.24615100	1.94073600
H	-7.62103100	-3.47204700	3.44828900

HCy8

C	-6.37618900	-3.04320800	0.03337400
C	-5.03711500	-3.47389100	0.03290000
C	-4.73229300	-4.82626400	0.08073700
C	-5.78650000	-5.75059100	0.12915100
C	-7.11781700	-5.31767100	0.12945800
C	-7.43029800	-3.95636700	0.08152700
C	-5.26891600	-1.15615900	-0.05224400
H	-3.70144300	-5.17442300	0.08126400
H	-5.56691600	-6.81421900	0.16702400
H	-7.91886900	-6.05130400	0.16758700
H	-8.45829100	-3.60681400	0.08140100
N	-6.48392000	-1.64492200	-0.01901300
C	-4.16122100	-2.23608800	-0.02450500
C	-3.30555400	-2.22597100	-1.31178200
H	-2.71197900	-1.31052600	-1.39629900
H	-2.61618800	-3.07824500	-1.30945100
H	-3.93764800	-2.30497700	-2.20209100
C	-3.27320600	-2.13270600	1.23678300
H	-2.58817900	-2.98720200	1.28201800
H	-2.67268400	-1.21798900	1.23759400
H	-3.88289500	-2.14014100	2.14592000
C	-5.10235700	0.27725700	-0.11093600
H	-6.05137300	0.80708100	-0.12165800

C	-3.93097200	0.97189500	-0.14354000
H	-2.99442700	0.42801400	-0.12002400
C	-3.81113100	2.40805200	-0.18588900
C	-2.57566800	3.00828800	-0.16222000
C	-2.35630400	4.43953800	-0.11645000
C	-5.05200400	3.28004300	-0.24146300
H	-5.51470900	3.33298400	0.75612900
H	-5.79602300	2.80467300	-0.89080900
C	-4.76669500	4.69603400	-0.75681900
H	-5.65708400	5.32424100	-0.64179400
H	-4.53686800	4.65543100	-1.82937000
C	-3.58181200	5.31474700	-0.00829200
H	-3.84783400	5.42378300	1.05422400
H	-3.35327800	6.32035600	-0.37932300
O	-1.46518200	2.17634600	-0.17146100
C	-1.09323600	4.93541300	-0.13723200
H	-0.92624300	6.00931800	-0.10631300
C	0.05351800	4.05646500	-0.17378700
C	-0.19324900	2.66973400	-0.17859100
C	1.38739600	4.49264700	-0.18833500
C	0.84512000	1.74335200	-0.19002500
C	2.44433900	3.58702000	-0.20954600
H	1.59076500	5.56002200	-0.18325500
C	2.15220100	2.22252400	-0.20984200
H	0.63948200	0.67980300	-0.18472700
H	3.47565900	3.91927800	-0.21975400
O	3.19469600	1.28307900	-0.29446800
S	4.00225100	0.93713500	1.13000300
O	3.03706600	0.43927300	2.10436400
O	4.86718200	2.06580000	1.46459000
C	4.97748200	-0.43522500	0.49847200
C	5.99369500	-0.24569100	-0.46085000
C	4.69166000	-1.68241300	1.05887200
C	6.71013600	-1.38513100	-0.84230000
C	5.42095700	-2.80151500	0.66863500
H	3.90025700	-1.76667700	1.79457600
C	6.41986500	-2.62511200	-0.28128700
H	7.50220300	-1.31452100	-1.57791700
H	5.22723000	-3.78327000	1.08135200
N	7.20650500	-3.79928600	-0.71391000
O	6.93250400	-4.88290700	-0.20298800
O	8.08320900	-3.61785600	-1.55606900
C	6.33244100	1.08924200	-1.07778100
H	5.45550100	1.53536400	-1.55676100
H	6.67847500	1.79578200	-0.31762100
H	7.11579600	0.97009800	-1.83039000

HCy9

C	-6.58663500	-2.40669800	0.13055300
C	-5.32949700	-3.02232800	-0.00834200
C	-5.22611500	-4.40377200	-0.07922600
C	-6.39979600	-5.16925000	-0.01057000
C	-7.64897500	-4.55243700	0.12697900
C	-7.75855100	-3.16113800	0.19973100
C	-5.22074000	-0.69612500	0.08643500
H	-4.26070300	-4.89429800	-0.18502300
H	-6.33810100	-6.25293500	-0.06402400
H	-8.54537900	-5.16501500	0.17849500
H	-8.72076300	-2.66962200	0.30729300
N	-6.48941600	-1.00783600	0.18535000
C	-4.28579500	-1.92125300	-0.05107700
C	-3.53027500	-1.93149300	-1.39991400
H	-2.80972400	-1.11051200	-1.46714500
H	-2.98139700	-2.87350200	-1.51348900
H	-4.22707900	-1.84284400	-2.23966800
C	-3.30483400	-2.04479800	1.13754300
H	-2.75495000	-2.99057500	1.07177600
H	-2.57446200	-1.23006900	1.14753500
H	-3.84205800	-2.03198500	2.09122400
C	-4.84873400	0.69884000	0.11368700
H	-5.70611600	1.35726100	0.22606700
C	-3.59380800	1.22030700	0.01931200
H	-2.75059100	0.54769700	-0.08138600
C	-3.26597800	2.62356000	0.06004400
C	-1.95838200	3.03963800	-0.00810300
C	-1.52740600	4.41825300	0.10475400
C	-4.36310200	3.66403500	0.19153600
H	-4.71311100	3.70662300	1.23452900
H	-5.22956300	3.34920200	-0.40123300
C	-3.92276100	5.05998900	-0.26443300
H	-4.69582600	5.79683200	-0.01903300
H	-3.80426300	5.06817400	-1.35568400
C	-2.59401000	5.44761000	0.39247800
H	-2.74001700	5.51384900	1.48146800
H	-2.25477900	6.43560000	0.06080500
O	-0.98954200	2.06383400	-0.18888400
C	-0.21245800	4.73133200	-0.01583400
H	0.11373900	5.76542200	0.06488300
C	0.78337900	3.70579700	-0.22736400

C	0.33588800	2.37236500	-0.29677400
C	2.15994100	3.95137200	-0.35229200
C	1.21908900	1.31313800	-0.48379600
C	3.06397800	2.91070900	-0.54067400
H	2.51804100	4.97568100	-0.29500000
C	2.57587500	1.60552100	-0.60804500
H	0.85923800	0.29257900	-0.52866100
H	4.12881400	3.09032000	-0.63110000
O	3.47418700	0.56191100	-0.87310600
S	4.05656000	-0.25324400	0.47916900
O	2.95764300	-1.03040100	1.03416200
O	4.80429200	0.71177300	1.27196600
C	5.19796700	-1.31425300	-0.45373200
C	6.33322800	-1.89572500	0.13239100
C	4.96510900	-1.53163700	-1.81393900
C	7.23301400	-2.64019200	-0.62472100
C	5.85162700	-2.30171900	-2.56931200
H	4.10068500	-1.08205100	-2.28332400
C	6.98959600	-2.84829500	-1.98029300
H	8.10382100	-3.05428500	-0.13097200
H	7.68503500	-3.43984400	-2.56736800
N	6.60570200	-1.81641300	1.58027700
O	5.63459900	-1.74685200	2.32651300
O	7.77963500	-1.87355700	1.93619500
H	5.64901000	-2.46184000	-3.62382900

HCy10

C	-6.49171600	-2.86349900	0.01116700
C	-5.17648600	-3.35974400	0.06164700
C	-4.94142600	-4.72400600	0.14971400
C	-6.04172200	-5.59353500	0.18640200
C	-7.34908200	-5.09539100	0.13522100
C	-7.59134400	-3.72189300	0.04687200
C	-5.28917900	-1.03607200	-0.08097500
H	-3.92977800	-5.12227300	0.19074800
H	-5.87712400	-6.66550400	0.25552500
H	-8.18669000	-5.78743300	0.16510800
H	-8.60005900	-3.32205200	0.00716600
N	-6.52767100	-1.46320600	-0.07443800
C	-4.23820500	-2.16837500	0.00392600
C	-3.34577600	-2.23067300	-1.25673900
H	-2.70308000	-1.34909100	-1.34209700
H	-2.70169600	-3.11666200	-1.21572100
H	-3.95480900	-2.29713100	-2.16404000
C	-3.38378600	-2.07896900	1.28910400
H	-2.74349700	-2.96438600	1.37488100
H	-2.73864100	-1.19532600	1.28743000
H	-4.01981500	-2.03455700	2.17897500
C	-5.04993200	0.38596000	-0.16305300
H	-5.97099800	0.96128100	-0.20790300
C	-3.84540200	1.02177100	-0.17968600
H	-2.93738100	0.43363000	-0.12369500
C	-3.65499000	2.44951400	-0.24436200
C	-2.39236800	2.98962900	-0.20718300
C	-2.10488300	4.40917700	-0.17596500
C	-4.85054800	3.37995700	-0.33930400
H	-5.33112800	3.47074200	0.64709500
H	-5.60325600	2.93116300	-0.99756300
C	-4.48579800	4.77206000	-0.86865100
H	-5.34674300	5.44442500	-0.78247500
H	-4.23517900	4.70380600	-1.93513100
C	-3.28827300	5.34362900	-0.10293600
H	-3.57045200	5.47838000	0.95245800
H	-3.00410200	6.33240900	-0.48077500
O	-1.32359200	2.10508500	-0.18584300
C	-0.81913100	4.84301500	-0.17934100
H	-0.60069000	5.90791600	-0.15882000
C	0.28386300	3.90923000	-0.18615800
C	-0.02903600	2.53588700	-0.17968700
C	1.63730100	4.28079700	-0.18605800
C	0.96235600	1.55871900	-0.17542900
C	2.64774000	3.32402100	-0.17722400
H	1.89214200	5.33709400	-0.18785000
C	2.29184900	1.97598300	-0.17614300
H	0.70337300	0.50711600	-0.17217000
H	3.69511100	3.60305900	-0.17284700
O	3.31598300	1.01556300	-0.24429000
S	3.69932000	0.22302800	1.17521000
O	2.64344500	-0.73881600	1.47703200
O	4.12587300	1.20700800	2.16431300
C	5.12816600	-0.64747000	0.53484300
C	6.39110800	-0.07327800	0.68827600
C	4.94364000	-1.88476300	-0.08473300
C	7.50339400	-0.75066300	0.19492000
C	6.05471600	-2.56216600	-0.58028600
H	3.95005500	-2.31079500	-0.16708400
C	7.31165800	-1.97947600	-0.43274300
H	8.50364800	-0.34722700	0.29046700
H	5.96320000	-3.52378100	-1.06945100

N	8.49169800	-2.69787800	-0.95897700
O	8.29779100	-3.78045400	-1.50695500
O	9.58953300	-2.16490400	-0.81520800
H	6.49776500	0.88021200	1.19330700

Cartesian coordinates (angstrom) for the optimized structures of DNs-series probes used for P_k^+ and ω calculations

DNs-AcRh

S	-3.37422000	-1.27060900	1.37438000
O	-2.98818000	-0.16099800	2.23359500
O	-3.79186900	-2.58534200	1.85336600
C	-4.66478300	-0.68874800	0.21247300
C	-6.03142900	-0.65023600	0.54053600
C	-4.28192400	-0.30976600	-1.07713500
C	-6.98805500	-0.25710900	-0.38736900
C	-5.22347400	0.11597600	-2.01301400
H	-3.23772500	-0.36340800	-1.35753500
C	-6.56435700	0.13112200	-1.65137900
H	-8.03720700	-0.25310900	-0.12321200
N	-6.54337400	-0.98794200	1.88791100
O	-5.78388000	-0.81591500	2.83304400
O	-7.70366700	-1.38064900	1.95898500
H	-4.92946800	0.42042200	-3.00992200
N	-7.57462800	0.56745900	-2.63764400
O	-7.16730900	0.89106500	-3.75013400
O	-8.74747500	0.57618100	-2.27613500
N	-2.08958800	-1.60408800	0.30680000
H	-1.97636100	-2.61311500	0.26221500
C	-0.87851000	-0.86257000	0.24948700
C	0.32224500	-1.56464500	0.17698800
C	-0.86840300	0.54118300	0.19229000
C	1.52571800	-0.86942200	0.03248900
H	0.35241200	-2.64879000	0.22737600
C	0.34225600	1.20664100	0.07454100
H	-1.79025300	1.10467600	0.26448100
C	1.56529900	0.52630400	-0.01264900
H	0.34206100	2.29158000	0.03700600
C	3.87695500	-1.05705000	-0.12411500
C	2.86860000	1.27373800	-0.20510300
C	4.95698400	-1.94445300	-0.12593300
C	4.04910800	0.32737600	-0.16986000
C	6.25870600	-1.45081500	-0.21822200
H	4.74729600	-3.00120100	-0.02967100
C	5.36835000	0.80358700	-0.23302300
C	6.45348900	-0.05468300	-0.26205900
H	5.53930100	1.87493400	-0.27565200
H	7.46150600	0.34347700	-0.33566600
C	3.02140300	2.46563400	0.73127700
C	3.06307100	3.63336500	-0.01947800
C	3.11298500	2.51010200	2.11972100
C	3.19689700	4.88873900	0.57229600
C	3.24693000	3.76272400	2.72488100
H	3.08013400	1.60406800	2.71799600
C	3.28922000	4.94104500	1.96238500
H	3.22619700	5.78452900	-0.04065800
H	3.31884300	3.82637200	3.80714900
H	3.39394100	5.89888900	2.46366700
O	2.64409600	-1.65401800	-0.03959100
C	2.94235700	3.28226200	-1.45347500
O	2.93226000	3.99928400	-2.42268700
O	2.83257900	1.91553100	-1.53860000
N	7.39765300	-2.27386100	-0.21419400
H	8.25087800	-1.84547900	0.12820300
C	7.65692200	-3.54087400	-0.72856600
C	6.55452100	-4.28291800	-1.45730300
H	5.91846400	-4.82140500	-0.74501000
H	5.91802900	-3.62228400	-2.05198600
H	7.03432900	-5.02052500	-2.10260300
O	8.77572100	-4.01012800	-0.59315000

DNs-Coum

S	1.92360200	-1.62572200	0.10598000
O	1.30278500	-1.64974200	-1.22102700
O	2.44697400	-2.84163800	0.74130900
C	3.27496500	-0.39325600	0.09302300
C	4.54637100	-0.62299700	-0.47166700
C	3.03996000	0.83251200	0.71491100
C	5.54350800	0.34135900	-0.42354300
C	4.02275100	1.82162300	0.75660200
H	2.08393100	1.02549600	1.18299100
C	5.25835500	1.55596200	0.18533300
H	6.51248900	0.14750200	-0.86261100
N	4.90132200	-1.86951900	-1.16393900
O	3.98702200	-2.56441300	-1.60221000
O	6.09466800	-2.14154600	-1.28256800

H	3.82380200	2.77333700	1.23263600
N	6.30560200	2.58558900	0.22210500
O	6.04726700	3.64771300	0.78745000
O	7.38409300	2.33198700	-0.31419900
N	0.83439300	-0.97678500	1.19791700
H	0.83968700	-1.52067500	2.05965600
C	-0.42951100	-0.42418100	0.83195300
C	-0.55096400	0.57666800	-0.13638200
C	-1.56270400	-0.87336200	1.51573000
C	-1.80744400	1.11355700	-0.42088800
H	0.31989400	0.94192200	-0.66877500
C	-2.79084000	-0.29408000	1.22424100
H	-1.49390200	-1.65397400	2.26682900
C	-2.95842400	0.70265800	0.25863100
H	-1.87902900	1.88257900	-1.18224600
C	-4.35850500	1.24541600	0.03559100
C	-5.16224800	-0.57972000	1.55092800
C	-5.35767200	0.06285200	0.19351100
O	-3.86978600	-0.74339700	1.98897100
H	-5.08571600	-0.70888900	-0.54286300
H	-4.57735400	1.95628500	0.84832300
O	-6.03543500	-0.98670000	2.29060200
C	-4.52168200	1.99669600	-1.28912900
H	-3.83454200	2.84744100	-1.33862500
H	-4.32794100	1.34470000	-2.14747300
H	-5.53479100	2.39657200	-1.38427500
C	-6.82672100	0.43060100	-0.04261300
H	-7.06539800	1.39227100	0.42436900
H	-7.45922800	-0.32008100	0.44619200
C	-7.25174600	0.45452000	-1.52817200
O	-8.14571900	1.29251100	-1.84559700
O	-6.71604900	-0.38838800	-2.30657600

DNs-CV

S	1.48073300	-1.02056700	-0.05188300
O	1.10352700	-1.70508800	-1.29504200
O	1.35221500	-1.70411300	1.23988900
C	3.22507900	-0.50088000	-0.21255500
C	4.29012200	-1.29891300	0.22963100
C	3.51802300	0.74688600	-0.77116400
C	5.60641300	-0.85671900	0.16586000
C	4.83175500	1.19471400	-0.88181600
H	2.69896700	1.37401700	-1.09882500
C	5.85639900	0.38608600	-0.40149000
H	6.41404700	-1.47059900	0.54216900
N	4.10810800	-2.67514700	0.74282500
O	3.34765700	-3.40369300	0.11977400
O	4.78682700	-2.99510000	1.71467900
H	5.06646500	2.15724000	-1.31886900
N	7.25081000	0.85765800	-0.49262300
O	7.43940600	1.96812200	-0.98532700
O	8.13069500	0.11115900	-0.07110500
N	0.79746100	0.49910600	0.04573000
C	-0.51408400	0.63676800	0.06563900
C	-1.47667900	-0.42056500	-0.07154300
C	-2.81202900	-0.15157300	-0.04629400
H	-1.15369600	-1.44292900	-0.21998000
C	-3.36511200	1.18168700	0.11094900
C	-2.41101800	2.28066900	0.25881700
C	-1.02060800	2.01815200	0.24438900
C	-2.86960600	3.59912700	0.42425400
H	-3.93942000	3.77556800	0.43167600
C	-0.12500200	3.08822200	0.41024300
H	0.93735000	2.87487800	0.41827900
C	-0.59054600	4.38535900	0.57307900
H	0.11582400	5.20070200	0.70118200
C	-1.96890200	4.64247000	0.57703200
H	-2.33365600	5.65780700	0.70481300
N	-4.65580100	1.40712400	0.12279000
O	-3.67271100	-1.20013000	-0.18646700
C	-5.01984000	-0.97829300	-0.17247900
C	-5.85900800	-2.06923500	-0.31903400
C	-5.50430500	0.34079500	-0.01581600
C	-7.25035800	-1.86511400	-0.30988700
H	-5.43224400	-3.06081500	-0.43296300
C	-6.90345500	0.52310300	-0.01049300
C	-7.75937600	-0.54859400	-0.15318600
H	-7.28222200	1.53327700	0.10806900
H	-8.83490800	-0.39480500	-0.14170600
N	-8.11343600	-2.93122200	-0.40563700
H	-9.07343000	-2.74373000	-0.65756500
H	-7.75290000	-3.80835300	-0.75377500

Cartesian coordinates (angstrom) for the optimized structures of HCy9, HCy and HCy10 used for TD-DFT calculations

HCy9

C	6.29592300	-2.49147100	-0.06999800
C	5.00350600	-2.99944300	0.08650700
C	4.80468700	-4.37000500	0.18434000
C	5.91851500	-5.21844900	0.12271000
C	7.20534700	-4.69298300	-0.03444500
C	7.41817900	-3.31369200	-0.13406300
C	4.94739800	-0.64486800	-0.03949800
H	3.80590900	-4.78000700	0.30628400
H	5.78063400	-6.29325500	0.19760400
H	8.05921700	-5.36288400	-0.08054300
H	8.42079200	-2.91874800	-0.25632000
N	6.21962100	-1.08031500	-0.14141800
C	7.38189500	-0.20914600	-0.30620900
H	8.28396000	-0.81622300	-0.34237600
H	7.29509600	0.35806200	-1.23721700
H	7.45179100	0.48328000	0.53736800
C	4.01093400	-1.85143300	0.12089200
C	3.26624300	-1.82574900	1.47729000
H	2.54620500	-1.00443600	1.52566800
H	2.71898700	-2.76579000	1.60270500
H	3.96804200	-1.72284600	2.31133300
C	3.01441500	-1.96627900	-1.05763400
H	2.46486800	-2.90932800	-0.97036200
H	2.28848700	-1.14861700	-1.05264400
H	3.53767600	-1.96115300	-2.01930300
C	4.63996000	0.72585400	-0.08702000
H	5.47532100	1.40407600	-0.21690800
C	3.36472800	1.26564900	0.01037700
H	2.52399700	0.59399400	0.12748000
C	3.06054800	2.64278200	-0.05072700
C	1.72853200	3.06181400	0.00835700
C	1.30731300	4.42868700	-0.15221400
C	4.14717300	3.69312500	-0.20511200
H	4.48148500	3.72504000	-1.25251200
H	5.02079900	3.39791200	0.38635000
C	3.69236900	5.08986000	0.23228500
H	4.46239400	5.82590200	-0.02149400
H	3.56762500	5.11133900	1.32265000
C	2.36829900	5.46276300	-0.43886800
H	2.51900300	5.52228000	-1.52676400
H	2.01468500	6.44645700	-0.11305800
O	0.78367300	2.10190200	0.22480700
C	-0.01996900	4.73215300	-0.06310000
H	-0.35153700	5.76097000	-0.17559800
C	-1.00455100	3.71333300	0.16439300
C	-0.54961500	2.38851400	0.29692600
C	-2.38899800	3.95393800	0.25862800
C	-1.41729200	1.32151700	0.51567300
C	-3.27713100	2.91011000	0.47039300
H	-2.75380700	4.97177400	0.15944200
C	-2.77575700	1.60853400	0.59717000
H	-1.04208900	0.31151800	0.62853300
H	-4.34454700	3.08543000	0.54547600
O	-3.67686300	0.57686400	0.88583800
S	-4.13028200	-0.39054000	-0.40787300
O	-3.00986300	-1.27465500	-0.71757000
O	-4.68695600	0.49193700	-1.42903600
C	-5.44241900	-1.26757300	0.47299900
C	-6.54357500	-1.83168100	-0.19534500
C	-5.38684300	-1.35094900	1.86444700
C	-7.58339900	-2.42762400	0.51048700
C	-6.41763200	-1.97541800	2.57215100
H	-4.55531400	-0.91733800	2.40386100
C	-7.51723700	-2.50291400	1.90084000
H	-8.41911100	-2.84935700	-0.03522200
H	-8.32177600	-2.98098400	2.44982000
N	-6.62279200	-1.89216200	-1.65890500
O	-5.56711000	-2.03335600	-2.27710300
O	-7.73634200	-1.84141800	-2.17763600
H	-6.35365700	-2.03500100	3.65380800

HCy

C	-4.82201200	0.11430900	0.02037100
C	-4.18886500	-1.13467800	0.00030600
C	-4.94481800	-2.29822700	-0.00122700
C	-6.34470700	-2.20107100	0.01940800
C	-6.96267500	-0.94770900	0.04190000
C	-6.21097800	0.23407600	0.04362800
C	-2.57607900	0.59947200	0.00466800
H	-4.46274500	-3.27239200	-0.01726800
H	-6.94912900	-3.10372100	0.01894600
H	-8.04725200	-0.88132500	0.05977300
H	-6.70500900	1.19976100	0.06504200

N	-3.84426700	1.12375800	0.01449500
C	-4.13260200	2.54923400	0.02293500
H	-3.65301900	3.03792300	-0.83162700
H	-5.20855200	2.70285700	-0.04708300
H	-3.76767100	3.01168000	0.94683800
C	-2.68053500	-0.93787200	-0.01611000
C	-2.07709100	-1.53924800	-1.30719200
H	-0.99275500	-1.40152600	-1.34948700
H	-2.28315900	-2.61482200	-1.33969100
H	-2.51745000	-1.08023100	-2.19879700
C	-2.04463800	-1.58012000	1.23869100
H	-2.25405700	-2.65549300	1.24394200
H	-0.95905800	-1.44798700	1.25586100
H	-2.45881900	-1.14725700	2.15554600
C	-1.45374100	1.40689600	0.01664100
H	-1.62512300	2.47760500	0.04894000
C	-0.10992700	0.95955100	-0.00061100
H	0.06744900	-0.10850300	-0.01852600
C	1.01299700	1.76781800	0.01615000
C	2.32093900	1.18623500	0.03359300
C	3.51139500	1.91692700	0.11704300
C	0.93394300	3.28535500	0.05317000
H	0.77383100	3.62282500	1.08866000
H	0.06099000	3.62296100	-0.51593200
C	2.19413700	3.95888400	-0.50514800
H	2.12236700	5.04459000	-0.37559000
H	2.26510000	3.76428400	-1.58354800
C	3.45071200	3.42601700	0.18854100
H	3.44965300	3.74809300	1.24083100
H	4.35568000	3.84856900	-0.26351200
O	2.36214400	-0.17619600	-0.03129200
C	4.72371800	1.21752000	0.13591900
H	5.65509100	1.77720200	0.19550800
C	4.77314000	-0.17284100	0.07947100
C	3.53582500	-0.89040900	-0.00727900
C	5.97275000	-0.95335900	0.09620900
C	3.46383800	-2.25680300	-0.07201100
C	5.93044600	-2.31485100	0.03359300
H	6.92240200	-0.42626300	0.16120000
C	4.66959800	-3.06345500	-0.05459100
H	2.50144900	-2.75541000	-0.13838100
H	6.84512800	-2.90257200	0.04716300
O	4.65109400	-4.31343700	-0.11104400

HCy10

C	-6.18259100	-2.92777100	0.08515700
C	-4.84225900	-3.31658500	0.01710600
C	-4.51056400	-4.66486500	0.02559500
C	-5.54154400	-5.61146200	0.09949700
C	-6.87849900	-5.20449500	0.16129000
C	-7.22476300	-3.84892500	0.15417400
C	-5.01569700	-0.96392600	-0.02426200
H	-3.47305900	-4.98341700	-0.02541300
H	-5.29997400	-6.67042400	0.10689600
H	-7.66693000	-5.94982200	0.21507200
H	-8.26496000	-3.54530600	0.19801400
N	-6.24375900	-1.51426600	0.06685900
C	-7.49217200	-0.75638600	0.12933600
H	-8.31801900	-1.44015300	0.31477100
H	-7.44669700	-0.03123700	0.94586300
H	-7.66364600	-0.23251200	-0.81582000
C	-3.96287900	-2.08193200	-0.06215800
C	-3.17237400	-2.07376000	-1.39303400
H	-2.54467100	-1.18290300	-1.48301800
H	-2.52256200	-2.95430600	-1.42893500
H	-3.84840000	-2.11008100	-2.25352000
C	-3.00740400	-2.02432500	1.15394000
H	-2.37694700	-2.91951200	1.15431300
H	-2.35202000	-1.15030200	1.11505500
H	-3.56680600	-1.99799700	2.09470300
C	-4.84290700	0.42980200	-0.08769600
H	-5.74601900	1.02894600	-0.08099300
C	-3.62166700	1.08700600	-0.15132600
H	-2.71268600	0.49967500	-0.13123200
C	-3.45655700	2.48730400	-0.22156900
C	-2.17004700	3.03393700	-0.21875000
C	-1.89499600	4.44670800	-0.20843200
C	-4.64573000	3.43065400	-0.28307300
H	-5.06447600	3.56125300	0.72569000
H	-5.43773800	2.97446700	-0.88690800
C	-4.28651600	4.79962200	-0.87133200
H	-5.14156800	5.47772200	-0.78081800
H	-4.06652800	4.69326900	-1.94149500
C	-3.06963600	5.39295800	-0.15734600
H	-3.32507000	5.58105300	0.89580700
H	-2.77983200	6.35607000	-0.59013200
O	-1.12335200	2.15921900	-0.21908900
C	-0.59892100	4.87354500	-0.22447900
H	-0.37656200	5.93732500	-0.22668000

C	0.49361700	3.94296700	-0.22325600
C	0.17813700	2.57185300	-0.21160700
C	1.85255600	4.31280100	-0.22647900
C	1.15773800	1.58211000	-0.19942100
C	2.84960900	3.34875700	-0.21171000
H	2.11054000	5.36742100	-0.23818500
C	2.48507900	1.99656800	-0.19804500
H	0.88990600	0.53223000	-0.20108100
H	3.89843900	3.62350100	-0.21726000
O	3.49889400	1.02977800	-0.25261800
S	3.97361100	0.35036300	1.20226200
O	2.91486300	-0.54397300	1.66820700
O	4.46038000	1.41584800	2.07671400
C	5.34185800	-0.60076700	0.55584000
C	6.61142200	-0.02024800	0.53475100
C	5.10237600	-1.89981100	0.10375200
C	7.67811300	-0.76313300	0.03730300
C	6.16925200	-2.64166000	-0.39492800
H	4.10833800	-2.33041200	0.14113900
C	7.43393900	-2.05704200	-0.41879400
H	8.67750500	-0.34831200	0.00649000
H	6.02320300	-3.65233600	-0.75429600
N	8.56345500	-2.84267700	-0.94616200
O	8.33427600	-3.98461000	-1.34554300
O	9.67550400	-2.31444300	-0.96024500
H	6.77125100	0.98735400	0.90124000

References

1. Zhang, J.; Shibata, A.; Ito, M.; Shuto, S.; Ito, Y.; Mannervik, B.; Abe, H.; Morgenstern, R., Synthesis and characterization of a series of highly fluorogenic substrates for glutathione transferases, a general strategy. *J. Am. Chem. Soc.* **2011,** *133*, 14109-14119.
2. Zhang, X. X.; Qi, H.; Lu, M. H.; Yang, S. Q.; Li, P.; Piao, H. L.; Han, K. L., Semi-Quantitatively Designing Two-Photon High-Sensitive Fluo-rescent Probes for Glutathione S-Transferases with Negligible Background Noise. **2018**,
3. Weigend, F.; Ahlrichs, R., Balanced basis sets of split valence, triple zeta valence and quadruple zeta valence quality for H to Rn: Design and assessment of accuracy. *Phys. Chem. Chem. Phys.* **2005,** *7*, 3297-3305.
4. Parr, R. G.; Von Szentpaly, L.; Liu, S. B., Electrophilicity index. *J. Am. Chem. Soc.* **1999,** *121*, 1922-1924.
5. Weigend, F., Accurate Coulomb-fitting basis sets for H to Rn. *Phys. Chem. Chem. Phys.* **2006,** *8*, 1057-1065.
6. Hellweg, A.; Hattig, C.; Hofener, S.; Klopper, W., Optimized accurate auxiliary basis sets for RI-MP2 and RI-CC2 calculations for the atoms Rb to Rn. *Theor. Chem. Acc.* **2007,** *117*, 587-597.
7. Grimme, S.; Antony, J.; Ehrlich, S.; Krieg, H., A consistent and accurate ab initio parametrization of density functional dispersion correction (DFT-D) for the 94 elements H-Pu. *J. Chem. Phys.* **2010,** *132*, 154104-154101-154104-154119.
8. Rassolov, V. A.; Ratner, M. A.; Pople, J. A.; Redfern, P. C.; Curtiss, L. A., 6-31G*basis set for third-row atoms. *J. Comput. Chem.* **2001,** *22*, 976-984.
9. Le Guennic, B.; Jacquemin, D., Taking up the cyanine challenge with quantum tools. *Acc. Chem. Res.* **2015,** *48*, 530-537.
10. Lu, T.; Chen, F., Multiwfn: a multifunctional wavefunction analyzer. *J. Comput. Chem.* **2012,** *33*, 580-592.
11. Gaussian 09, R. D.; M. J. Frisch, G. W. T., H. B. Schlegel, G. E. Scuseria, ; M. A. Robb, J. R. C., G. Scalmani, V. Barone, B. Mennucci, ; G. A. Petersson, H. N., M. Caricato, X. Li, H. P. Hratchian, ; A. F. Izmaylov, J. B., G. Zheng, J. L. Sonnenberg, M. Hada, ; M. Ehara, K. T., R. Fukuda, J. Hasegawa, M. Ishida, T. Nakajima, ; Y. Honda, O. K., H. Nakai, T. Vreven, J. A. Montgomery, Jr., ; J. E. Peralta, F. O., M. Bearpark, J. J. Heyd, E. Brothers, ; K. N. Kudin, V. N. S., T. Keith, R. Kobayashi, J. Normand, ; K. Raghavachari, A. R., J. C. Burant, S. S. Iyengar, J. Tomasi, ; M. Cossi, N. R., J. M. Millam, M. Klene, J. E. Knox, J. B. Cross, ; V. Bakken, C. A., J. Jaramillo, R. Gomperts, R. E. Stratmann, ; O. Yazyev, A. J. A., R. Cammi, C. Pomelli, J. W. Ochterski, ; R. L. Martin, K. M., V. G. Zakrzewski, G. A. Voth, ; P. Salvador, J. J. D., S. Dapprich, A. D. Daniels, ; O. Farkas, J. B. F., J. V. Ortiz, J. Cioslowski, ; and D. J. Fox, G., Inc., Wallingford CT, 2013., **2013**,
12. Marenich, A. V.; Cramer, C. J.; Truhlar, D. G., Universal solvation model based on solute electron density and on a continuum model of the solvent defined by the bulk dielectric constant and atomic surface tensions. *J. Phys. Chem. B* **2009,** *113*, 6378-6396.
13. Kendall, R. A.; Dunning, T. H.; Harrison, R. J., Electron-Affinities of the 1st-Row Atoms Revisited - Systematic Basis-Sets and Wave-Functions. *J. Chem. Phys.* **1992,** *96*, 6796-6806.
14. Woon, D. E.; Dunning, T. H., Gaussian-Basis Sets for Use in Correlated Molecular Calculations .3. The Atoms Aluminum through Argon. *J. Chem. Phys.* **1993,** *98*, 1358-1371.
15. Dunning, T. H., Gaussian-Basis Sets for Use in Correlated Molecular Calculations .1. The Atoms Boron through Neon and Hydrogen. *J. Chem. Phys.* **1989,** *90*, 1007-1023.
16. Gaussian 16, R. A.; M. J. Frisch, G. W. T., H. B. Schlegel, G. E. Scuseria, ; M. A. Robb, J. R. C., G. Scalmani, V. Barone, ; G. A. Petersson, H. N., X. Li, M. Caricato, A. V. Marenich, ; J. Bloino, B. G. J., R. Gomperts, B. Mennucci, H. P. Hratchian, ; J. V. Ortiz, A. F. I., J. L. Sonnenberg, D. Williams-Young, ; F. Ding, F. L., F. Egidi, J. Goings, B. Peng, A. Petrone, ; T. Henderson, D. R., V. G. Zakrzewski, J. Gao, N. Rega, ; G. Zheng, W. L., M. Hada, M. Ehara, K. Toyota, R. Fukuda, ; J. Hasegawa, M. I., T. Nakajima, Y. Honda, O. Kitao, H. Nakai, ; T. Vreven, K. T., J. A. Montgomery, Jr., J. E. Peralta, ; F. Ogliaro, M. J. B., J. J. Heyd, E. N. Brothers, K. N. Kudin, ; V. N. Staroverov, T. A. K., R. Kobayashi, J. Normand, ; K. Raghavachari, A. P. R., J. C. Burant, S. S. Iyengar, ; J. Tomasi, M. C., J. M. Millam, M. Klene, C. Adamo, R. Cammi, ; J. W. Ochterski, R. L. M., K. Morokuma, O. Farkas, ; J. B. Foresman, a. D. J. F., Gaussian, Inc., Wallingford CT, 2016.,
17. Yuan, L.; Lin, W.; Zhao, S.; Gao, W.; Chen, B.; He, L.; Zhu, S., A unique approach to development of near-infrared fluorescent sensors for in vivo imaging. *J. Am. Chem. Soc.* **2012,** *134*, 13510-13523.
18. Roy, R. K.; Hirao, K.; Pal, S., On non-negativity of Fukui function indices. II. *J. Chem. Phys.* **2000,** *113*, 1372-1379.
19. Roy, R. K.; Hirao, K.; Krishnamurty, S.; Pal, S., Mulliken population analysis based evaluation of condensed Fukui function indices using fractional molecular charge. *J. Chem. Phys.* **2001,** *115*, 2901-2907.
20. Cheng, T.; Zhao, Y.; Li, X.; Lin, F.; Xu, Y.; Zhang, X.; Li, Y.; Wang, R.; Lai, L., Computation of octanol-water partition coefficients by guiding an additive model with knowledge, http://www.sioc-ccbg.ac.cn/?p=42&software=xlogp3. *J Chem Inf Model* **2007,** *47*, 2140-2148.
21. Tetko, I. V.; Gasteiger, J.; Todeschini, R.; Mauri, A.; Livingstone, D.; Ertl, P.; Palyulin, V. A.; Radchenko, E. V.; Zefirov, N. S.; Makarenko, A. S.; Tanchuk, V. Y.; Prokopenko, V. V., Virtual computational chemistry laboratory--design and description. *J. Comput. Aided Mol. Des.* **2005,** *19*, 453-463.
22. VCCLAB, Virtual Computational Chemistry Laboratory, http://www.vcclab.org. **2005**,
23. Kamiya, M.; Asanuma, D.; Kuranaga, E.; Takeishi, A.; Sakabe, M.; Miura, M.; Nagano, T.; Urano, Y., beta-Galactosidase fluorescence probe with improved cellular accumulation based on a spirocyclized rhodol scaffold. *J. Am. Chem. Soc.* **2011,** *133*, 12960-12963.
